# Real-life implementation of a G6PD deficiency screening qualitative test into routine vivax malaria diagnostic units in the Brazilian Amazon (SAFEPRIM study)

**DOI:** 10.1371/journal.pntd.0009415

**Published:** 2021-05-18

**Authors:** Jose Diego Brito-Sousa, Felipe Murta, Sheila Vitor-Silva, Vanderson S. Sampaio, Maxwell O. Mendes, Marcelo A. M. Brito, Talita S. B. Batista, Alicia P. C. Santos, Leonardo L. G. Marques, Laila R. A. Barbosa, Marly M. Melo, Djane C. Baia-da-Silva, Alexandre V. Silva-Neto, Thalie C. Santos, Brenda K. A. Souza, Erick F. G. Figueiredo, Emanuelle L. Silva, Sheila Rodovalho, Theresa H. Nakagawa, Ana Ruth Arcanjo, André M. Siqueira, Gisely C. Melo, Judith Recht, Gonzalo J. Domingo, Quique Bassat, Germana Bancone, Wuelton M. Monteiro, Marcus V. G. Lacerda

**Affiliations:** 1 Instituto de Pesquisa Clínica Carlos Borborema, Fundação de Medicina Tropical Dr Heitor Vieira Dourado, Manaus, Brazil; 2 Escola Superior de Ciências da Saúde, Universidade do Estado do Amazonas, Manaus, Brazil; 3 Escola de Enfermagem de Manaus, Universidade Federal do Amazonas, Manaus, Brazil; 4 Fundação de Vigilância em Saúde do Amazonas—FVS-AM, Manaus, Brazil; 5 Pan American Health Organization–PAHO, World Health Organization, Brasilia, Brazil; 6 Laboratório Central de Saúde Pública do Amazonas–LACEN/AM, Manaus, Brazil; 7 Fundação Oswaldo Cruz, Instituto Nacional de Infectologia–INI, Rio de Janeiro, Brazil; 8 Independent consultant, North Bethesda, Maryland, United States of America; 9 Diagnostics Program, PATH, Seattle, Washington, United States of America; 10 Institut de Salut Global de Barcelona (ISGlobal), Hospital Clínic—Universitat de Barcelona, Barcelona, Spain; 11 Centro de Investigação em Saúde de Manhiça (CISM), Maputo, Mozambique; 12 Institució Catalana de Recerca i Estudis Avançats (ICREA), Pg. Lluís Companys, Barcelona, Spain; 13 Pediatric Infectious Diseases Unit, Pediatrics Department, Hospital Sant Joan de Déu (University of Barcelona), Barcelona, Spain; 14 Consorcio de Investigación Biomédica en Red de Epidemiología y Salud Pública (CIBERESP), Madrid, Spain; 15 Shoklo Malaria Research Unit, Mahidol–Oxford Tropical Medicine Research Unit, Faculty of Tropical Medicine, Mahidol University, Mae Sot, Thailand; 16 Centre for Tropical Medicine and Global Health, Nuffield Department of Medicine, University of Oxford, Oxford, United Kingdom; 17 Fundação Oswaldo Cruz, Instituto Leônidas e Maria Deane—ILMD, Manaus, Amazonas, Brazil; Eijkman-Oxford Clinical Research Unit, INDONESIA

## Abstract

**Background:**

Glucose-6-phosphate dehydrogenase (G6PD) deficiency greatly hinders *Plasmodium vivax* malaria radical cure and further elimination due to 8-aminoquinolines-associated hemolysis. Although the deleterious health effects of primaquine in G6PD deficient individuals have been known for over 50 years, G6PD testing is not routinely performed before primaquine treatment in most *P*. *vivax* endemic areas.

**Method/Principal findings:**

The qualitative CareStart G6PD screening test was implemented in 12 malaria treatment units (MTUs) in the municipality of Rio Preto da Eva, Western Brazilian Amazon, a malaria endemic area, between February 2019 and early January 2020. Training materials were developed and validated; evaluations were conducted on the effectiveness of training health care professionals (HCPs) to perform the test, the interpretation and reliability of routine testing performed by HCPs, and perceptions of HCPs and patients. Most HCPs were unaware of G6PD deficiency and primaquine-related adverse effects. Most of 110 HCPs trained (86/110, 78%) were able to correctly perform the G6PD test after a single 4-hour training session. The test performed by HCPs during implementation showed 100.0% (4/4) sensitivity and 68.1% (62/91) specificity in identifying G6PD deficient patients as compared to a point-of-care quantitative test (Standard G6PD).

**Conclusions/Significance:**

G6PD screening using the qualitative CareStart G6PD test performed by HCPs in MTUs of an endemic area showed high sensitivity and concerning low specificity. The amount of false G6PD deficiency detected led to substantial loss of opportunities for radical cure.

## Author summary

Glucose 6-phosphate dehydrogenase deficiency (G6PDd) has greatly impacted the treatment of *Plasmodium vivax* malaria because of the red blood cell destruction in what is known as hemolysis. Primaquine, used to clear dormant liver parasites that cause relapses of the disease, is a well-known trigger that may lead to life-threatening complications in patients with this condition. Although there are several G6PDd diagnostic tests available to guide the decision of weekly or daily primaquine treatment, they are not yet routinely used: questions on how, when, where and who is going to perform the test remain unanswered. This study revealed that, although G6PDd was not previously known by most of the healthcare workers, they were able to perform the test after a single training session. The test performed well in the field, differentiating patients that cannot use daily primaquine from the others, but some expected limitations require further action to be taken into consideration. This research provides an important overall understanding that may aid policy makers in the process of recommending proven interventions, such as G6PDd screening, to implement them pragmatically.

## Introduction

The radical cure for *Plasmodium vivax* malaria is achieved by combination therapy using blood schizontocidal agents along with an 8-aminoquinoline drug, of which primaquine (PQ) is the most used worldwide. The use of PQ is associated with acute hemolytic anemia (AHA) in a dose-dependent manner in patients presenting glucose-6-phosphate dehydrogenase (G6PD) deficiency (G6PDd); G6PDd is an X-linked genetic condition estimated to affect 8% of the world’s population living in malaria endemic areas [1]. The red blood cells of G6PD deficient individuals lack appropriate antioxidant defense mechanisms and become prone to early destruction exacerbated by oxidative stress induced by PQ [2,3].

Although AHA can lead to severe anemia and acute kidney injury [4,5], with an estimated impact on hospitalization costs of US$ 6 million per year to the Brazilian public health budget [6], there is currently no routine G6PDd screening performed in vivax malaria patients prior to treatment in Brazil, where all patients are given standard short-course PQ treatment (0.5 mg/kg/day for 7 days) [7]. With a prevalence ranging from 4–10% in the Amazon region, mostly the African A- variant, the condition is often suspected after a hemolytic crisis during treatment [5,8]. On the other hand, concerns regarding AHA in the absence of available G6PD testing in areas where more severe G6PD variants prevail, often result in hesitancy to prescribe PQ, leading to a continuous cycle of relapses and increased morbidity and mortality in vivax malaria patients [7].

Qualitative G6PD rapid diagnostic tests can reliably identify patients with enzyme activity below 30%, thus missing those with intermediate activity, as opposed to quantitative assays [9–14]. Their recent availability makes it possible to incorporate G6PDd screening into malaria case management. This does represent a significant change in workflow to frontline workers, with operational challenges that need to be identified and addressed. A real-world study is of paramount importance to answer this knowledge gap, especially in a malaria pre-elimination scenario where the use of single dose tafenoquine, an 8-aminiquinoline anti-relapse drug with similar efficacy to primaquine, is imminent [15–17]. The present study aimed at evaluating the routine implementation of the CareStart G6PD test at malaria treatment units (MTUs) of a municipality in the countryside of the Brazilian Amazon, assessing the reliability of interpretation by health care professionals (HCPs), the test performance in the field, and the perception about the test by HCPs and users pre- and post-implementation.

## Methods

### Ethics statement

This protocol was approved by the Ethics Review Board at the *Fundação de Medicina Tropical Dr Heitor Vieira Dourado* in Manaus, Brazil (Study ID: 92012818.1.0000.0005). HCPs and patients gave approval and signed informed consent forms for the qualitative interviews.

### Study site

**Fig 1:**
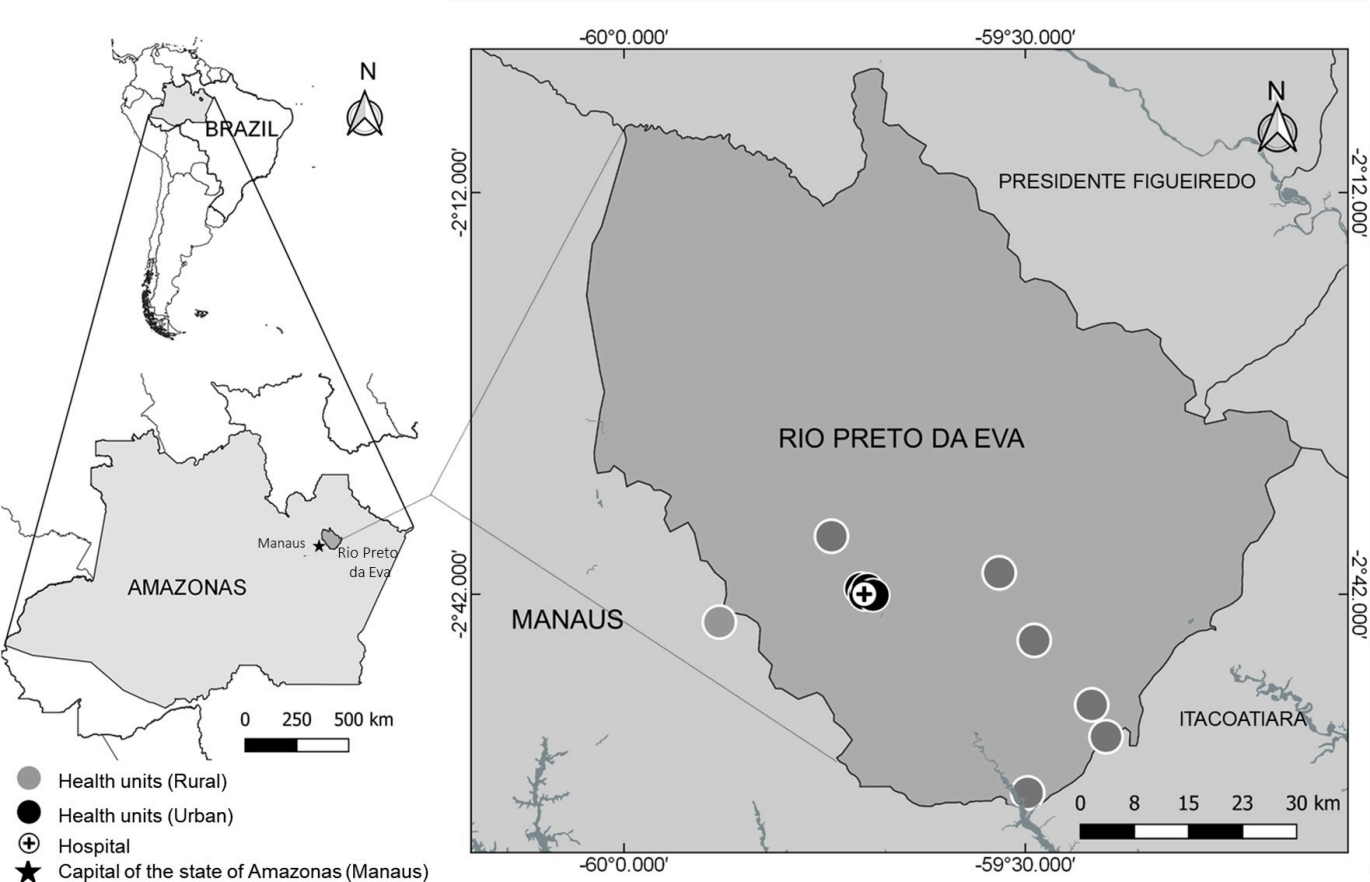
Study location and malaria treatment units (MTU). Rio Preto da Eva is located 78 km from Manaus, capital of the State of Amazonas, Western Brazilian Amazon. The test was implemented in 5 urban (including the local hospital, black sign and black circle, respectively) and 7 rural health posts (filled grey circles). This map was created using the base from the Brazilian Institute of Geography and Statistics (https://portaldemapas.ibge.gov.br/portal.php#homepage).

G6PD screening was implemented in all (*n* = 12) malaria treatment units (MTUs) in the municipality of Rio Preto da Eva, Brazilian state of Amazonas, between February 2019 and January 2020 in urban and rural areas including the local reference hospital (Fig 1). The municipality, located 78 km away from the capital city Manaus, has an estimated population of 32,577 inhabitants with 1,025 malaria cases reported in 2018, almost all *P*. *vivax* mono-infections [18]. A formal agreement was signed between the local, state and federal malaria control programs for implementation of routine G6PD testing in the entire municipality for 18 months.

### Study design

**Fig 2:**
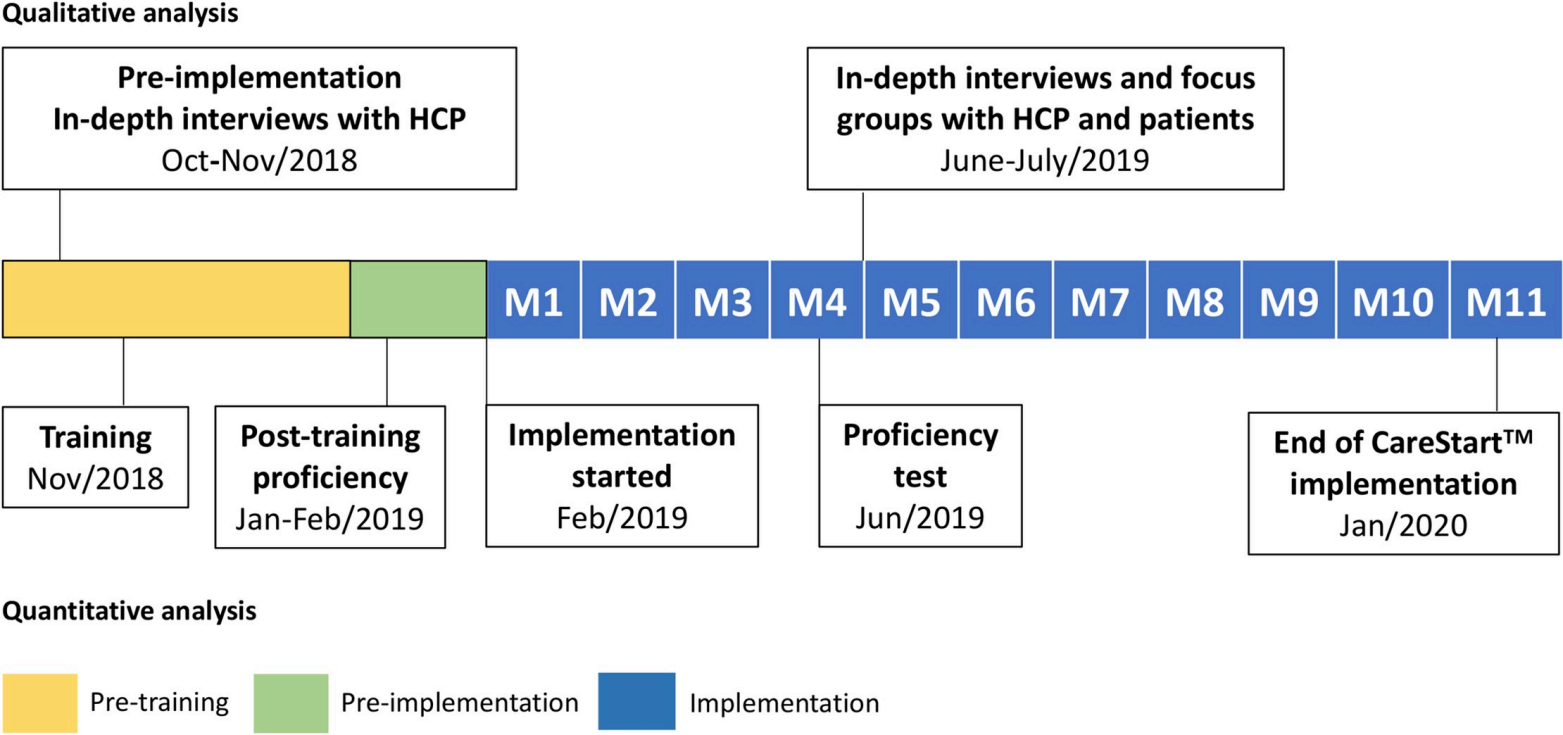
Timeline of events. Timeline of events is shown consisting of three phases: 1) pre-training (before any study intervention, yellow), pre-implementation (after training but before implementation, green), and implementation period (actual implementation of the test, blue). Each M corresponds to a month of implementation, where M1 is March 2019, M10 is December 2019, and M11 is early January 2020.

The SAFEPRIM mixed-methods study aimed at evaluating the operational aspects of implementation of the CareStart (AcessBio, New Jersey) rapid diagnostic test (RDT) for G6PDd screening prior to PQ use in a malaria endemic area. Specific aims of the study were to develop training materials, evaluate effectiveness of HCP training and implementation and reliability of the test performed by HCPs, and evaluate the perceptions of HCPs and patients over the implementation process (Fig 2). Methods and results sessions have been divided into quantitative (A) and qualitative (B) to facilitate comprehension of the study design.

### A. Quantitative methods

#### A.1. HCPs training and proficiency assessment

Before the actual implementation of the test, training and evaluation of proficiency were conducted. Some of the trainers were from the state level malaria reference laboratory (*Laboratório Central do Amazonas*—LACEN), guaranteeing the sustainability of the actions post-study. They all reviewed and guided the development of all materials used. Theoretical and practical training sessions of ~4 hours covered G6PDd and testing, malaria treatment, pharmacovigilance (PV) of PQ-associated hemolysis, CareStart G6PD test procedure, interpretation, result documentation, and case-scenarios (see training session slides—S1 File), a locally recorded video of the CareStart G6PD procedure (S2 File), job aids handed to every HCP in a training pack including a guideline summarizing the test procedure and interpretation (S3 File), a quick guidance wall poster (S4 File) provided to every MTU, and a modified SIVEP-malaria reporting form adapted from the nationally used version to add G6PD data and PV of PQ-associated hemolysis (S5 File). All job aids were created based on WHO guidelines on how to use a G6PD RDT [19] and results from the qualitative analysis before the training. A supervised practice round followed the theory session, where groups of 5–8 HCPs per instructor used blood samples of known G6PD activity in order to practice sample collection.

HCPs proficiencies on result interpretation post-training was measured using a panel of 10 tests (4 normal, 4 deficient and 2 intermediate results, the latter also interpreted as deficient) using recombinant G6PD (r-G6PD) lyophilized controls [20] at their respective MTUs. Briefly, a satisfactory result was obtained when 90% of correct answers were achieved on the panel of tests; this threshold was chosen in line with LACEN guidelines for microscopists training. If an individual failed the test, an additional round of training and evaluation was carried out individually. A training certificate was provided to all approved participants.

Proficiency assessments at four months post-implementation were conducted with HCPs who had used or not the test since some MTUs did not report any malaria case in the after the implementation and accepted to participate in this follow-up. Assessment was carried out using a panel of five tests (two G6PD normal, two G6PD deficient and one intermediate) and based on local refresher training schedule for malaria workers.

#### A.2. G6PD testing, results recording and PV

All *P*. *vivax* malaria patients were screened for G6PDd in MTUs before PQ prescription. Occasionally, when the patient was unable to go to the health post, HCPs performed testing at their household before providing treatment. The CareStart G6PD test is based on a colorimetric change after incubation of blood with two drops of buffer on the test platform. A result can be observed after 10 minutes, with a distinct purple color interpreted as normal ("can use daily PQ") whilst a faint or no color change is interpreted as deficient ("cannot use daily PQ"), according to the current manufacturer’s label. The test does not have a control line. Intermediate results cannot be distinguished clearly, and thus faint color changes were also considered deficient. An invalid result was considered when blood did not run correctly through the testing window. Chloroquine for three days plus seven days PQ (0.5mg/kg/day) or weekly primaquine (0.75mg/kg/week for eight weeks) were used for G6PD normal and deficient patients, respectively, as per national guidelines [21]. Treatment supervision was solely dependent on municipality’s capability. A test result colored card was given by HCPs to the patient to bring it in case of a new malaria episode or to a medical appointment; G6PD normal patients received purple cards while G6PD deficient patients were given gray cards (S6 File). HCPs were trained to advise all patients to return on the fifth day of treatment to monitor signs and symptoms of AHA, especially dark urine and jaundice [5]. If present, patients should be referred to the reference hospital in Manaus, the state referral unit for tropical and infectious diseases.

#### A.3. Test performance in field conditions

All deficient and normal patients (approximate 1:2 ratio) on CareStart RDT were drawn from the database, reported up to the time of this analysis, to be confirmed by point-of-care enzymatic activity quantification [11] at a different time in patient households, according to manufacturer’s instructions (Standard G6PD, SD Biosensor, Korea) in an exploratory analysis. This test showed a sensitivity of 100% and specificity of 97% compared to a spectrophotometric assay to diagnose G6PD deficiency (< 30% normal) [11], For quality assurance of the quantitative test, normal and deficient controls provided by the manufacturer were used. Study field supervisors were trained on how to perform the test and control runs in the field by professionals with extensive experience with the biosensor. Blood sample from finger-prick was collected on filter papers [13]. Samples were genotyped for the three most common single nucleotide polymorphisms (SNPs) for G6PDd in the Brazilian Amazon [8,13]: rs1050828, rs1050829 and rs5030868, corresponding to G6PD African A-^(G202A, A376G)^, G6PD African A+^(A376G)^ and G6PD Mediterranean^(C563T)^, respectively.

#### A.4. Quality control and oversight

In order to ensure adequate storage conditions and following good laboratory practices, test kits were kept as per manufacturer’s instructions (up to 30°C protected from direct exposure to sunlight) at the *Fundação de Medicina Tropical Dr Heitor Vieira Dourado* in Manaus, Amazonas, until their distribution to the Municipality Malaria Department where they were kept at 24° C. Test kits were stored protected from direct exposure to sunlight when air conditioning was not available. Field supervisors oversaw procedures involving test application, patient counseling and treatment, and storage conditions, through standardized supervision questionnaires.

### B. Qualitative methods

#### B.1. Recruitment and sampling methods

A purposeful sample of 40 trained HCPs and 41 patients participated in the qualitative research according to the theoretical saturation criterion when focus groups (FGs) and in-depth interviews (IDI) were performed until a clear pattern appears and subsequent groups do not produce new information [22]. The goal was to understand in-depth perceptions about and experience with the qualitative G6PD test implementation. HCPs to be trained from malaria health posts were drawn from a list provided by the municipality. G6PD normal and deficient patients reported in the database up to the time of analysis but who were not symptomatic at the time of recruitment were also drawn. When the selected individual was not available for both HCPs and patients’ interviews, another from the same area was invited to participate.

#### B.2. Perceptions of HCPs and patients over implementation

IDIs and FGs were conducted pre-training and seven months post-training with HCPs and seven months post-implementation with selected (normal and deficient) patients from all malaria posts. HCPs were evaluated regarding information on professional experience, education, knowledge on the treatment of *P*. *vivax* malaria, PQ use, acceptability of training and use of rapid test in the field, G6PDd reporting, antimalarial side effects, as well as recognizing knowledge gaps and suggestions for improvement of implementation strategies. A semi-structured interview guide with open-ended questions, complementary questions and instructions was developed and previously validated in a small sample of participants, which allowed the interviewer to investigate the subject in more detail. Research areas were refined based on discussions and agreements within the interdisciplinary research team with experience in qualitative research and malaria. To assess user satisfaction over the use of RDT into routine, a sample of patients was selected to participate in IDIs. An interview guide was also prepared with questions about perception of pre-test orientation, test application and post-counseling, perceived utility and satisfaction over the test application. These materials are available as S7 File.

### Data curation, statistical analysis, and qualitative data analysis

Descriptive statistics were used for demographic and training data. Diagnostic performance of the test was calculated comparing the quantitative point-of-care test (cut-off for deficiency: < 4.0 IU/gHb, as per manufacturer’s instructions). Qualitative analysis was done as previously described [23]. Briefly, audio of the interviews and FGs were transcribed and inserted in the MAXQDA 2020 program. The qualitative analysis was carried out through thematic content analysis. Categories were created after transcript reading and analysis and were discussed to reach an agreement between researchers; two researchers developed a codebook and started line-by-line coding.

## Results

### Reliability of interpretation

Implementation of the test started in February 2019, with 110 HCPs trained and assessed irrespective of their previous experience with malaria diagnosis and treatment. Most health workers were able to achieve the minimum passing grade of 90% on the proficiency test after only one training session (n = 86; 78.2%), with 58 (67.4%) scoring 100% and 28 (32.6%) 90% of total points. The remaining HCPs needed at least one additional training round (13 needed a second round, and 11 ≥3 rounds).

Four months post-implementation, 46 HCPs (42%) accepted to participate in a proficiency evaluation using a panel of five tests (two normal, two deficient and one intermediate result). Eleven (23.9%) had used the test during implementation, 31 (67.4%) had not because no malaria case was reported at their unit, and 4 (8.7%) did not disclose this information. Correct answers were seen in 92.4% (85/92) of normal and 92.4% (85/92) deficient results among all HCPs, while 78.3% (36/46) had correct intermediate result answers.

### Test performance in the field

**Fig 3:**
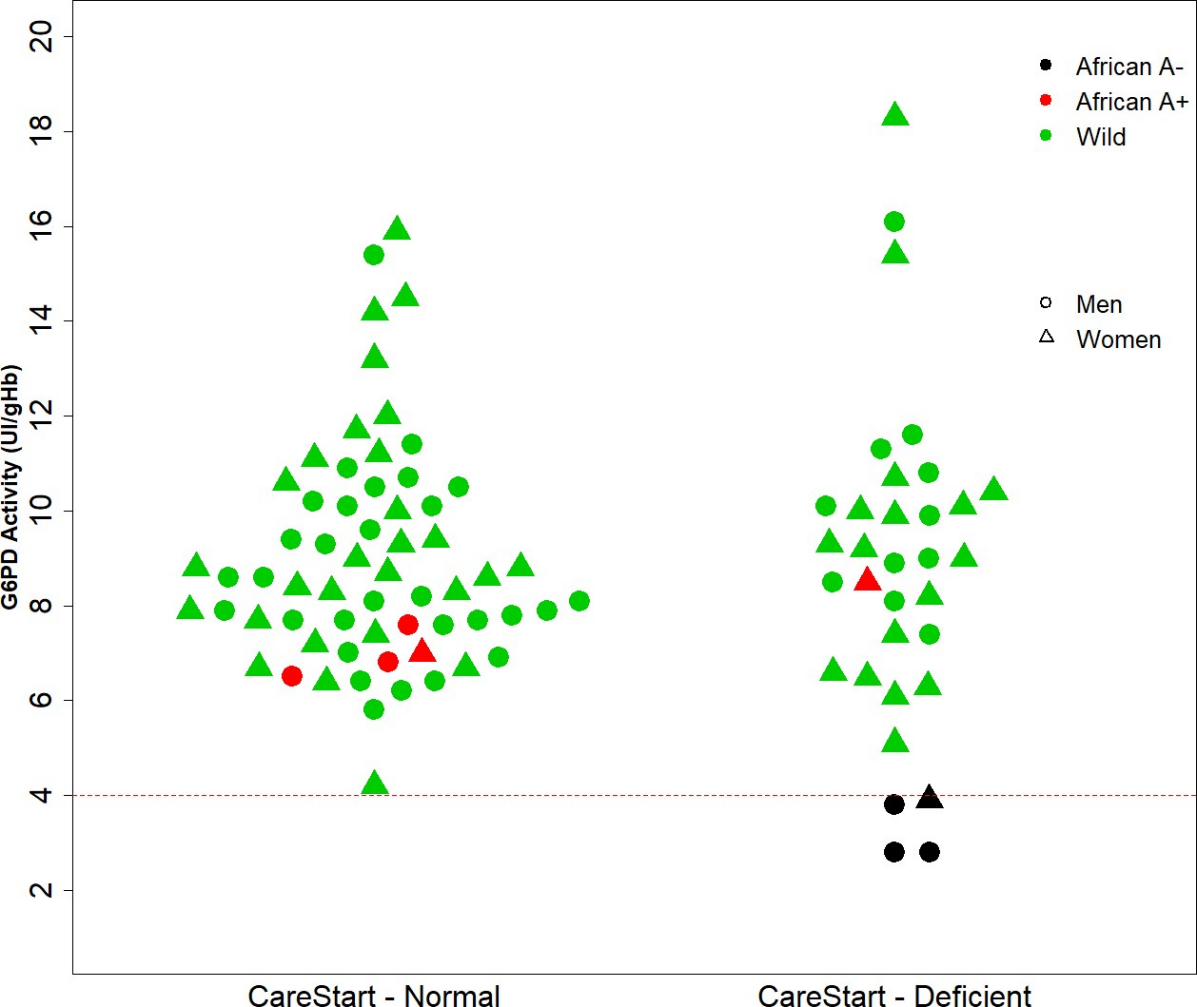
G6PD activity quantitation and genotyping of G6PD RDT normal and deficient samples. G6PD activity measured by point-of-care quantification (Y axis) is shown for CareStart RDT normal and deficient samples (X axis left and right, respectively) in Rio Preto da Eva, Brazil. G6PD African A-, African A+ and wild type variants are indicated by black, red and green colors, respectively. Circles below the red-dashed line (cut-off point 4.0 UI/g Hb) are considered G6PD deficient (<30%) as per quantitative test results.

During implementation (February 2019 to early January 2020), 72 G6PD deficient and 267 G6PD normal cases were detected using the CareStart RDT. The test’s reliability in the field was assessed on a subset of 33 patients (14 males and 19 females) with a deficient result and 62 patients (33 males and 29 females) with normal results from different MTUs. Their enzymatic activity was measured (median time from RDT result: 109 days [IQR 20–175]) and G6PD variants were genotyped (Fig 3). The median activity in the RDT normal and deficient groups were 8.5 UI/gHb (IQR 4.2–15.9) and 9.0 UI/gHb (IQR 2.8–18.3), respectively. From RDT deficient results, only four (three males and one heterozygous female) were true deficiencies (G6PD African A-). Among all samples collected, four were identified as G6PD African A-, five as A+, and no Mediterranean variant was detected in this subset of samples. Using the G6PD intermediate threshold provided by the biosensor’s manufacturer, (30–80%; 4–6 IU/gHb), three patients were detected: one female and one male RDT normal and 1 female RDT deficient (Fig 3).

Diagnostic parameters showed 100% sensitivity (4/4; 95%CI 39.8–100.0) and 68.1% specificity (62/91; 95%CI 57.5–77.5), with a prevalence of 4.2% (95%CI 1.2–10.4). Negative and positive predictive values were 100% (95%CI 94.2–100.0) and 12.1% (95%CI 3.4–28.2), respectively. In this scenario, 29 (87%) false deficient patients out of 33 diagnosed in the RDT missed the chance of radical cure due to low specificity.

### Perceptions of malaria work routine before training and implementation

The fieldwork conducted before Carestart routine implementation by HCPs included 40 IDIs using a semi-structured guide. The themes that emerged from the data were related to the knowledge about clinical signs in patients who had problems (i.e., adverse events) with the medication during vivax malaria treatment and about the diagnostic tools associated with the interviewees’ work routine.

### A. Signs of hemolysis

Before training, most HCPs (60.4%) were unaware of the adverse effects of PQ and G6PDd and were unable to establish a cause-and-effect relationship between PQ adverse reactions and these patients, despite reporting having seen signs of hemolysis such as jaundice and dark urine in patients undergoing treatment (Table 1). One participant described an occasion when he contracted malaria and observed such signs during his own treatment.

**Table 1:** Signs of hemolysis—IDIs with HCPs.

Participant	Response
HCP 38	*“I have seen people that were taken to the hospital vomiting because of the medication and found out that they were allergic to it”*
HCP 14	*“I saw a shocking case last year*! *It was a boy who was very weak*, *he was dark and suddenly went pale*, *I was very worried about him*, *and then I took him to the hospital*. *The urine was already the color of Coca-Cola*, *he was very weak and did not eat well*. *It was his third malaria episode and the doctor left him in isolation thinking it was hepatitis*. *He still stayed three days in the hospital being medicated and when he left*, *he got better”*
HCP 15	*“I was sick during the treatment of malaria*. *My urine went dark the color of Coca-Cola but I never went to the hospital”*

### B. Rapid diagnostic tests

Most professionals (75%) had never seen or used a RDT and were receptive to it. However, some, especially HCPs in rural areas, had already used a RDT for malaria diagnosis and the experience was negative. Many (60.9%) complained about the low sensitivity of the malaria RDT and were resistant to the idea of using it again, even for a different purpose (detection of G6PD levels).

“*My husband had malaria and that’s when I discovered a problem with the rapid test*: *the big problem with the test is that it only shows positive if the parasitemia is high*. *We went to another malaria outpost with a microscopist and it was positive on the same day*, *so I prefer the thick drop because it is more reliable*.*”* (HCP 40).

### C. Monitoring malaria patients

One of the challenges reported by the interviewees was the difficulty in monitoring the treatment of some malaria patients. These patients usually have jobs in poultry or agriculture farms, are frequently nomads and migrate in search of better job opportunities. According to HCPs (31.3%), some patients do not return after treatment for cure confirmation through a new thick blood smear. Although they search for these patients at registered homes, they claim that it would be important for the patient to be sensitized to return so that malaria control is more effective.

“*We have cases of treatment abandonment by people who work on farms*, *because they leave and there is no way for us to monitor them*” (HCP 03).

The poor awareness of hemolysis and G6PDd was considered in the construction of the training educational methodology.

### Perceptions after training and implementation of CareStart in the malaria work routine

Fieldwork included interviews and FGs with 35 health professionals and 41 malaria patients. Theoretical saturation was observed with this sample number.

### A. HCPs perceptions

#### A.1. G6PD test comprehension and acceptability

The main reasons that could hinder test implementation from the HCP perspective were analyzed. According to the interviewed HCPs, most patients accepted to take the G6PD test. However, some patients were afraid to suffer finger puncture twice (once for malaria diagnosis and the second time for G6PD when positive for *P*. *vivax* malaria; Table 2). A FG solution was to do the RDT together with the malaria slide, which upon evaluation by the research team, it was deemed not feasible at the time, but recommended to be analyzed in a broader implementation policy.

**Table 2:** HCPs perceptions on G6PD test comprehension and acceptability.

Topic	Response (participant)
Test implementation barriers	“.* *.* *. *I think it is invasive to fingerprick twice*. *Here*, *there is no collection at the hospital*, *the diagnosis of malaria only works during business hours*. *To improve this it would be better to collect the sample for the test at the same time as the blood for the diagnosis of malaria*.* *.* *.*”* (HCP 02)
HCP perception of test importance	*“It is important to get tested because if the person takes the medication and is allergic*, *they may have complications such as dark urine*, *yellow skin”* (HCP 15)
Patient understanding of the test	*“I think the patient understands the reason for the test because before the patients had a reaction to the medication and they did not know why and now they think that this is why they felt bad when they took the medication”* (HCP 03)
Cultural beliefs	*“*.* *.* *. *Some patients refuse because they always say that*, *for example*, *the vaccine that the government distributes serves to kill the population*, *especially the elderly*.*”* (HCP 24)

A greater understanding of the reasons for including the test in the routine of these professionals was observed. Although they lacked in-depth knowledge about the action and function of the enzyme, these professionals considered G6PDd (low enzymatic levels) an “allergy” to the drug (Table 2).

Respondents also reported how patients were sensitized to the G6PD test. Most of the interviewees (68.3%) believed that patients had difficulties understanding the concept of the test but accepted to do it because there was a trust long established due to years of malaria work in the Amazon (Table 2).

In the analysis it was also observed that within the culture there were many strong popular beliefs mainly among the elderly, such as conspiracy theories that would make it difficult for the patient to adhere to the G6PD test (Table 2). This would be the main barrier in this group, since there is a distrust towards something new.

#### A.2. RDT procedure

As a positive point, respondents reported that the test was easy to perform, but many mentioned challenges in interpreting the test color results.

“*I had difficulty only when an unclear color appeared*, *it was light pink almost white*, *there was a middle ground*, *so we were advised that when I had these doubts*, *it was good to retake the test or seek an opinion from another professional”* (HCP 12).

A consensus among HCPs was that the test should be kept with them and not in the laboratory. However, having only one buffer vial per CareStart kit is something that makes it unfeasible, as it is not possible to divide a test kit between the agents.

### B. Patients perceptions

#### B.1. Reason for testing

**Fig 4:**
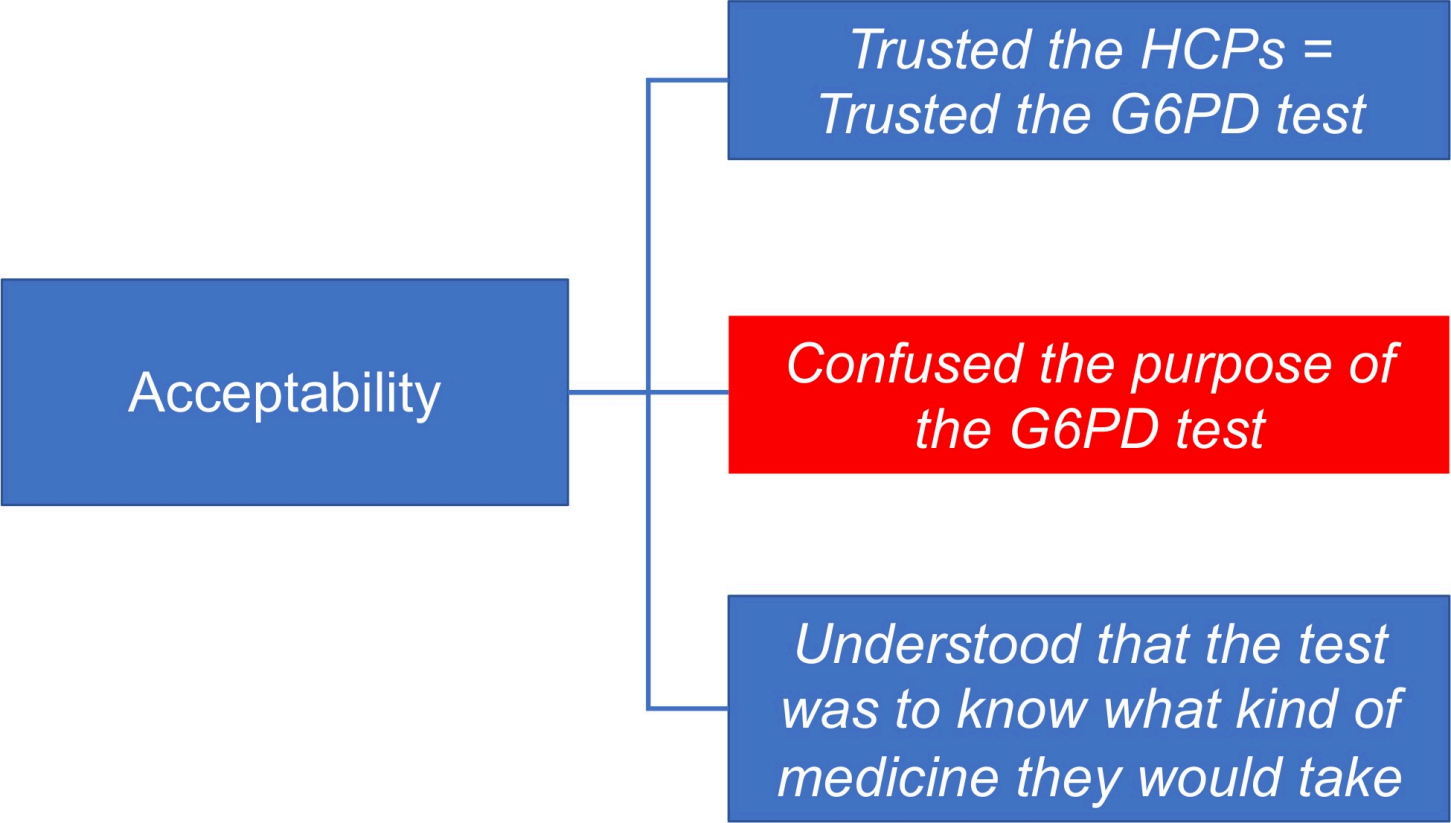
Patient acceptability of the test. Blue and red boxes show reasons for trusting and barriers to the test, respectively.

Most patients (80.5%) understood that the reason for the test was to check resistance to the medication or any type of “allergy”. Other patients reported that the test was to confirm the malaria diagnosis. Eleven patients said that performing the test helped them have greater confidence in the treatment of malaria, since many had a negative experience with the disease (Fig 4).

“… *there are people who are allergic to the medicine*… *and by taking the test you prevent yourself*, *then you already know if you are going to do another type of treatment*…" (Patient 08).

Patients also reported they would prefer the test done together with the sample collection for malaria diagnosis, preventing the HCPs from pricking their finger twice.

“*Two holes in the finger are unpleasant*. *He could have done it all at once*, *it would make it easier*, *especially for a child*.*”* (Patient 05).

#### B.2. Malaria monitoring card

A colored card containing the patient’s data and the RDT result was given to the patients in order to empower them regarding awareness of their health condition (S6 File): a purple card for normal and a gray card for deficient result. Almost all respondents liked having this card. Some patients reported the card made it possible for them to know whether or not they could take the normal medicine in addition to being able to track how many malaria episodes they had contracted.

“*I thought the card was good*, *because they said that when the HCP came again*, *I was supposed to present that card and they already knew what medicine I was taking and already brought the medicine*.*”* (Patient 11).

## Discussion

This study aimed at evaluating implementation of the CareStart G6PD screening into the routine vivax MTUs in the Brazilian Amazon. Despite showing 100% sensitivity, the test led to a high number of false deficient results. Discrepant results over Carestart RDT performance have been reported [9,13,24–26]. Interpretation of the test can be subjective and relies on subtle color changes. A more conservative approach that classifies faint color change as deficient may explain the high number of false deficient results observed in this study. Furthermore, the lack of a control line prevents the identification of an invalid test result, where no color is developed in the reading window even in G6PD normal samples. The difficulty in visualizing the color change can limit acceptability by HCPs in the Amazon.

A recent study from Cambodia showed the ability of trainees with different backgrounds to accurately use CareStart RDT to identify G6PDd in male healthy volunteers [27]. However, in cases where the color change was unclear, sample were not classified as either normal or deficient. In a real-life scenario, where the patient must have a G6PD result in order to receive PQ treatment, the decision will solely rely on HCPs judgement, often with no other confirmatory G6PD test. Thus, more people may be assigned to receive weekly rather than standard PQ, which may decrease adherence. It is not clear whether weekly PQ affect recurrences of *P*. *vivax* compared to shorter regimes [28], a question that requires further studies conducted on larger populations. Due to ethical concerns at the time of analysis, it was decided to withdraw the CareStart RDT from the municipality and replace it with the quantitative Standard G6PD Biosensor (SD Biosensor, Korea), the operational performance of which will be analyzed separately.

Although HCPs have already observed patients undergoing treatment for vivax malaria with clinical signs compatible with PQ hemolysis, most professionals had never heard of G6PDd. However, after training and implementing the test, the use of basic G6PDd concepts was observed in their speeches. They incorporated the knowledge learned and adapted the scientific explanation to pass it onto the population, exchanging some terms and incorporating others, such as allergy and liver test. In our scenario, where PQ is the leading cause of morbidity [4], training was focused only on how to discriminate patients that “can use daily PQ” from others that “cannot use daily PQ”, how the absence of testing for G6PDd can lead to hospitalizations, and how to identify AHA signs and symptoms in order to refer misdiagnosed cases to a specialized health facility. The training did not provide an in-depth G6PD counseling or detailed genetic explanations. There is a lack of knowledge on G6PDd and PQ-associated AHA in the Western Brazilian Amazon population. In fact, many cultural myths regarding malaria transmission and treatment still hamper control and elimination efforts at primary care level [23]. Even at higher levels of care, AHA cases can easily be misdiagnosed. Drug-induced AHA in G6PD deficient patients is complex and requires a multifactor analysis for diagnosis and case management.

A negative aspect reported by the HCPs was the difficulty of performing the test in the field due to the increased workload. In Burkina Faso, researchers pointed out that the increased workload of health professionals was a reported disadvantage in the use of RDTs for malaria diagnosis [29]. However, because this may be related to lack of technical skill, it can be overcome with frequent HCP training and refresher training [30].

Participants reported a need for more information about the purpose of the G6PD test. Informative/educational materials should be developed based on the local context, empowering health professionals to act as multipliers of this knowledge [31]. HCPs were considered by patients as having credibility and knowledge, so they should be considered as key people in the educational process about new tools for diagnosing and treating malaria.

This study had limitations. Quality control for this kind of test is hampered due to a lack of a control line. In order to standardize results and minimize subjectivity with r-G6PD controls, all results (normal, deficient and intermediate) used in proficiency analyses were seen by three experienced readers before their use. External factors may have affected the test performance, especially humidity and temperature. Those, however, are generally not monitored in the field, especially in remote areas of the Brazilian Amazon. Variations in individual hematocrit levels and anemia are known to affect the reliability of G6PD enzyme activity measurements [32–34], and could have affected test results here, but they are not routinely measured. Field measurements by RDT and reference biosensor testing were not done at the same time, which may have affected the calculated RDT performance. End user errors may also have played a role and can be addressed through additional training and quality control. Directly supervised therapy (DOT), although recommended for weekly PQ, was not always possible in this study, as it relies on availability of personnel to carry out visits and may be greatly impaired in locations with a higher case load. Since this was an exploratory analysis, caution is needed when interpreting diagnostic performance data due to the small sample size. Regarding the results from the interviews and focus groups, some of the findings might not be generalizable to other settings.

In conclusion, HCPs can carry out G6PD screening in a real scenario using the CareStart platform with minimal training. Although the RDT showed high sensitivity, the poor specificity translated into suboptimal treatment in a large proportion of the tested cases. The implementation of quantitative POC diagnostic tools may be considered in order to increase specificity and to provide proper radical cure in heterozygous females, who remain at risk of hemolysis [35]. In addition, return visits at day 5 post-treatment are advised in order to monitor signs/symptoms of AHA in the Brazilian Amazon owing to current test limitations.

## Supporting information

S1 File Training slides. A set of slides was produced to cover the minimal information necessary to conduct the test in the field.
(PDF)

S2 File Test procedure for training purposes. A locally recorded video was used for trainings sessions and to be distributed for HCPs during the implementation period. Due to the size of the file, please access it here: https://drive.google.com/file/d/1mm3FyP5S1soZzms1lnYjSJ3q2C_aLWl-/view?usp=sharing.
(DOCX)

S3 File Test procedure folder. Folders were provided for every professional during the training sessions **(**Size when folded: 21.0 x 29.7cm).
(PDF)

S4 File Test procedure wall poster. Posters were provided for each unit for quick guidance **(**Size: 29.7 x 42.0cm).
(PDF)

S5 File Malaria reporting form. Forms were adapted from the national SIVEP-malaria reporting forms in order to add data on G6PD results and pharmacovigilance of hemolysis.
(PDF)

S6 File G6PD result cards for patients. A purple card was given to normal and a gray card was given to deficient patients, where the result was recorded and used in future malaria episodes (Size: 8.0x20.0cm).
(PDF)

S7 File Qualitative interview guide. (PDF)

S8 File Datasets. (ZIP)
