# Supplementary material for: Real-life implementation of a G6PD deficiency screening qualitative test into routine vivax malaria diagnostic units in the Brazilian Amazon (SAFEPRIM study)
Source: PLoS Negl Trop Dis. 2021 May 18;15(5):e0009415. doi: 10.1371/journal.pntd.0009415 (PMC8162658; doi:10.1371/journal.pntd.0009415)
Supplement: S1 File — A set of slides was produced to cover the minimal information necessary to conduct the test in the field. (PDF) [file pntd.0009415.s001.pdf]

TRAINING TO PERFORM THE RAPID DIAGNOSTIC TEST (RDT) FOR THE SAFE USE OF PRIMAQUINE

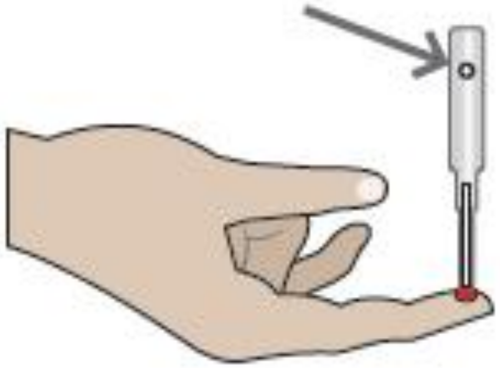

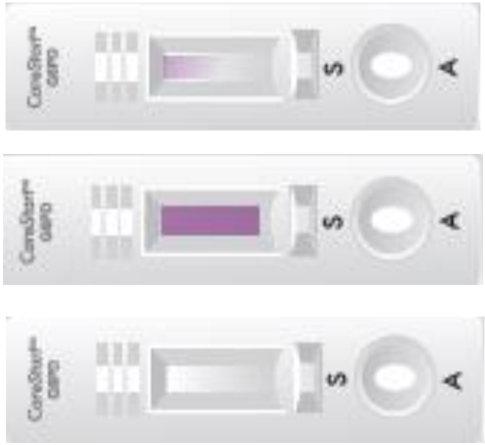

Rio Preto da Eva , Amazonas, Brazil
2018

Event Organizers:

Production:

FMT-HVD
Fundação de Medicina Tropical - Doutor Heitor Vieira Dourado

Institute ELIMINA

ILMD
INSTITUTO LEÔNIDAS & MARIA DEANE
Fiocruz Amazônia

Support:

Fundação de Vigilância em Saúde do Estado do Amazonas
Laboratório Central de Saúde Pública – LACEN-AM

Municipal Health Secretariat
- Rio Preto da Eva

SUSAM

Programme

1. Introduction
2. How to use the rapid test:
   - Performing a finger prick to collect the blood sample - practice
   - Performing the rapid test for safe use of Primaquine - practice
   - interpretation of results - practice
3. Filling in the SIVEP form
4. Patient counseling
5. Proficiency test

1. Introduction – Healthcare Provider

- **Decree No. 44/GM, of January 3rd, 2002** establishes the attributions of Community Health Worker (CHW,) in the prevention and control of malaria and dengue;

- **Law 11.350, of October 5th, 2006** defines the malaria control worker (MCW) as the professional who develops activities for the surveillance, prevention and control of diseases and health promotion in accordance with SUS guidelines and under the supervision of the manager of each federated entity

- **Both work with the community, the neighborhood, the city or the rural region to facilitate the population's access to health and prevent diseases.**

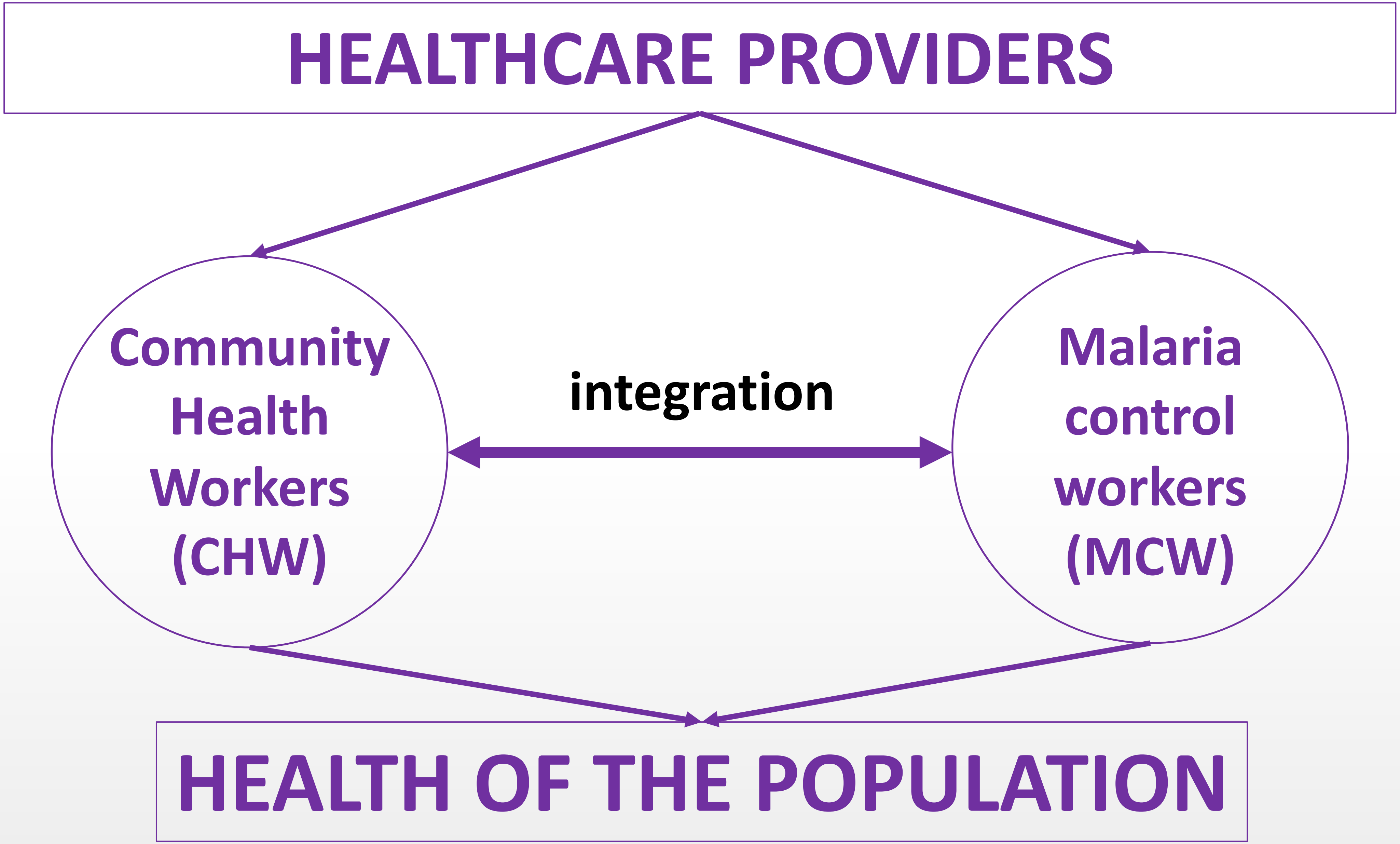
HEALTHCARE PROVIDERS
Community Health Workers (CHW)
integration
Malaria control workers (MCW)
HEALTH OF THE POPULATION

1. Introduction - *P. vivax* Malaria

Malaria caused by *Plasmodium vivax* is the most prevalent in Brazil and, in 2016, accounted for 90% of the cases reported in the Amazon region.

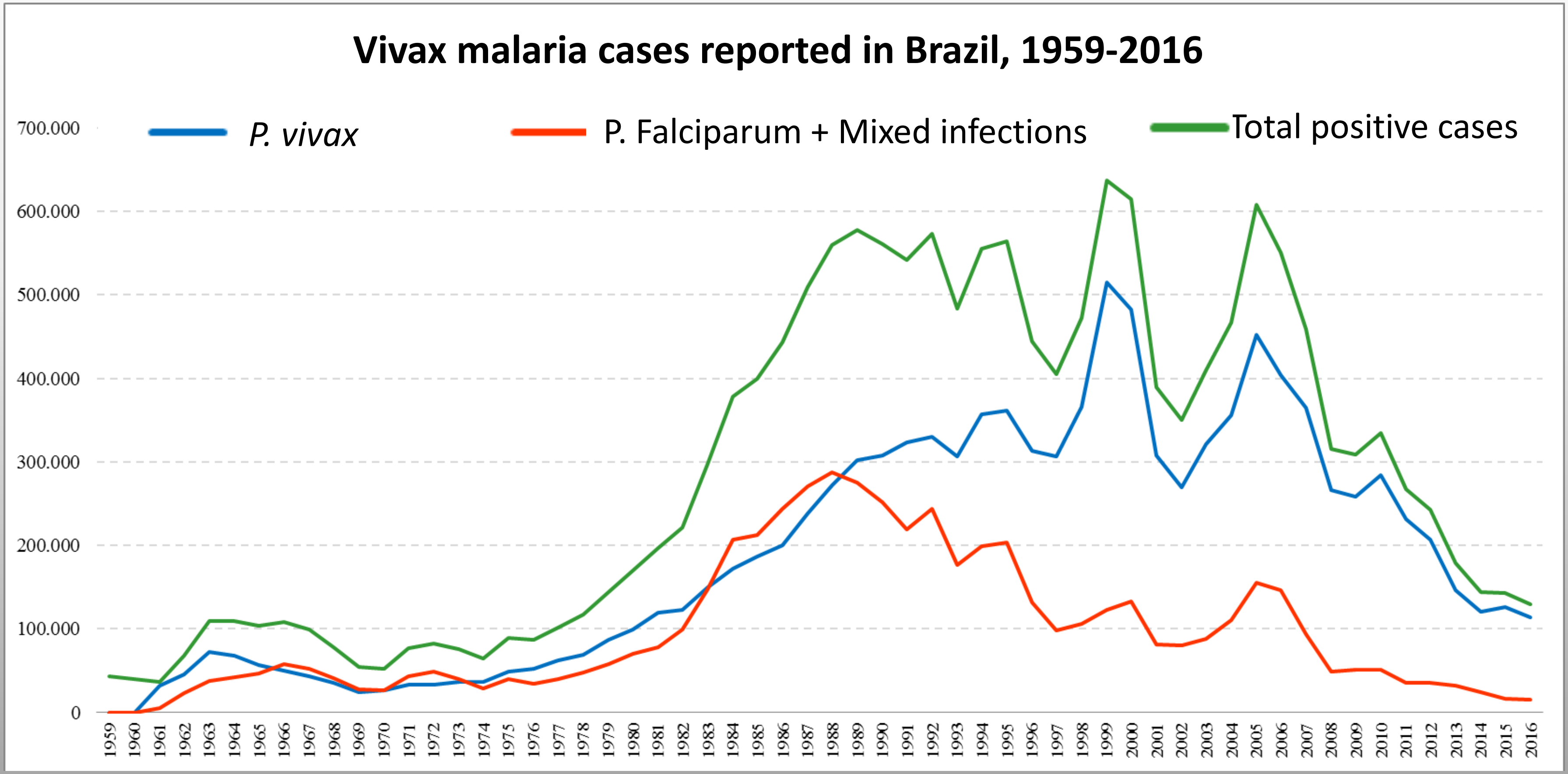

1. Introduction - *P. vivax* Malaria

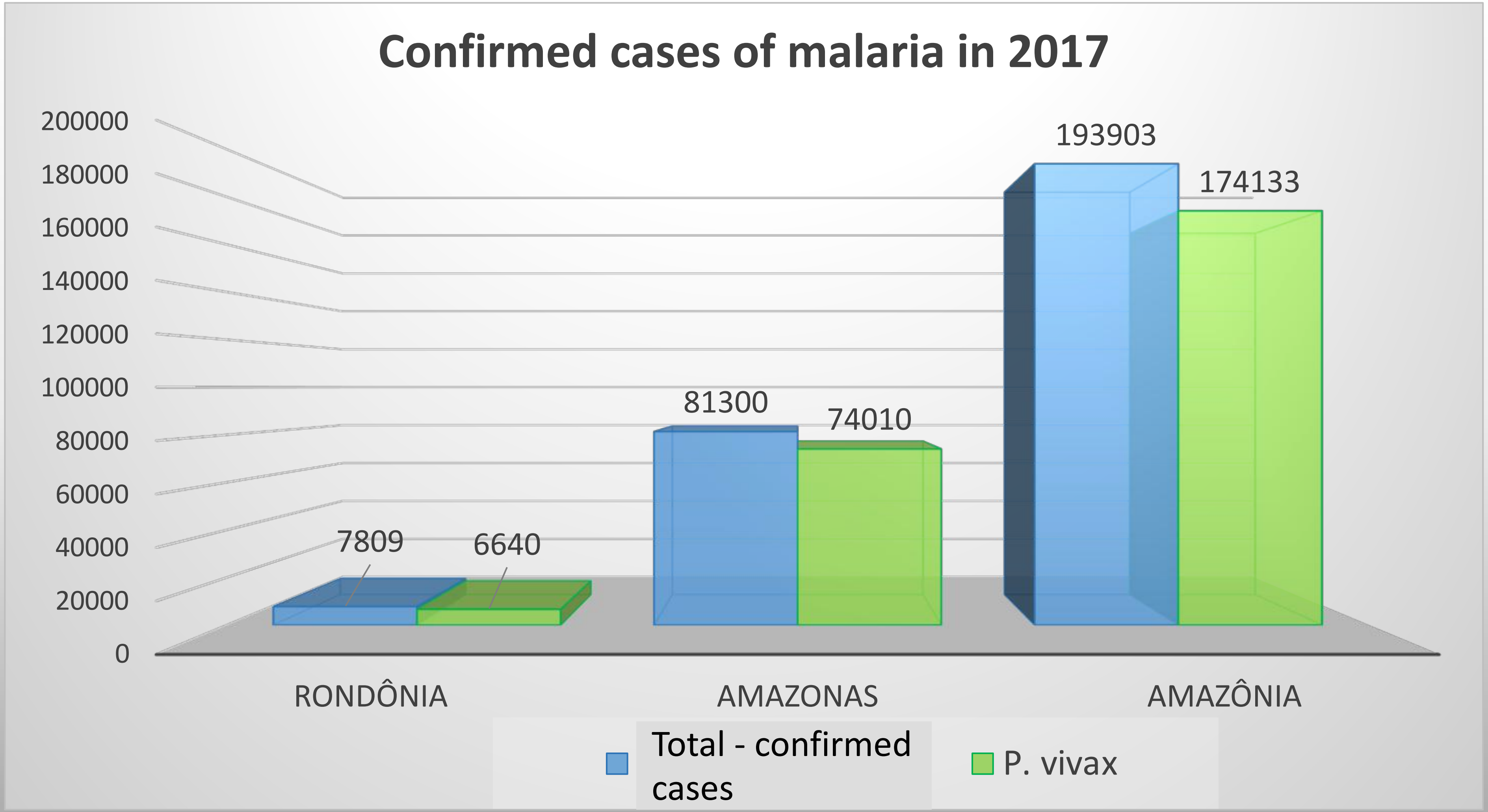

1. Introduction - *P. vivax Malaria*

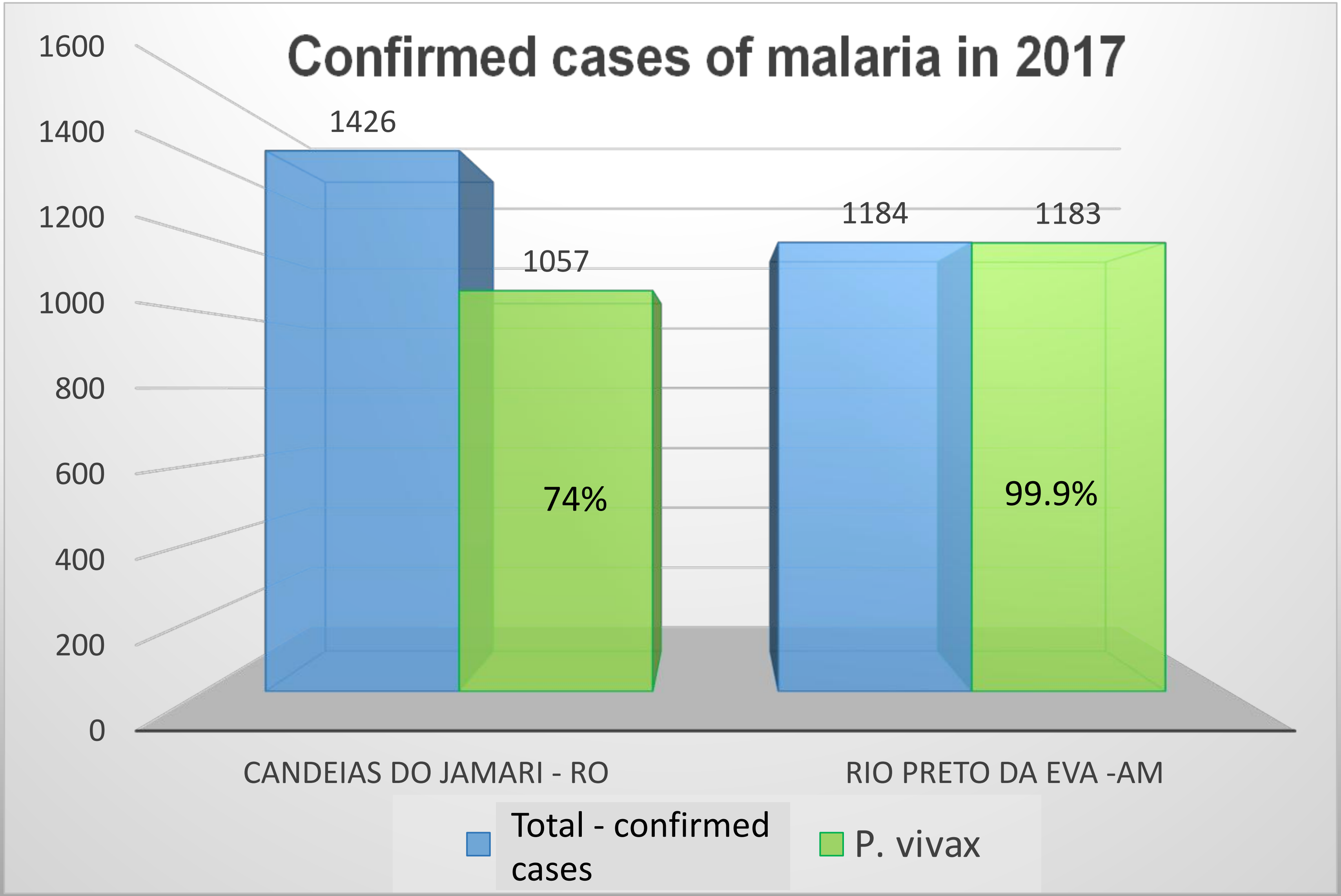

1. Introduction – biological cycle of vivax malaria

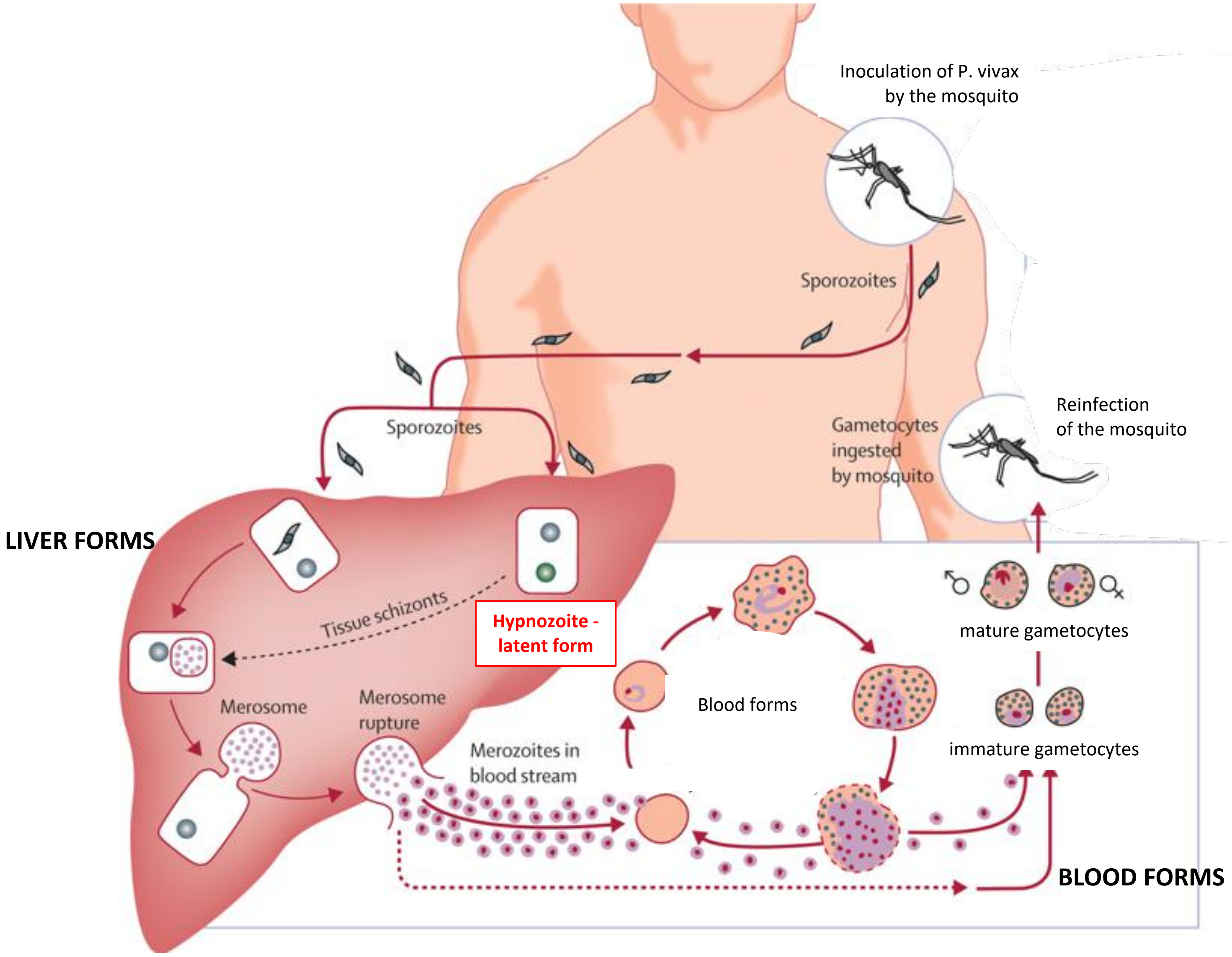

Adapted from Muller et al, ***Lancet***. 2009.

1. Introduction - *P. vivax* Malaria

Plasmodium vivax has a peculiar characteristic in its biological cycle, which is the development of **latent liver forms** called **HYPNOZOITES**, responsible for relapses months or years after an episode of *vivax* malaria.

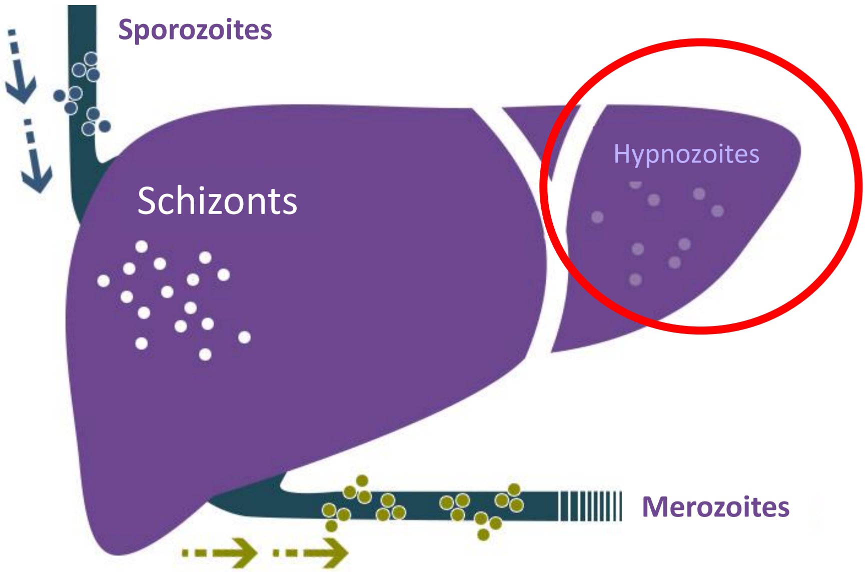

This brings difficulties to the CONTROL AND ELIMINATION of this parasitic species, since a portion of individuals becomes CHRONIC CARRIERS OF *P. VIVAX*, contributing to the maintenance of the transmission cycle.

1. Introduction - Primaquine

- Drug capable of **radical cure** of forms of malaria in which parasites have a dormant stage in the liver, such as *P. vivax*.
- It destroys **gametocytes, and is the most effective antimalarial agent for the prevention of disease transmission** by *Plasmodium* species.

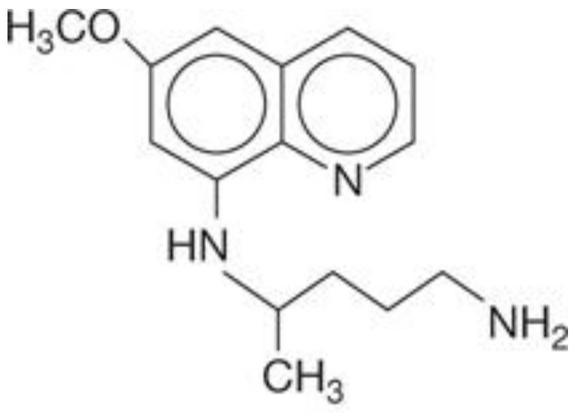

PRIMAQUINE

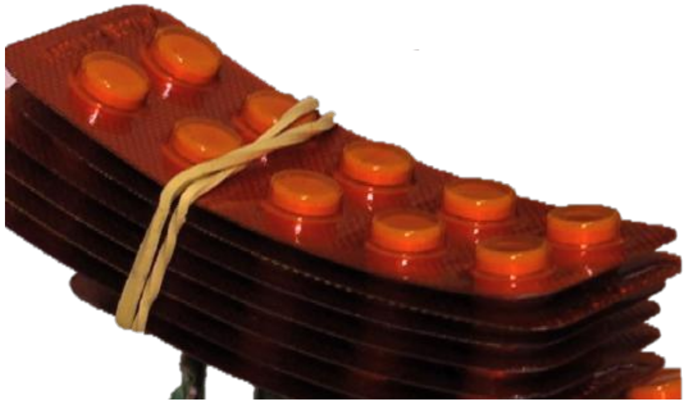

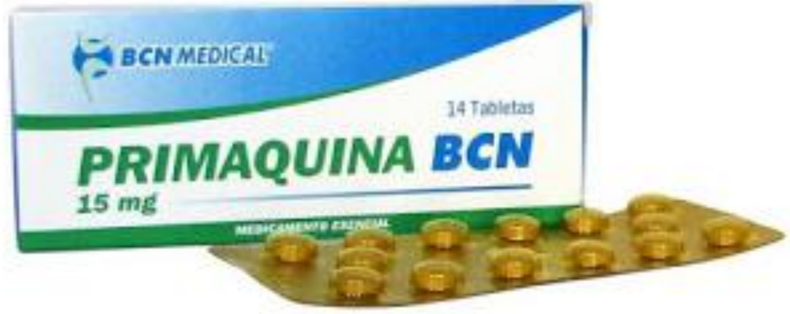

PRIMAQUINE

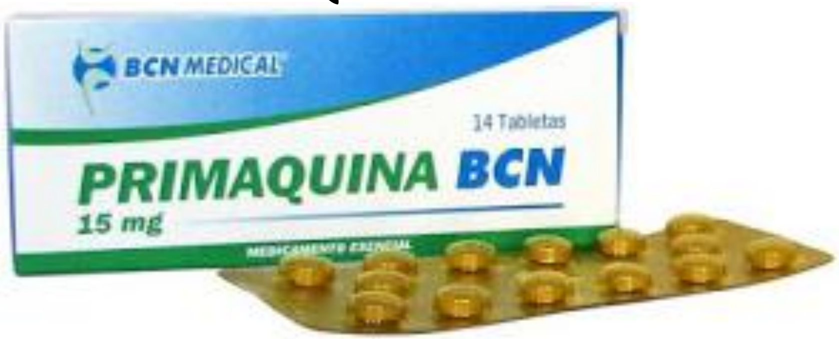

Destroys parasites in the liver

=

No relapse of disease

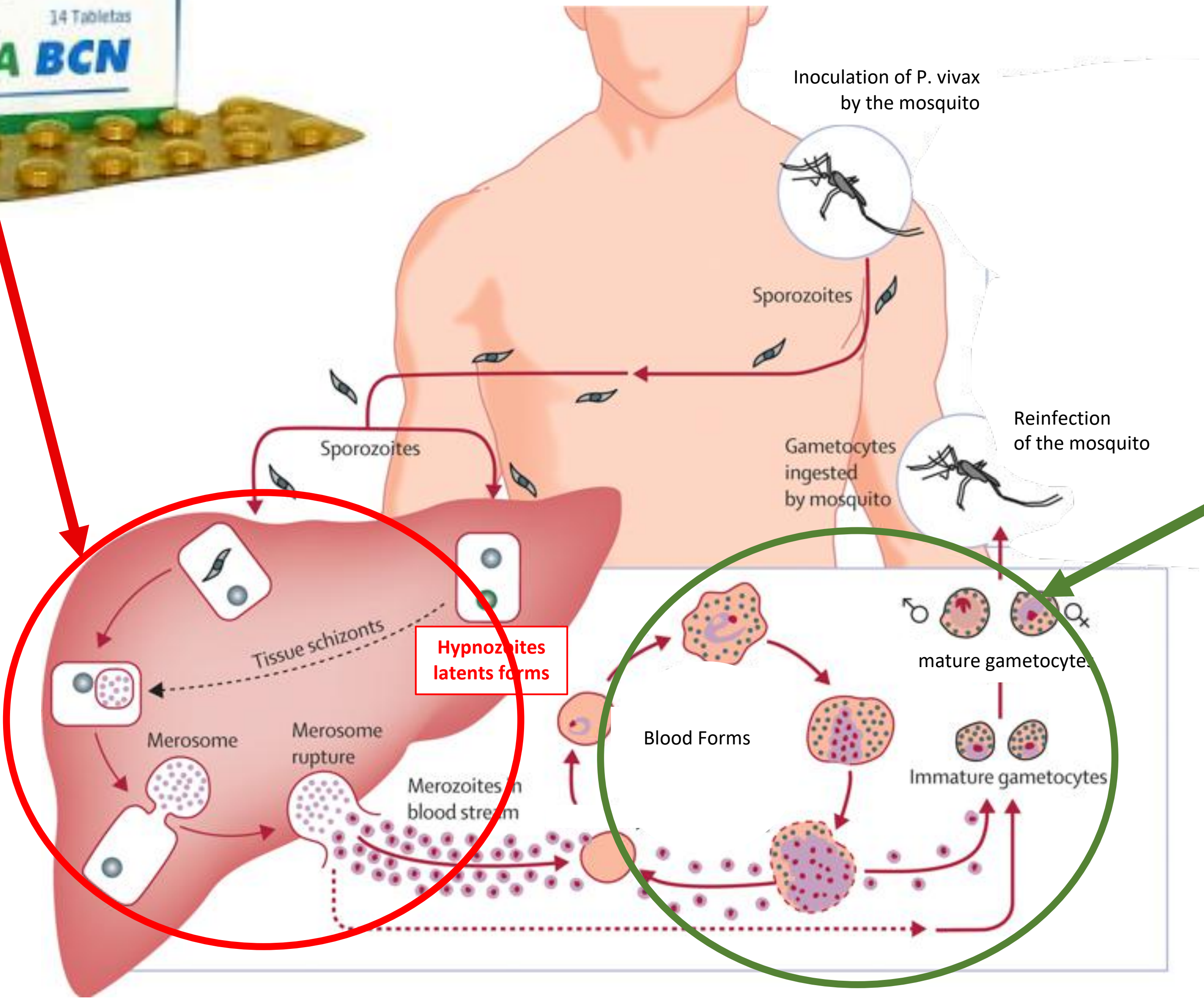

CHLOROQUINE

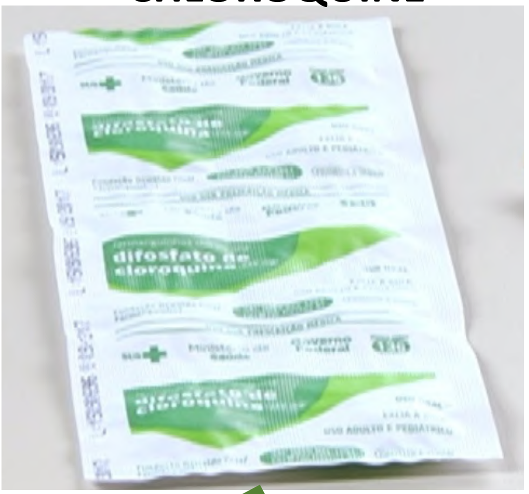

Destroys parasites in the blood

=

No acute symptoms of the disease

Adapted from Muller et al, ***Lancet***. 2009.

1. Introduction - Primaquine

In Brazil, the treatment used for the radical cure of vivax malaria is 0.5 mg/kg/day of primaquine for 7 days (short regimen).

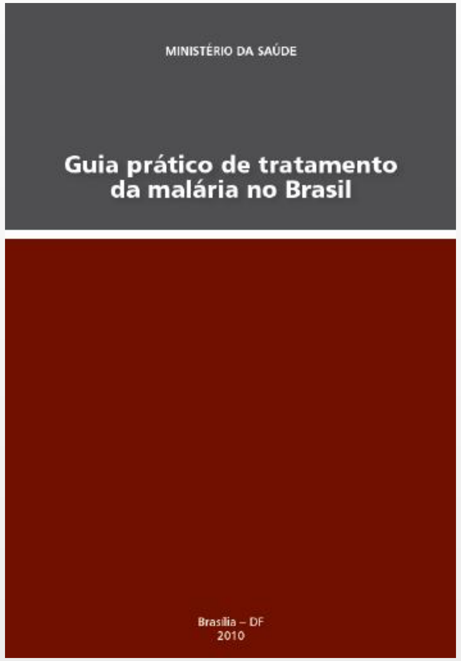

Practical guide for treating malaria in Brazil, 2010.

Age/Weight	Number of tablets of each medicine per day						
	Day 1		Day 2		Day 3		Day 4 to 7
	Chloroquine	Pediatric Primaquine	Chloroquine	Pediatric Primaquine	Chloroquine	Pediatric Primaquine	Pediatric Primaquine
6-11 months 5-9 kg	1/2	1	1/4	1	1/4	1	1/2
1-3 years 10-14 kg	1	2	1/2	1	1/2	1	1
4-8 years 15-24 kg	1	2	1	2	1	2	2
Age/Weight	Chloroquine	Primaquine for ADULTS	Chloroquine	Primaquine for ADULTS	Chloroquine	Primaquine for ADULTS	Primaquine for ADULTS
9-11 years 25-34 kg	2	1	2	1	2	1	1
12-14 years 35-49 kg	3	2	2	2	2	2	1
≥15years ≥ 50 kg	4	2	3	2	3	2	2

PRIMAQUINE

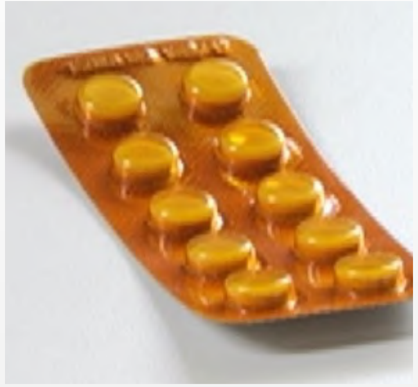

CHLOROQUINE

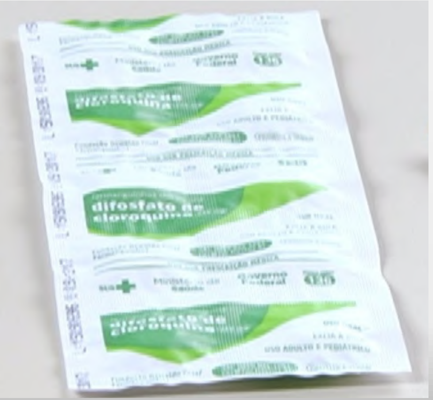

**** Pregnant women and children under 6 months old CANNOT take primaquine**

1. Introduction – Adverse effects of Primaquine

- Some people may experience adverse effects when using primaquine.
  - For example: Acute haemolytic anemia.

- According to the World Health Organization (2018), it is necessary to perform the rapid test for the safe use of Primaquine before treating *P. vivax* malaria.

EVERY patient diagnosed with ***P. vivax* malaria** will be screened with the rapid test for the safe use of PQ to see whether they **CAN** or **CANNOT** take PQ on a daily basis.

1. Introduction – Adverse effects of Primaquine

- Most common: Acute haemolytic anemia.

- **How to identify Acute Haemolytic Anemia:**

- **Signs and symptoms presented by the patient**:
    - ✓ Dark-colored urine (Color of Coca-cola)
    - ✓ Yellow eyes or skin

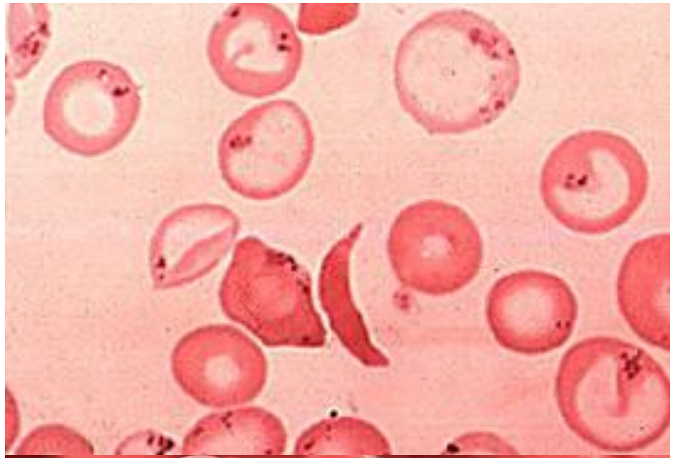

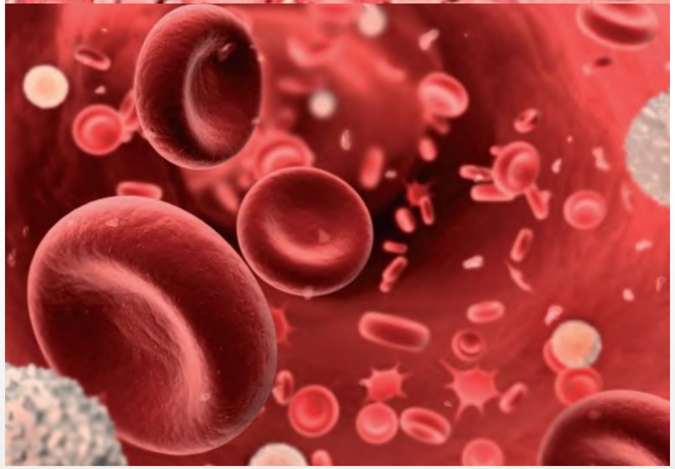

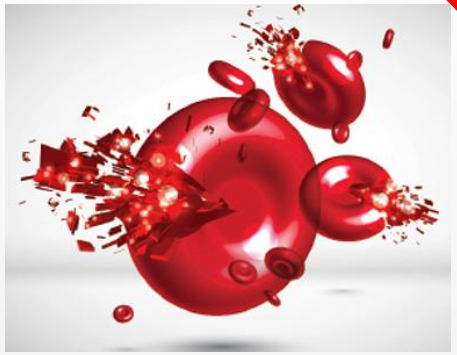

Hemolysis

YELLOW EYES/SKIN

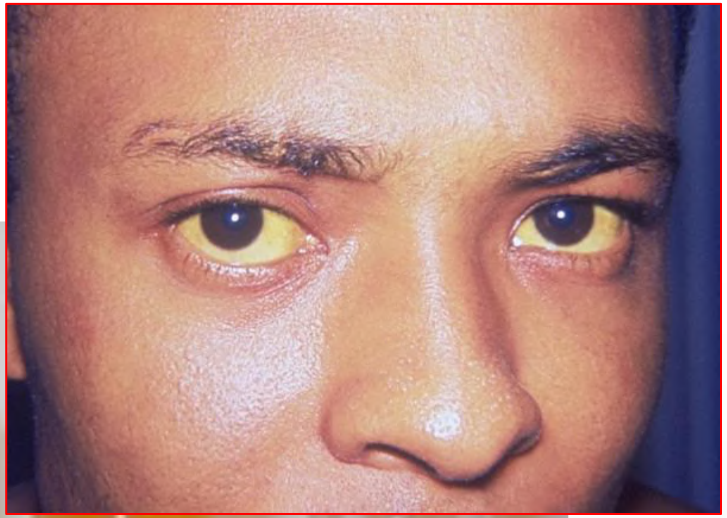

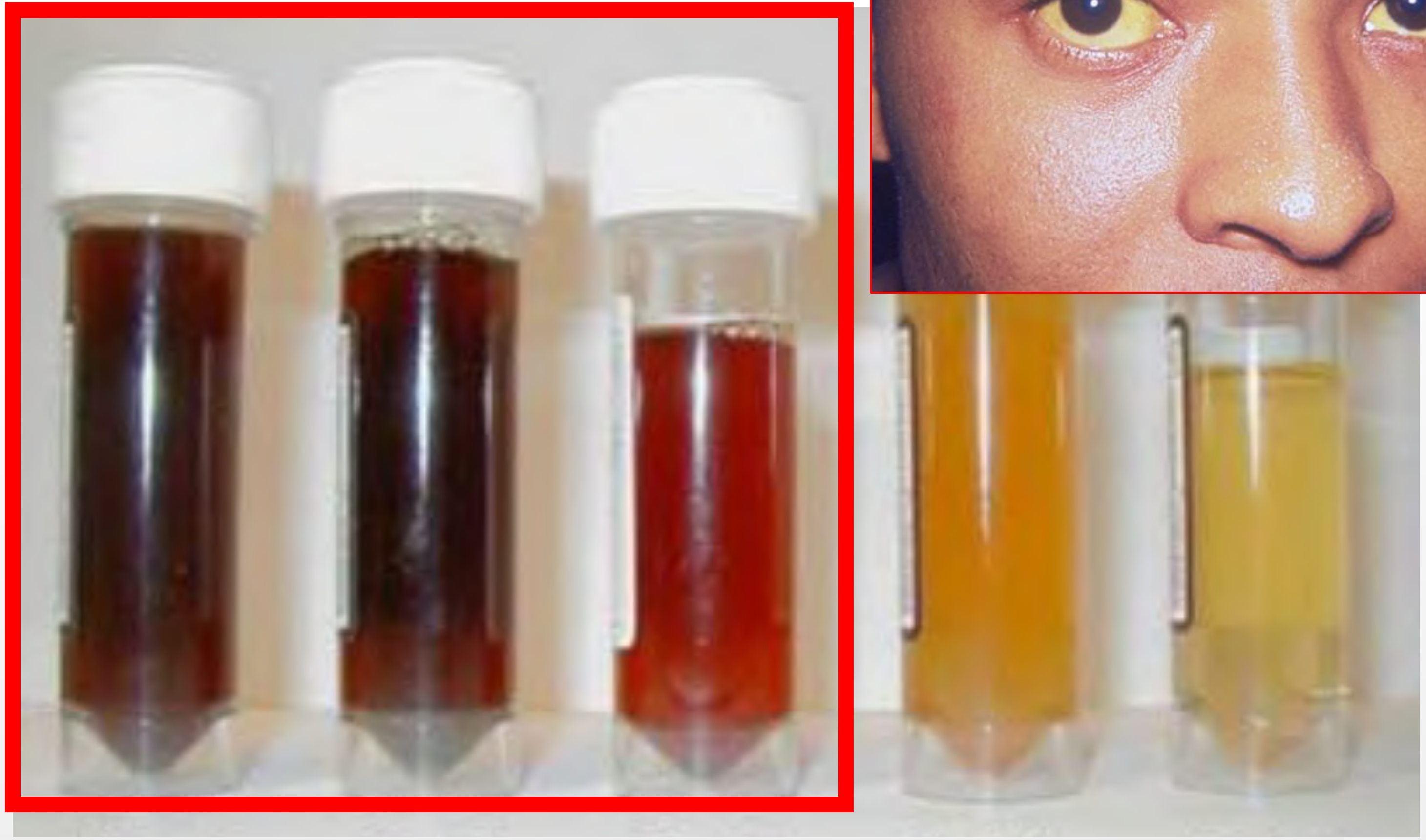

DARK-COLORED URINE

1. Introduction – What are rapid tests?

- Rapid tests are quick to perform. The result is easy to read and interpret, **without the need for specialized laboratory structure**. The result is read with the naked eye.
- For the **rapid test for the safe use of primaquine**, the patient's blood is used in the test device with the addition of a reagent. After 10 minutes, the test result is obtained.

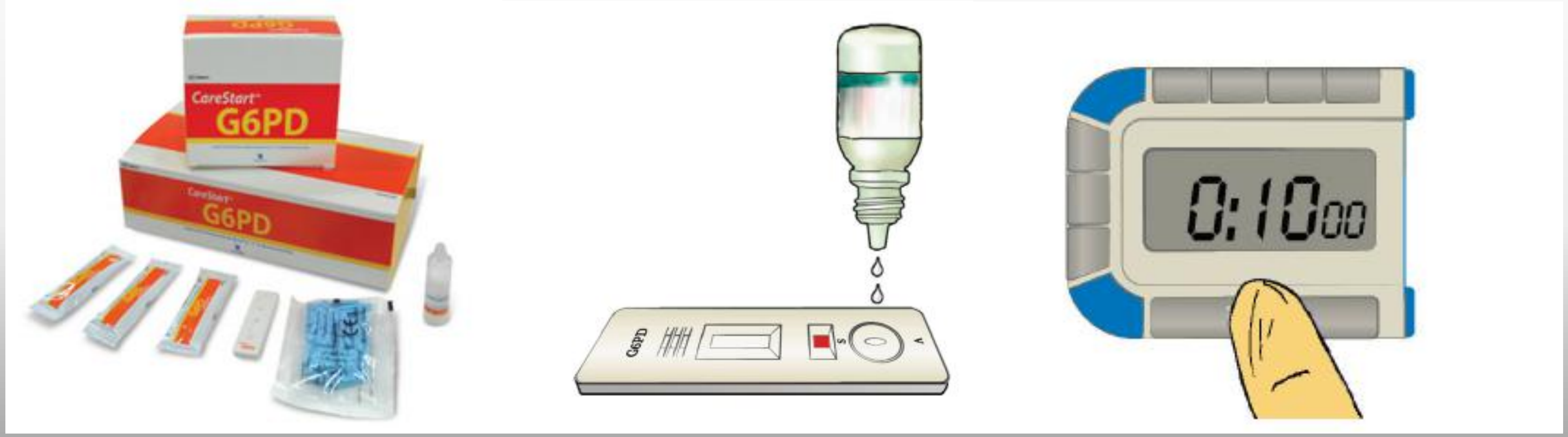

1. Introduction – What are rapid tests?

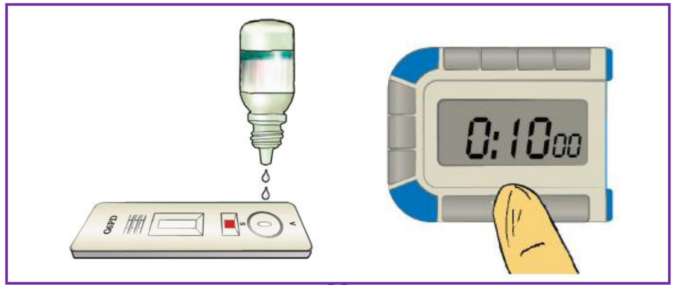

CAN TAKE PRIMAQUINE ON A DAILY BASIS

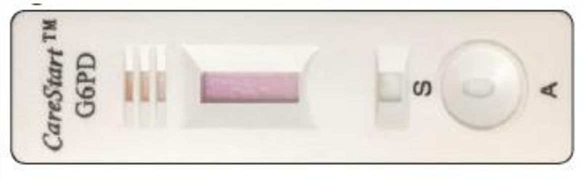

Result is purple

CANNOT TAKE PRIMAQUINE ON A DAILY BASIS

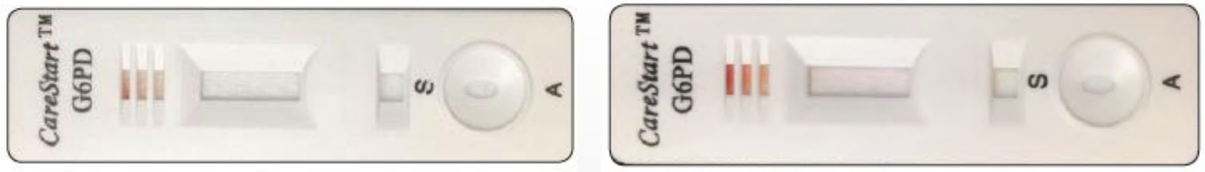

No color change or difficulty seeing color

1. Introduction
# – Why is rapid testing for safe use of primaquine important?

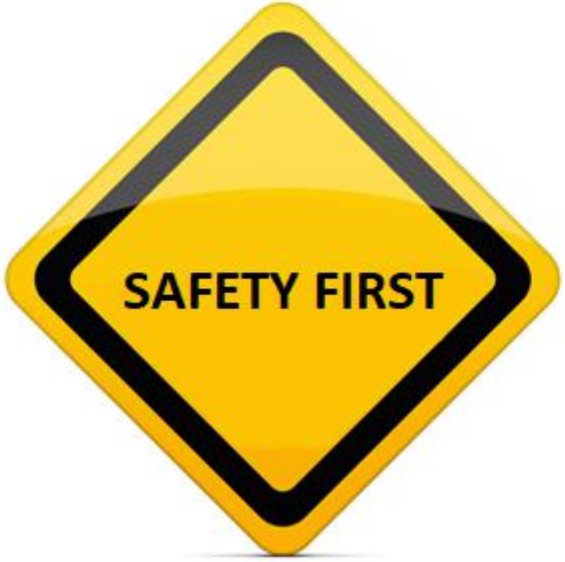

SAFE use of primaquine for **radical cure** of ***P. vivax*** **malaria**

PATIENT SAFETY !!!

1. Introduction – How to store the test

TEST RESULT = ENZYMATIC REACTION MAY BE AFFECTED BY **TEMPERATURE AND HUMIDITY**

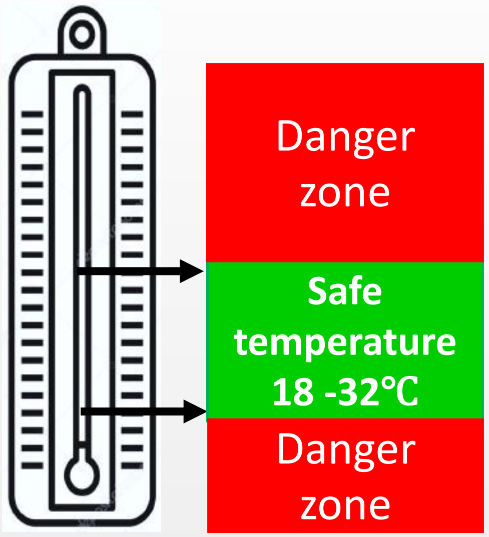

Rapid Test Storage Guidelines
✓ Respect the storage instructions for the tests. the manufacturer's recommended temperature is between **18 °C and 32 °C**.
✓ Do not place in *freezer*.
✓ Store in an organized manner, making tests with shorter shelf life the most accessible.
✓ Control the stock of rapid tests carefully.

1. Introduction – How to store the test

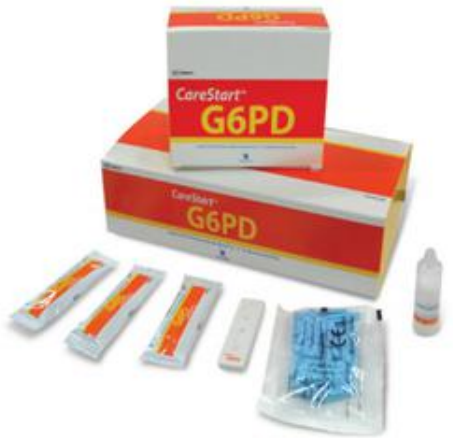

Guidelines for Storage of Rapid Tests
✓ Allocate appropriate storage location.
✓ Storage should be on racks or shelves, **away from the wall and floor in order to avoid damage by water, moisture and contaminants**.
✓ Store in **air-conditioned environments.** When there is no air conditioning or refrigeration, ensure a **natural ventilation system** in the environment, such as windows and vents.
✓ Protect from **the sun, excessive heat, rodents and insects**.

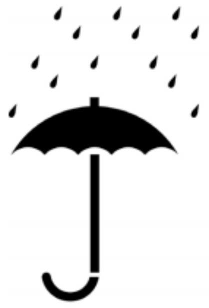

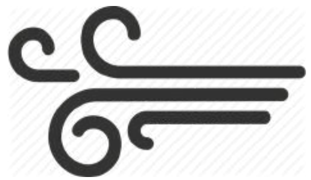

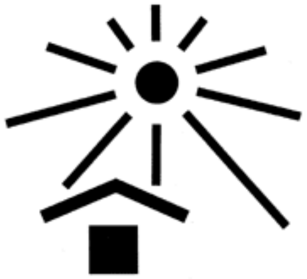

1. Introduction – How to store the test:

Stock management

Primaquine RDT Requisition Form

Person responsible for the request: ______________________

Date of last request: ___/___/___

Date of request: ___/___/___

Clinic: ______________________

Request is for how many days?________

	A	B	C	D	[illegible]	F	[illegible]	[illegible]		J	[illegible]
Medication/Supplies	Previous balance	Usage	Lost/Expired	Returned	Current balance	Mininmum stock	Usage Minimum	Ordered	Delivered	Batch number	Final balance

Date ___/___/___

____
Delivered by

____
Received by

2. How to use the rapid test
# - Preparation before using the rapid test

2.1. Separate the necessary materials and supplies to perform the rapid test:

- ✓ **Rapid test kit**
  - ✓ Alcohol wipe
  - ✓ Lancet
  - ✓ Reagent
  - ✓ Pipette
  - ✓ Test device

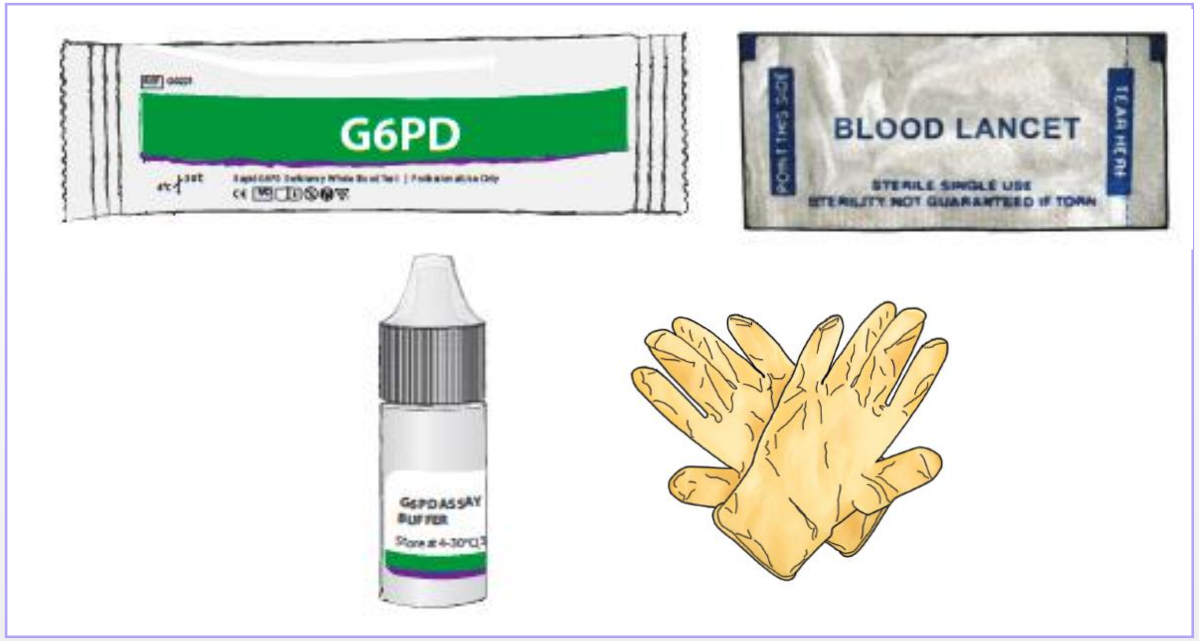

- ✓ One pair of NEW disposable gloves

2. How to use the rapid test
# - Preparation before using the rapid test

2.1. Separate the necessary materials and supplies to perform the rapid test:

In addition to the kit's components, the following materials are required:

- ✓ Sharps disposal container;
- ✓ Common waste disposal container;
- ✓ Pen or pencil
- ✓ Stopwatch

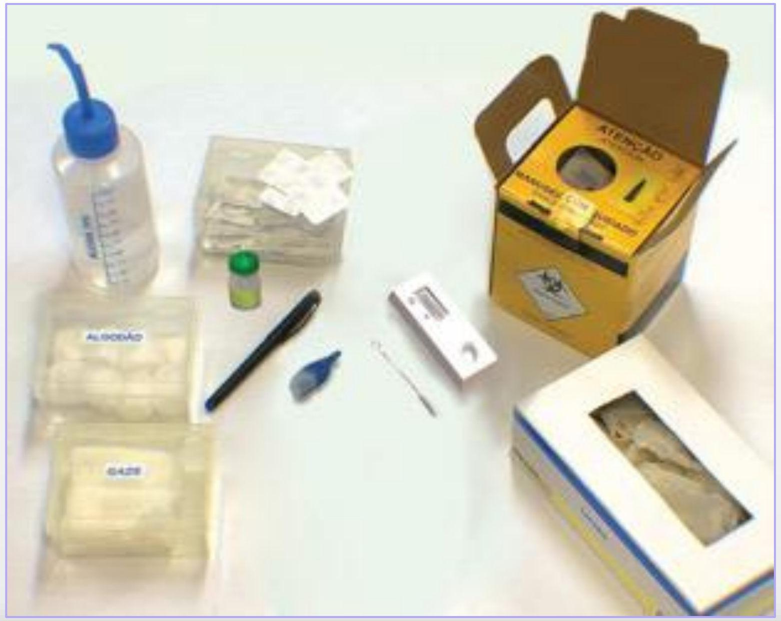

2. How to use the rapid test
# - Preparation before using the rapid test

2.2. Check the test's expiration date:

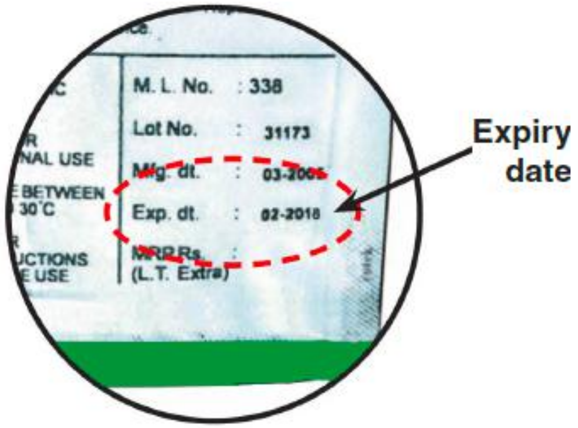

2.3. Professional preparation:

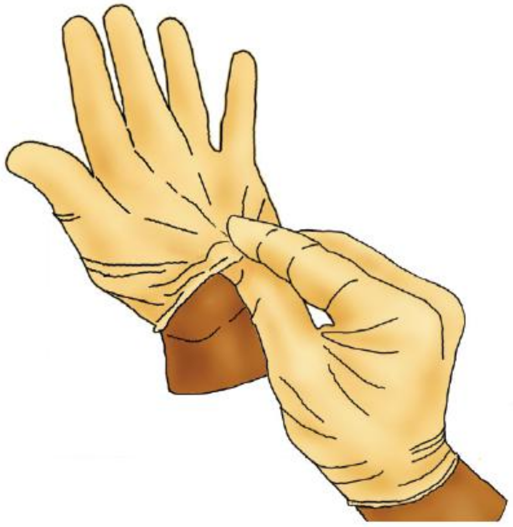

✓Wash your hands using soap and water, 70% alcohol or other antiseptic and put on gloves.

2. How to use the rapid test
# - Preparation before using the rapid test

2.4. Open the test package and remove the contents:

✓Test device

✓Silica gel sachet (keeps the test dry)

✓Pipette

Silica gel

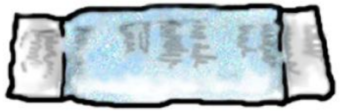

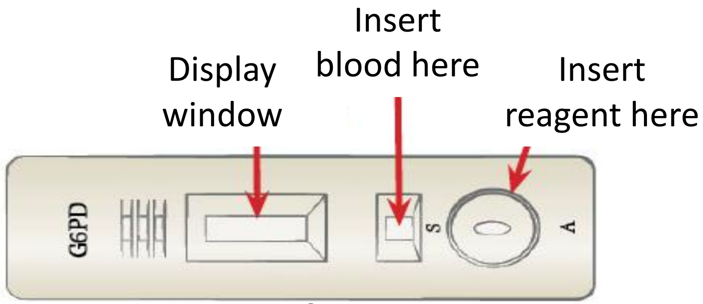

Test device

Pipette

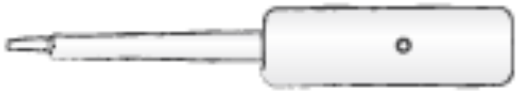

2.5. Write the patient's name on the test device:

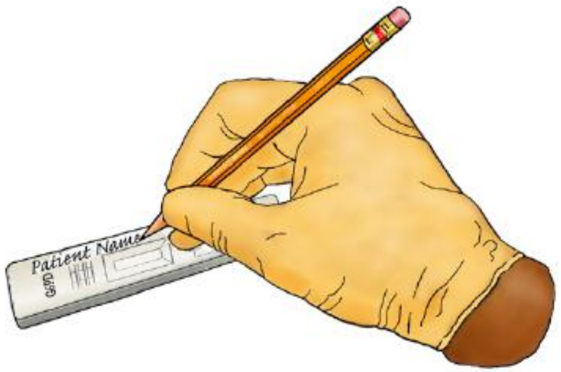

Rapid test device for safe use of primaquine

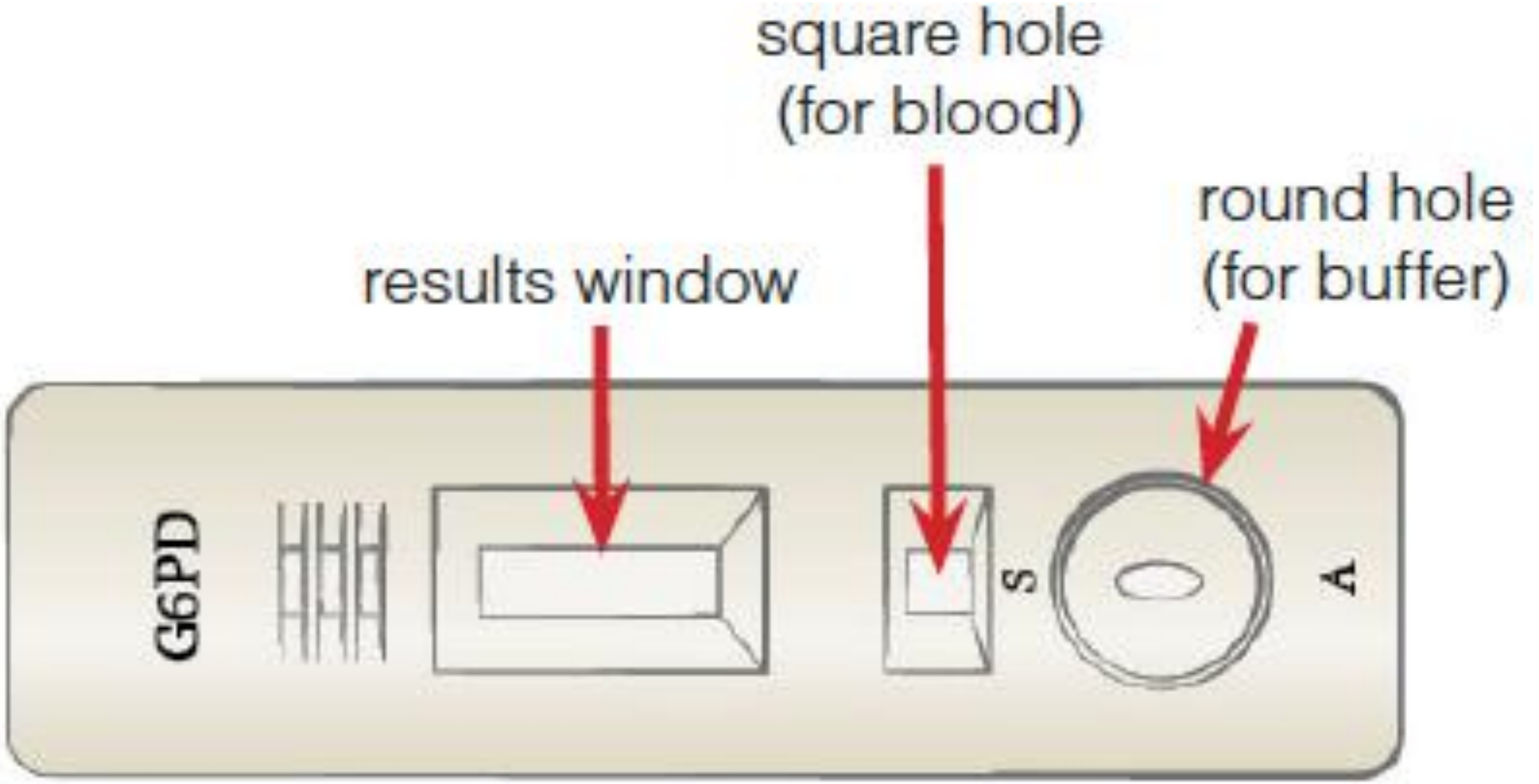

2. How to use the rapid test
# - Preparation for performing the rapid test

2.6. Prepare for the finger prick:

✓Observe the patient's fingers and choose the finger tip of the fourth finger

✓Perform antisepsis at the finger collection site with the alcohol wipe

✓Let the alcohol dry naturally

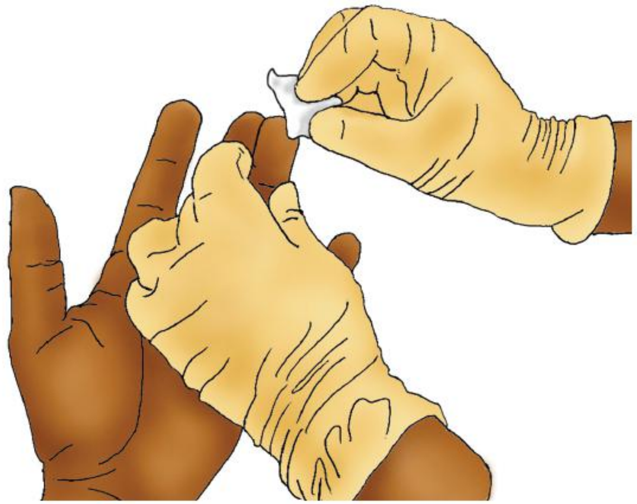

2. How to use the rapid test
# - Sample collection by finger pricking

2.7. Perform the finger prick

- ✓ Open the lancet
- ✓ Press the lancet firmly against the finger and pierce the skin on the side of the finger
- ✓ Discard the lancet **immediately** in the sharps disposal box
- ✓ **Wipe the first drop of blood**
- ✓ **Collect the second drop of blood**

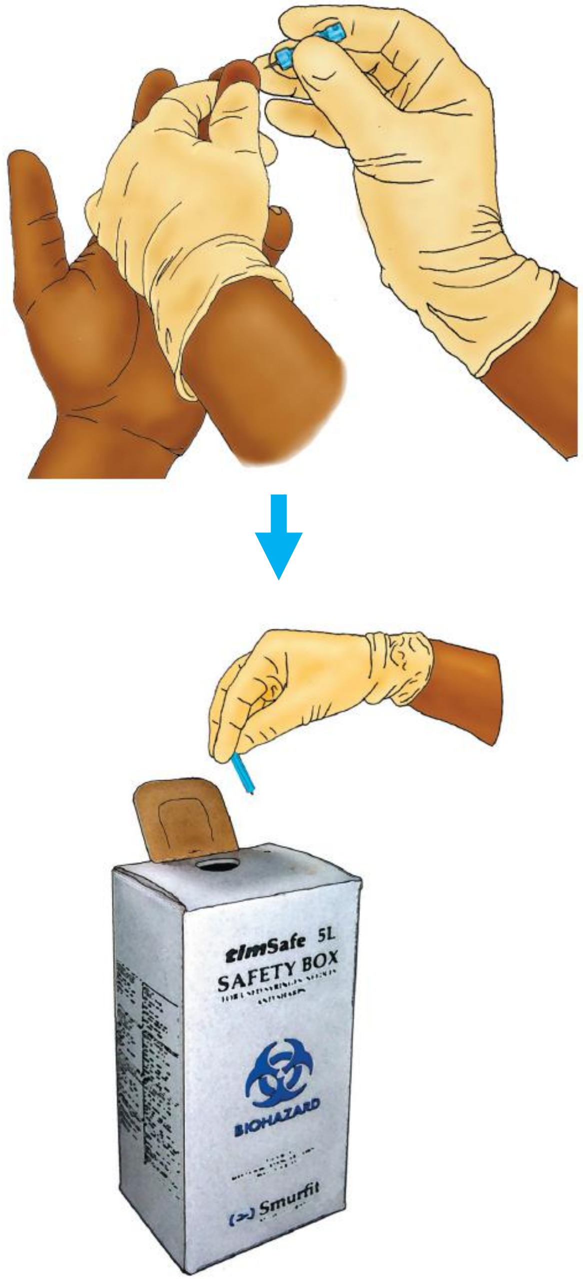

2. How to use the rapid test
# - Sample collection by finger pricking

2.8. Collect blood with the pipette

- ✓Squeeze the patient's finger near the puncture site to form a drop of blood.
- ✓Collect 2 µL of blood (up to the mark) with the individual pipette.

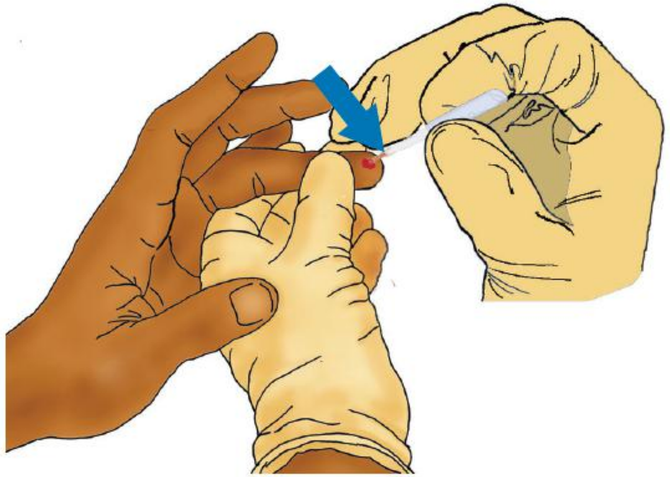

Pipette:

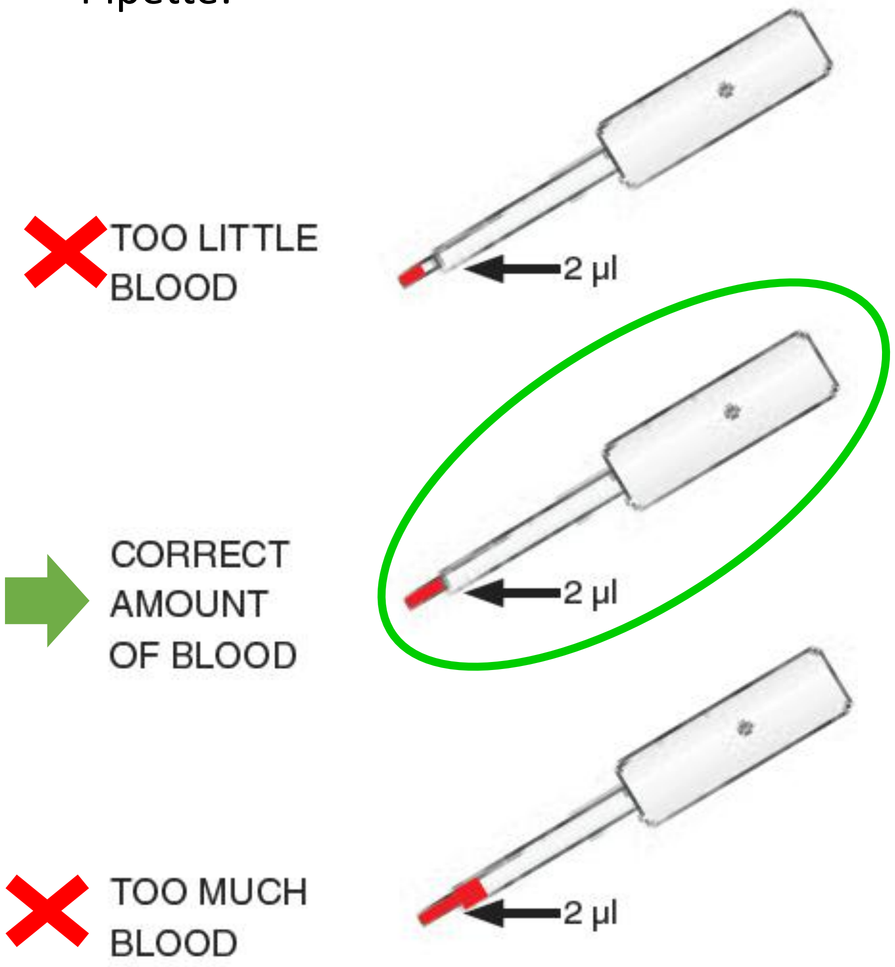

2. How to use the rapid test
# - Performing the test

2.9. Transfer the blood to the test device

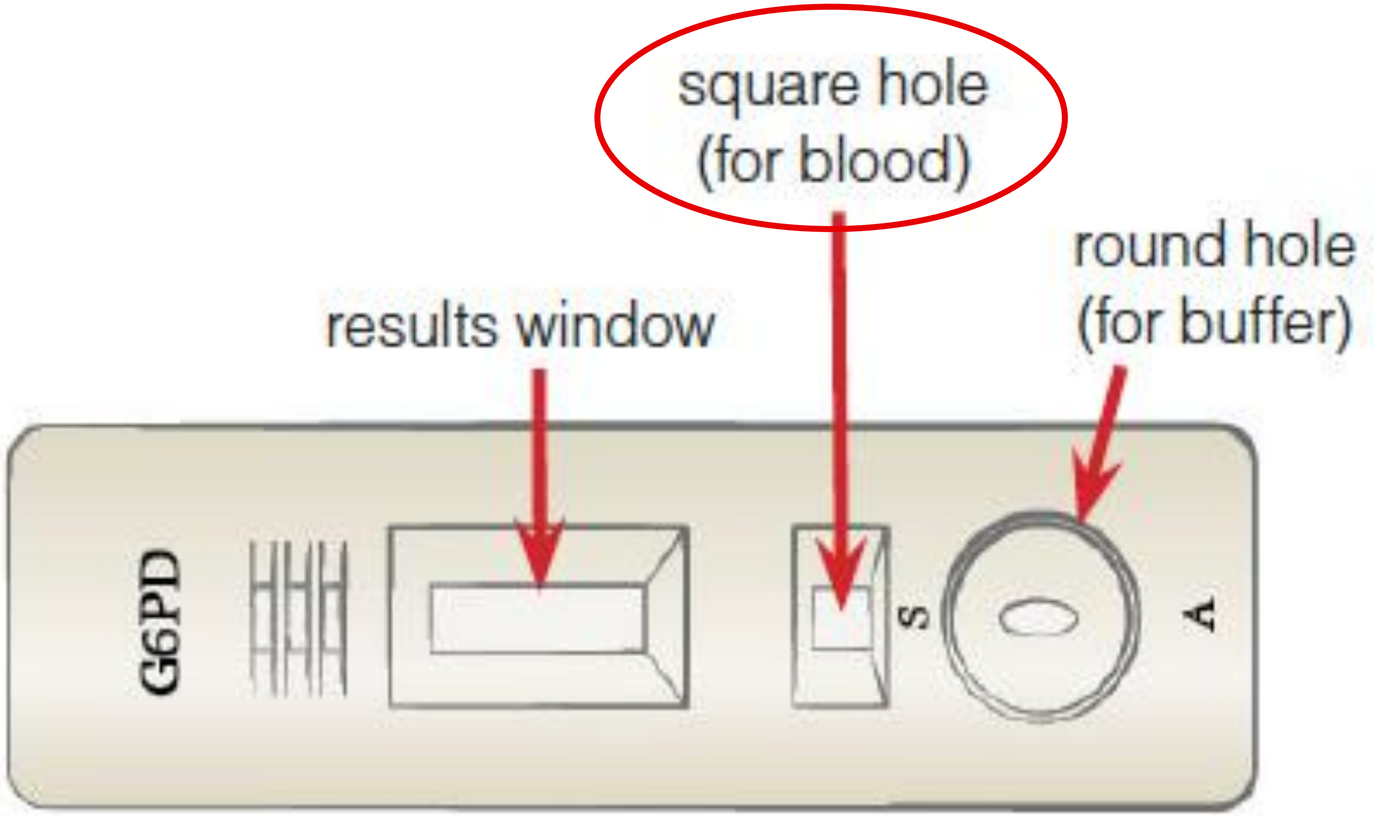

2. How to use the rapid test
# - Performing the test

2.9. Transfer the blood to the test device

- ✓Place the pipette on the membrane of the SQUARE window for Blood(**Letter ”S”**)
- ✓Press the top of the pipette to transfer the blood
- ✓Check that blood is in contact with the blood window membrane
- ✓Discard the pipette after use

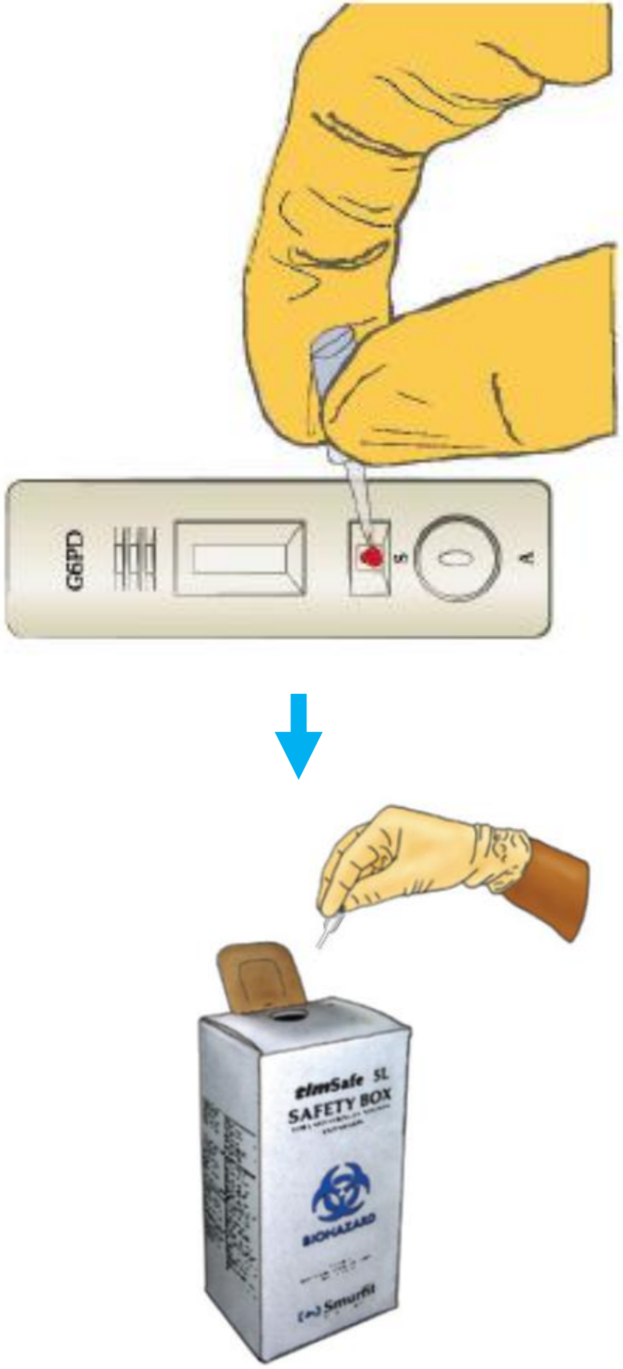

2. How to use the rapid test
# - Performing the test

2.10. Add the reagent

✓Add **TWO drops** of reagent to the **round window (Letter ”A”)**

2.11. Wait for the correct time before reading the result

✓Note on the paper the time you added the reagent and the time you should read the test OR measure the time

✓**Wait <u>at least 10 minutes</u> and at most 20 minutes**

✓Watch the blood flow to the result window and then disappear

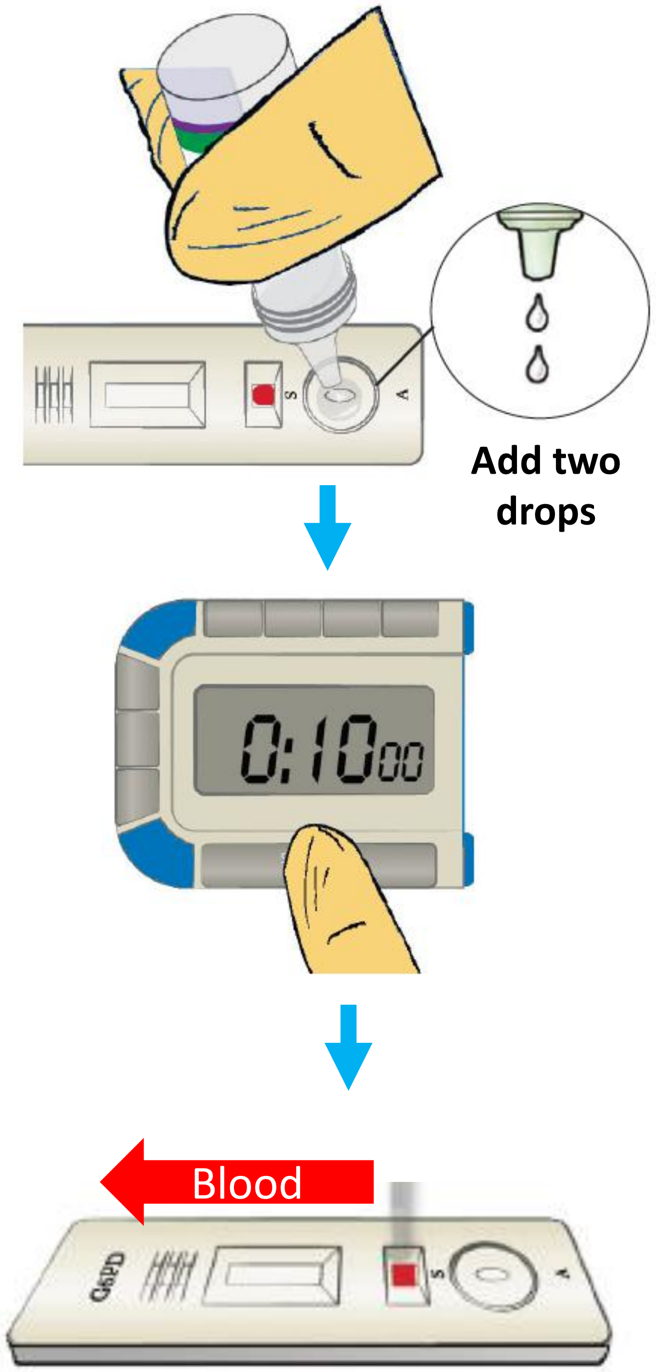

✓Watch the blood flow to the result window and then disappear

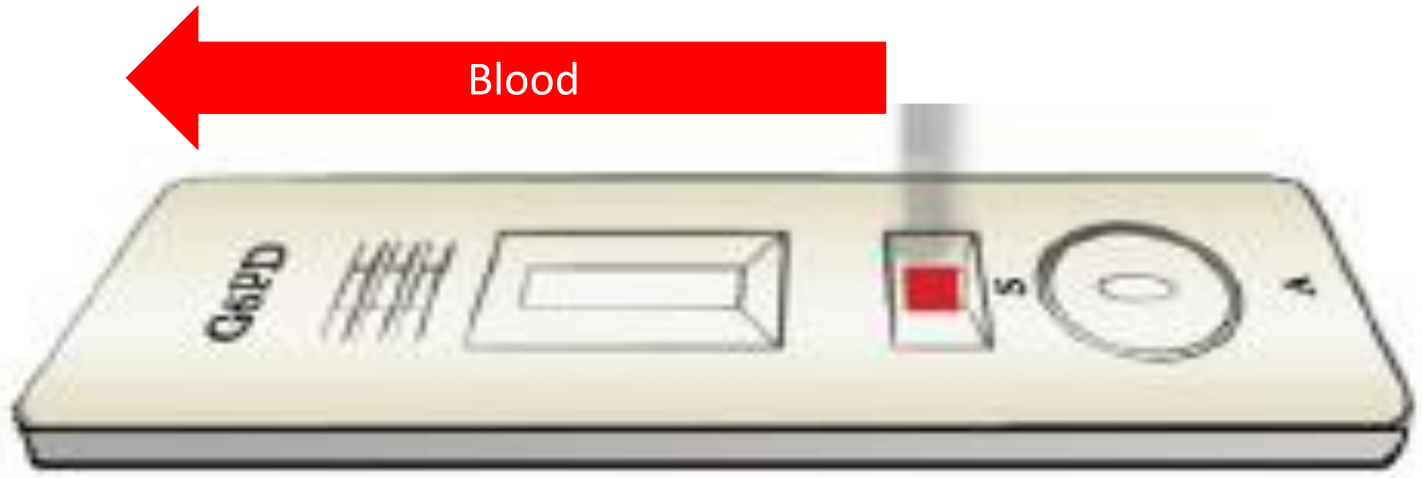

2. How to use the rapid test
# - Performing the test

2.12. Read the test result

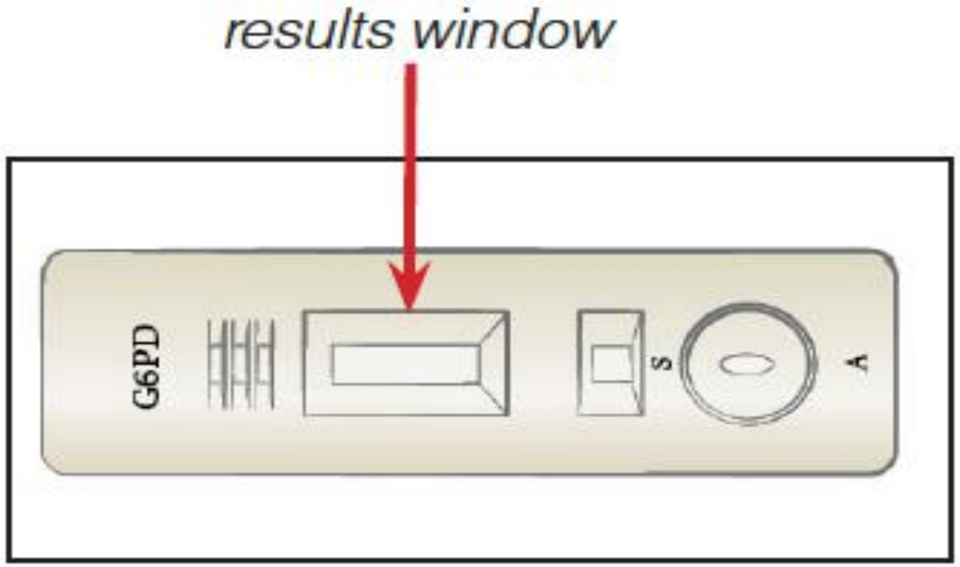

2.13. Dispose of gloves and test in a contaminated material bin

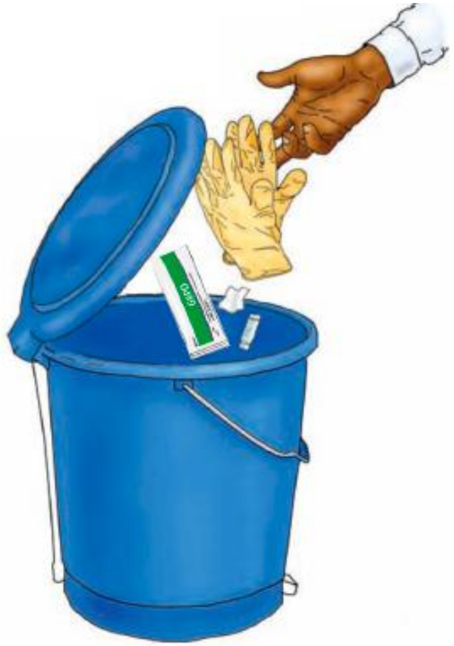

Training video

2. How to use the rapid test

WHAT SHOULD BE REMEMBERED WHEN USING THE RAPID TEST:

- Take proper care when handling blood and disposing of material.
- Follow product guidelines correctly.
- The test should be discarded if the envelope is damaged.
- Open the envelope and use it immediately after opening.
- Tests must not be reused.

2. How to use the rapid test
# - Reading and interpretation of results

➢ POSSIBLE RESULTS

The result is given by the presence of color or its absence.

INVALID TEST

Blood does not flow in the test window, as shown in the following example:

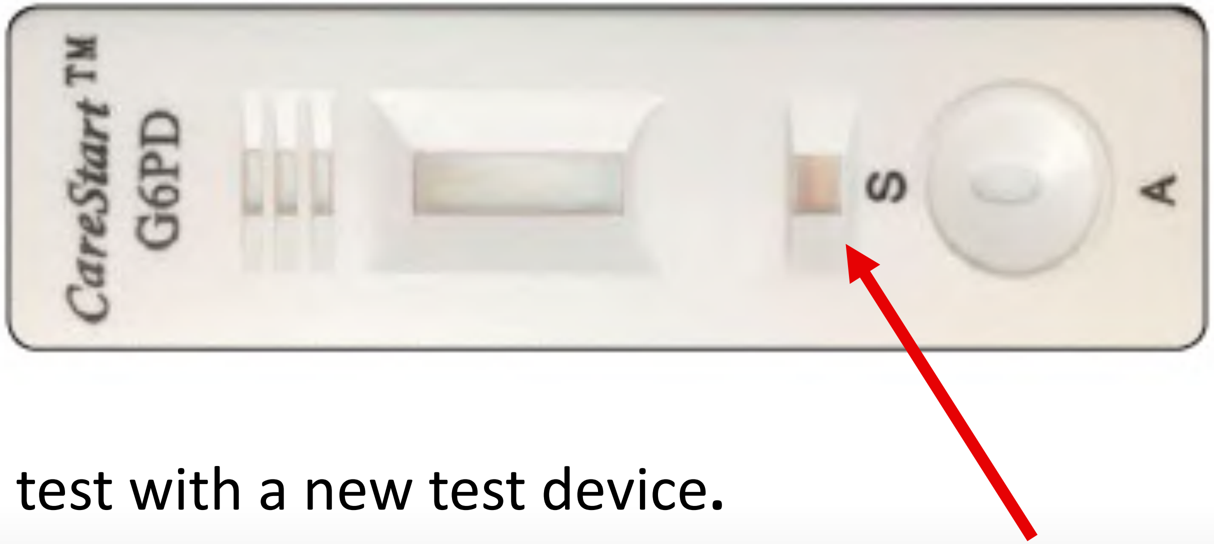

❑ Repeat the test with a new test device.

Reading and interpretation of results

CAN TAKE PRIMAQUINE ON A DAILY BASIS

A **strong purple color** means that the patient **CAN** take primaquine on a daily basis (7 days):

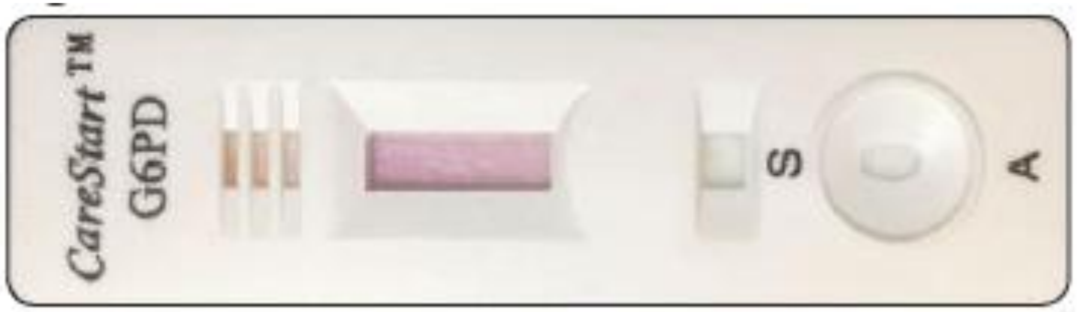

CANNOT TAKE PRIMAQUINE ON A DAILY BASIS

No color change or doubts regarding color (very weak change) means that **CANNOT** use primaquine on a daily basis:

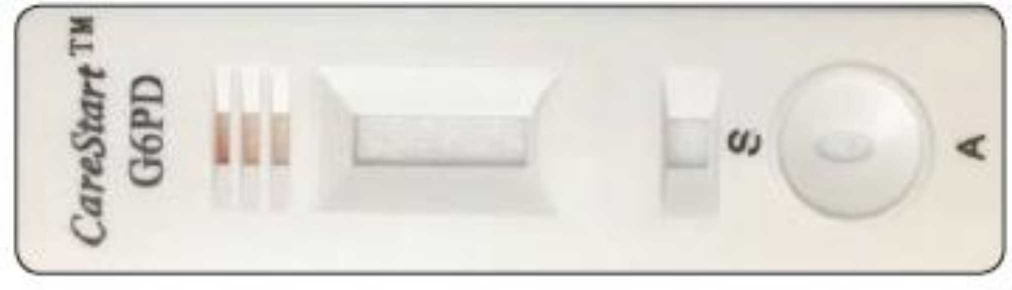

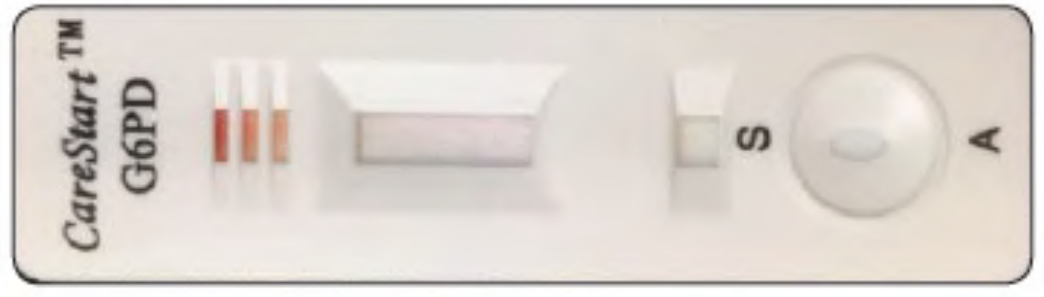

DECISION ALGORITHM

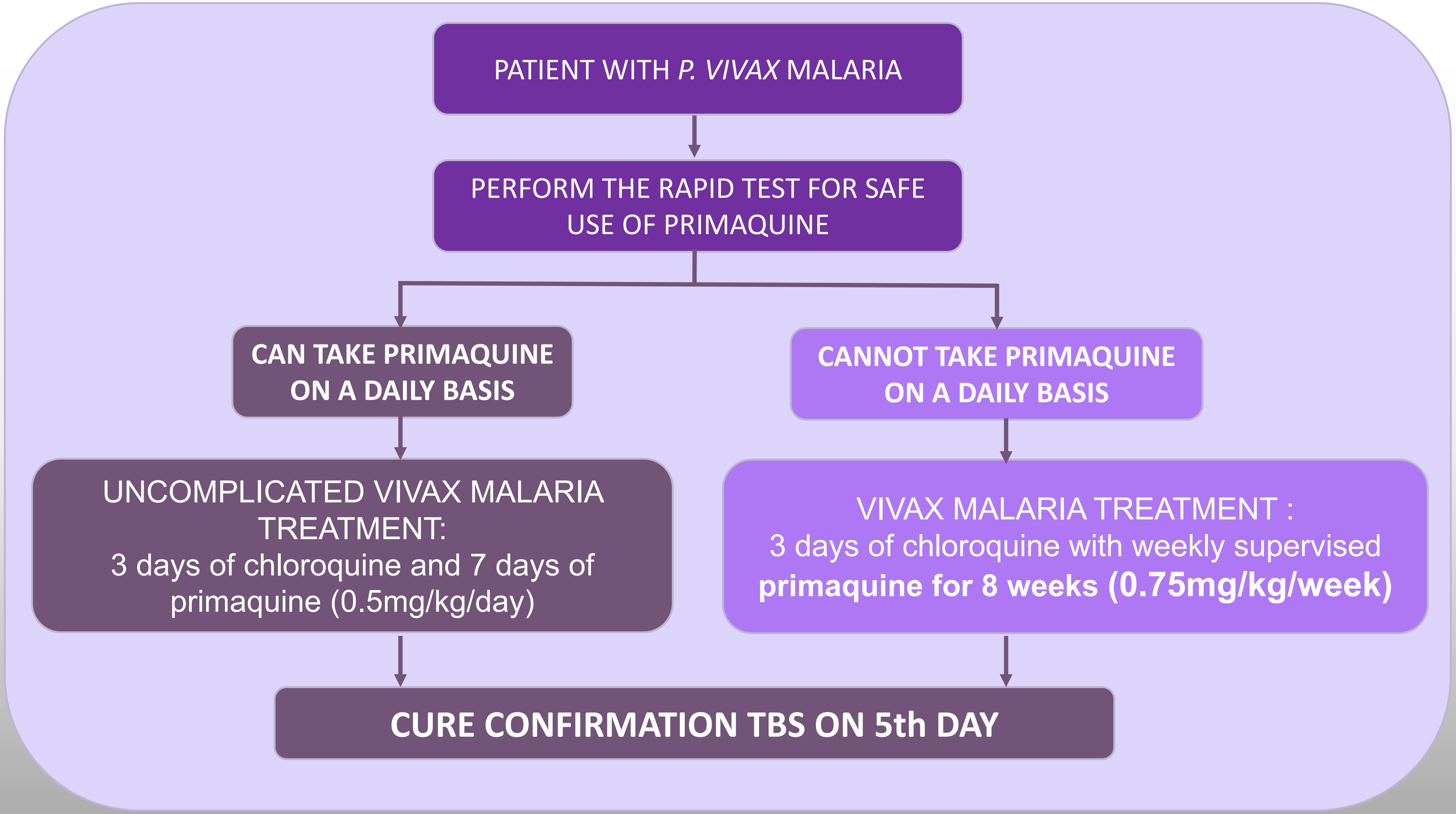

2. How to use the rapid test
# - Reading and interpretation of results

CAN TAKE PRIMAQUINE ON A DAILY BASIS

Strong purple color means the patient **CAN** use the daily primaquine:

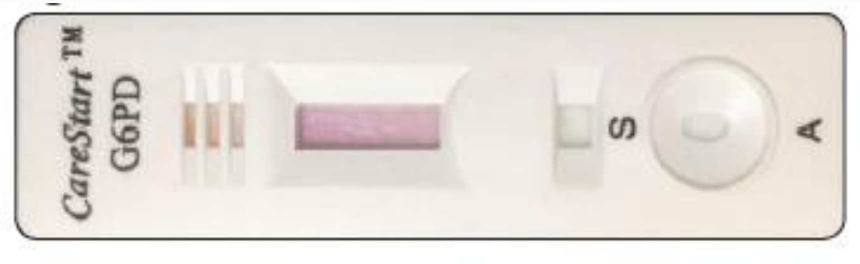

PRIMAQUINE

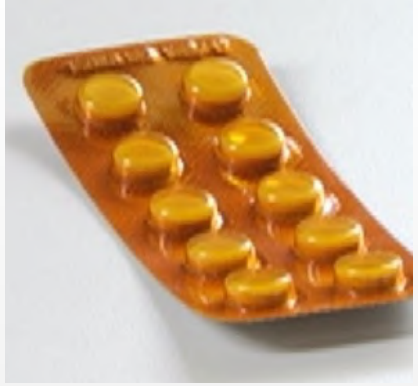

\+

CHLOROQUINE

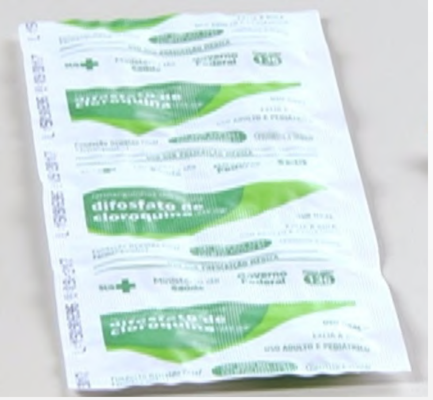

2. How to use the rapid test
# - Reading and interpretation of results

CANNOT TAKE PRIMAQUINE ON A DAILY BASIS

No color change or doubts regarding color (very weak) means that the patient **CANNOT** take primaquine on a daily basis:

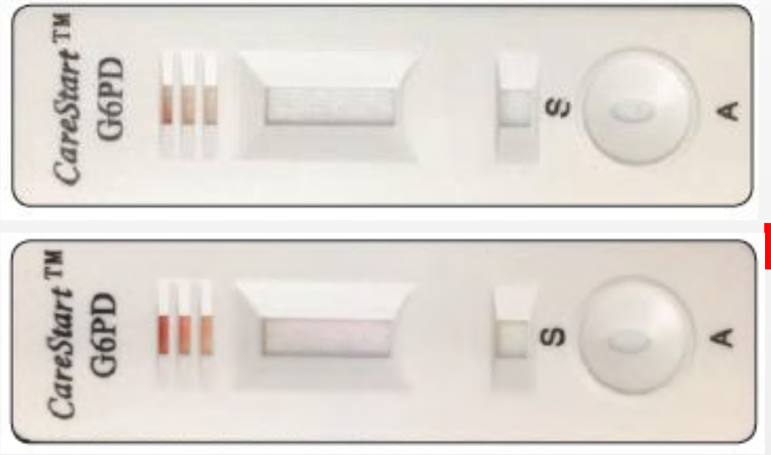

CHLOROQUINE

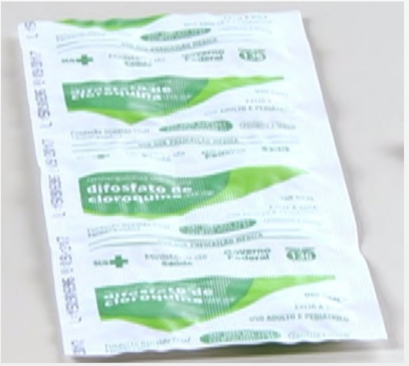

+

WEEKLY SUPERVISED PRIMAQUINE

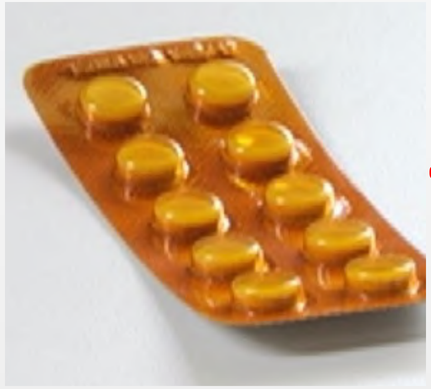

0.75mg/kg/week
1 dose per week
for 8 weeks

+

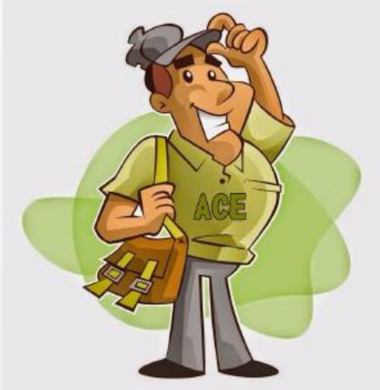

Ex. Patient weighs 70kg
75 x 0.75= 56.5mg
56.5/15mg= 3.75 =
4 tabs/15mg for week

3. Filling in the SIVEP Form

Objectives:

- ✓ Register the result of the rapid test for the safe use of primaquine
- ✓ Perform the Pharmacovigilance relating to the use of primaquine (adverse event)

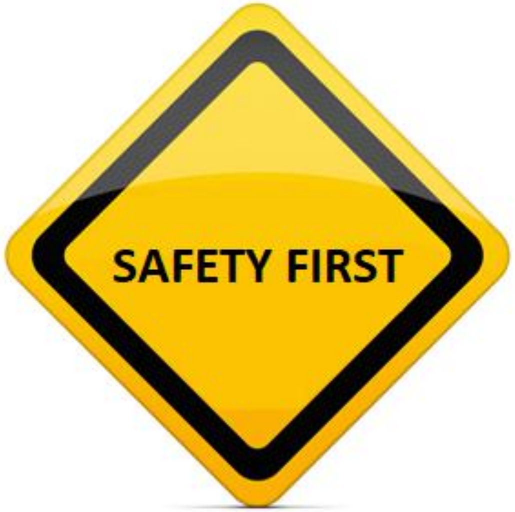

3. Filling in the SIVEP Form

What is Pharmacovigilance?

- "The science and activities related to **identification, evaluation, understanding and prevention of adverse effects or any problems related to the use of medicines**" (ANVISA).

- Pharmacovigilance is responsible for identifying, evaluating and monitoring the occurrence of **adverse events related to use of medications** in the Brazilian market, with the objective of **ensuring that the benefits related to the use of these products outweigh the risks caused by them**.

3. Filling in the SIVEP Form

TREATMENT

46 Course of treatment used according to the Malaria Therapy Manual:

1 – P. vivax or P. ovale infections with chloroquine for 3 days and primaquine for 7 days (short course);
2 – P. vivax, or P. ovale infections with chloroquine for 3 days and primaquine for 14 days (long course);
3 – P. malariae infections for all ages and P. vivax or P. ovale infections in pregnant women and children under 6 months of age with chloroquine for 3 days;
4 – Prevention of frequent relapses by P. vivax or P. ovale with weekly chloroquine for 12 weeks;
5 – P. falciparum infections with the fixed combination of artemether + lumefantrine for 3 days;
6 – P. falciparum infections with fixed combination of artesunate + mefloquine for 3 days;
7 – P. falciparum infections with quinine for 3 days, doxycycline for 5 days and primaquine for 6 days;
8 – Mixed infections by P. falciparum and P. vivax or P. ovale with Artemether + Lumefantrina or Artesunate + Mefloquine for 3 days and Primaquine for 7 days;
9 – Uncomplicated P. falciparum infections in the first trimester of pregnancy and in children under 6 months with quinine for 3 days and clindamycin for 5 days;
10 – Severe and complicated P. falciparum malaria in all age groups;
11 – P. falciparum infections with the fixed combination of artemether + lumefantrine for 3 days and single dose primaquine;
12 – P. falciparum infections with the fixed combination of artesunate + mefloquine for 3 days and single dose primaquine;
13 – P. vivax or P. ovale infections with chloroquine for 3 days and weekly primaquine for 8 weeks under medical supervision
99 – Other course used (by doctor) - describe:

47 Treatment Start Date:

RAPID TEST FOR USE OF PRIMAQUINE

48 Rapid test result for use of Primaquine: 1-CAN use primaquine daily a 2-CANNOT use primaquine daily 3-No test done

49 Source of information: 1-RDT 2-Malaria Info Card

50 Has the patient received a blood transfusion in the last 60 days? 1-Yes 2-No

Fill in spaces 51 and 52 only on patient's return on day 5 of treatment

51 Date of last positive malaria test:

52 Has the patient reported/presented dark urine or yellow eyes? 1-Yes 2-No 3-Other - describe:

53 Patient's weight (Kg):

54 Patient's telephone number:

STATE CODE MUNICIPALITY DEPARTMENT DISTRICT HEALTH

12 Patient's name:

15 Age:

1 Notification No:

38 Test Date:

40 Test Results:

44 Tester's name:

48 Rapid test result for the use of Primaquine 1-CAN use primaquine daily a 2-CANNOT use primaquine daily 3-No test done

Proof of exam result to be given to the patient

PATIENT, PLEASE NOTE: If you have **dark urine (coca cola color) or yellow eyes,** seek for medial assistance.

TREATMENT:
Field 46, item 13

RAPID TEST FOR THE SAFE USE OF PRIMAQUINE:
Fields 48-54

PATIENT'S PART OF THE FORM:
Field 48

3. Filling in the SIVEP Form

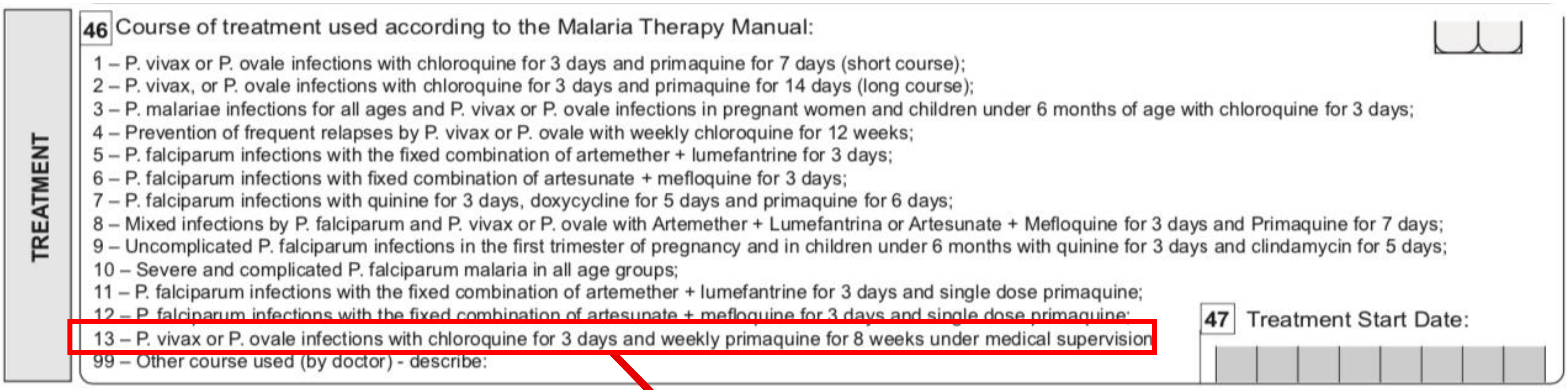

TREATMENT

46 Course of treatment used according to the Malaria Therapy Manual:

1 – P. vivax or P. ovale infections with chloroquine for 3 days and primaquine for 7 days (short course);
2 – P. vivax, or P. ovale infections with chloroquine for 3 days and primaquine for 14 days (long course);
3 – P. malariae infections for all ages and P. vivax or P. ovale infections in pregnant women and children under 6 months of age with chloroquine for 3 days;
4 – Prevention of frequent relapses by P. vivax or P. ovale with weekly chloroquine for 12 weeks;
5 – P. falciparum infections with the fixed combination of artemether + lumefantrine for 3 days;
6 – P. falciparum infections with fixed combination of artesunate + mefloquine for 3 days;
7 – P. falciparum infections with quinine for 3 days, doxycycline for 5 days and primaquine for 6 days;
8 – Mixed infections by P. falciparum and P. vivax or P. ovale with Artemether + Lumefantrina or Artesunate + Mefloquine for 3 days and Primaquine for 7 days;
9 – Uncomplicated P. falciparum infections in the first trimester of pregnancy and in children under 6 months with quinine for 3 days and clindamycin for 5 days;
10 – Severe and complicated P. falciparum malaria in all age groups;
11 – P. falciparum infections with the fixed combination of artemether + lumefantrine for 3 days and single dose primaquine;
12 – P. falciparum infections with the fixed combination of artesunate + mefloquine for 3 days and single dose primaquine;
13 – P. vivax or P. ovale infections with chloroquine for 3 days and weekly primaquine for 8 weeks under medical supervision
99 – Other course used (by doctor) - describe:

47 Treatment Start Date:

TREATMENT:

Field 46, item 13: ***P. vivax*** **or** ***P. ovale*** **infections must be treated with chloroquine for 3 days and primaquine weekly for 8 weeks under medical supervision**

*** When the test result is positive: Cannot take primaquine on a daily basis**

3. Filling in the SIVEP Form

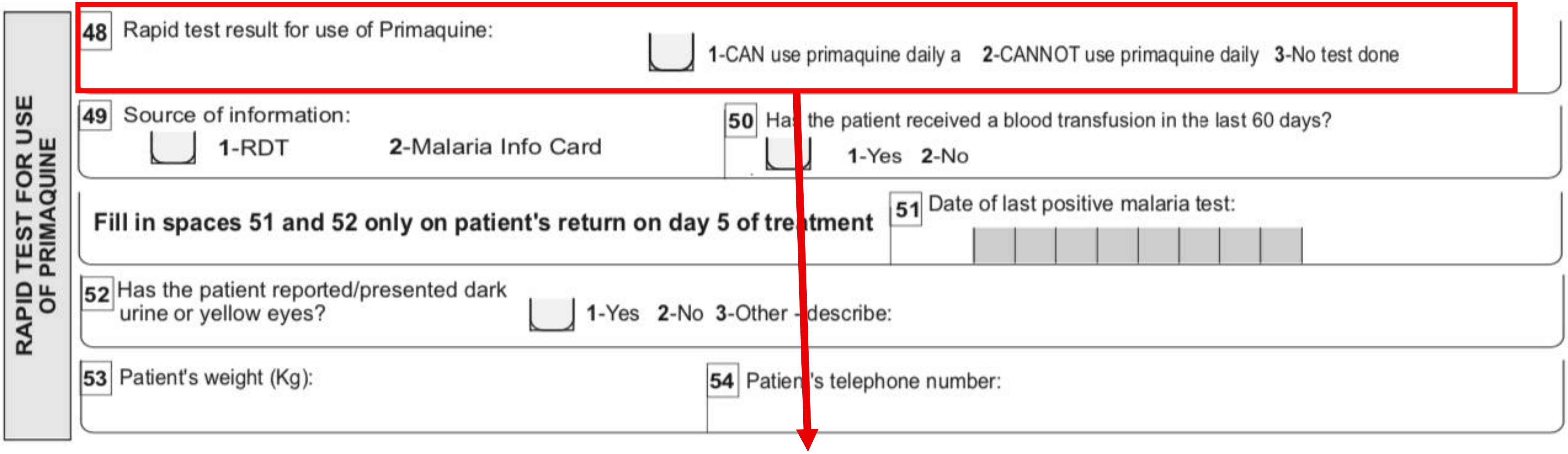

RAPID TEST FOR USE OF PRIMAQUINE

48 Rapid test result for use of Primaquine: 1-CAN use primaquine daily a 2-CANNOT use primaquine daily 3-No test done

49 Source of information: 1-RDT 2-Malaria Info Card

50 Has the patient received a blood transfusion in the last 60 days? 1-Yes 2-No

Fill in spaces 51 and 52 only on patient's return on day 5 of treatment

51 Date of last positive malaria test:

52 Has the patient reported/presented dark urine or yellow eyes? 1-Yes 2-No 3-Other - describe:

53 Patient's weight (Kg):

54 Patient's telephone number:

RAPID TEST FOR THE SAFE USE OF PRIMAQUINE:

Field 48: Result of the rapid test for the safe use of primaquine:

1- **CAN** TAKE PRIMAQUINE ON A DAILY BASIS

2- **CANNOT** TAKE PRIMAQUINE ON A DAILY BASIS

3- Not tested

3. Filling in the SIVEP Form

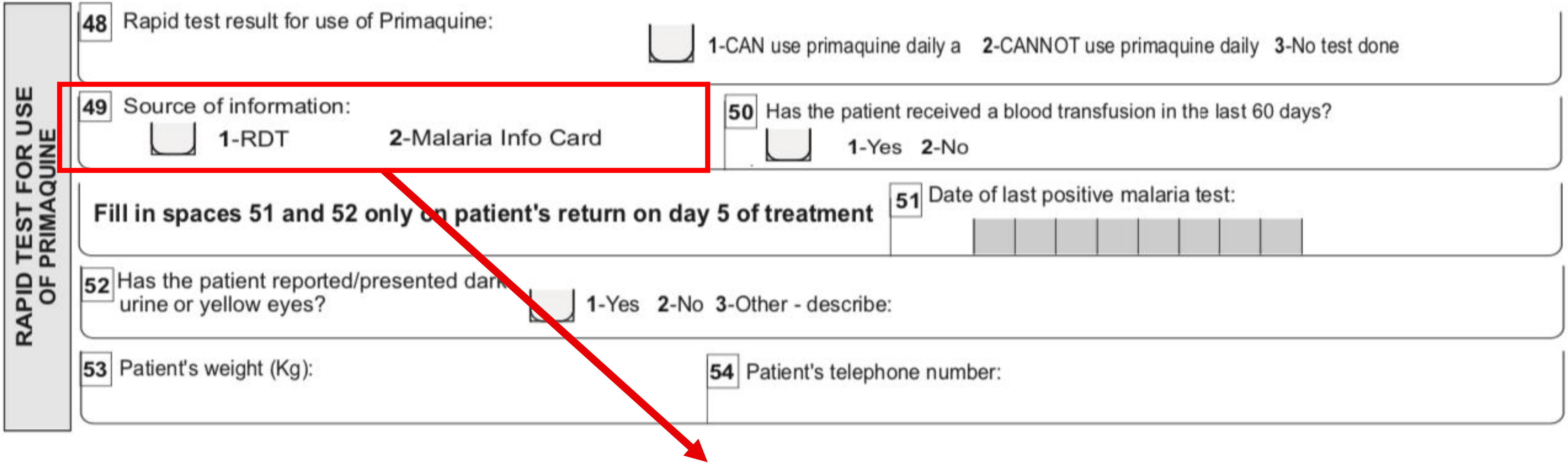
RAPID TEST FOR USE OF PRIMAQUINE

48 Rapid test result for use of Primaquine: 1-CAN use primaquine daily a 2-CANNOT use primaquine daily 3-No test done

49 Source of information: 1-RDT 2-Malaria Info Card

50 Has the patient received a blood transfusion in the last 60 days? 1-Yes 2-No

Fill in spaces 51 and 52 only on patient's return on day 5 of treatment

51 Date of last positive malaria test:

52 Has the patient reported/presented dark urine or yellow eyes? 1-Yes 2-No 3-Other - describe:

53 Patient's weight (Kg):

54 Patient's telephone number:

RAPID TEST FOR THE SAFE USE OF PRIMAQUINE:

Field 49: Source of information:

1- Rapid test

2- Malaria Info Card / SIVEP (Patient's part of the form)

3. Filling in the SIVEP Form

MALARIA INFO CARD

PURPLE card: Test result – **CAN** TAKE PRIMAQUINE ON A DAILY BASIS

GRAY card: Test result – **CANNOT** TAKE PRIMAQUINE ON A DAILY BASIS

FMT-HVD

Instituto ELIMINA

ILMD
INSTITUTO LEÔNIDAS & MARIA DEANE
Fiocruz Amazônia

Fundação de Medicina Tropical Dr. Heitor Vieira Dourado

Malaria Info Card

Name: ____________________

Weight (Kg): ____________________

Date of Birth: ____/____/____

Address: ____________________

____

Mother's name: ____________________

Phone number: ____________________

RESULTS OF THE RAPID TEST FOR SAFE USE OF PRIMAQUINE

This patient has had the G6PD test and **CAN** use daily primaquine.

Test date: 01 / 01 / 2018

Name of clinic: MARIA DE LOUDES I

HCP: JOSE ANTONIO

Date of malaria test	Result	Treatment	Return date (Return on day 5 of treatment)	Signs and symptoms
01 / 01 / 2018	[X] *VIVAX*<br>☐ *FALCIPARUM*<br>☐ *MIXED INFEC.*	Chloroquine for 3 days<br>Primaquine por 7 days	05 / 01 / 2018	☐ *Dark urine*<br>☐ *Yellow eyes and or skin*<br>☐ *Others, please list:*
___/___/___	☐ *VIVAX*<br>☐ *FALCIPARUM*<br>☐ *MIXED INFEC.*		___/___/___	☐ *Dark urine*<br>☐ *Yellow eyes and or skin*<br>☐ *Others, please list:*
___/___/___	☐ *VIVAX*<br>☐ *FALCIPARUM*<br>☐ *MIXED INFEC.*		___/___/___	☐ *Dark urine*<br>☐ *Yellow eyes and or skin*<br>☐ *Others, please list:*
___/___/___	☐ *VIVAX*<br>☐ *FALCIPARUM*<br>☐ *MIXED INFEC.*		___/___/___	☐ *Dark urine*<br>☐ *Yellow eyes and or skin*<br>☐ *Others, please list:*

- If your **urine becomes dark, similar to Coca Cola, your eyes or skin become yellow**, seek medical attention.
- **Do not stop taking your medicine**, even if you are feeling better.
- Always **show this card to the nurse or your doctor**, if you are suspected of having malaria.

FMT-HVD

Instituto ELIMINA

ILMD
INSTITUTO LEÔNIDAS & MARIA DEANE
Fiocruz Amazônia

Fundação de Medicina Tropical Dr. Heitor Vieira Dourado

Malaria Info Card

Name:______________________________

Weight (Kg):______________________________

Date of Birth: _____/_____/_____

Address:______________________________

____

Mother's name:______________________________

Phone number:______________________________

RESULTS OF THE RAPID TEST FOR SAFE USE OF PRIMAQUINE:

This patient has had the G6PD test and **CANNOT** use daily primaquine.

Test date: 01 / 01 / 2018
Name of clinic: MARIA DE LOUDES I
HCP: JOSE ANTONIO

Date of malaria test	Result	Treatment	Return date (Return on day 5 of treatment)	Signs and symptoms
01 / 01 / 2018	[x] *VIVAX* [ ] *FALCIPARUM* [ ] *MIXED INFEC.*	Chloroquine for 3 days Primaquine weekly for 8 weeks	05 / 01 / 2018	[ ] *Dark urine* [ ] *Yellow eyes and or skin* [ ] *Others, please list:*
__/__/__	[ ] *VIVAX* [ ] *FALCIPARUM* [ ] *MIXED INFEC.*		__/__/__	[ ] *Dark urine* [ ] *Yellow eyes and or skin* [ ] *Others, please list:*
__/__/__	[ ] *VIVAX* [ ] *FALCIPARUM* [ ] *MIXED INFEC.*		__/__/__	[ ] *Dark urine* [ ] *Yellow eyes and or skin* [ ] *Others, please list:*
__/__/__	[ ] *VIVAX* [ ] *FALCIPARUM* [ ] *MIXED INFEC.*		__/__/__	[ ] *Dark urine* [ ] *Yellow eyes and or skin* [ ] *Others, please list:*

- If your **urine becomes dark, similar to Coca Cola, your eyes or skin become yellow**, seek medical attention.
- **Do not stop taking your medicine**, even if you are feeling better.
- Always **show this card to the nurse or your doctor**, if you are suspected of having malaria.

3. Filling in the SIVEP Form

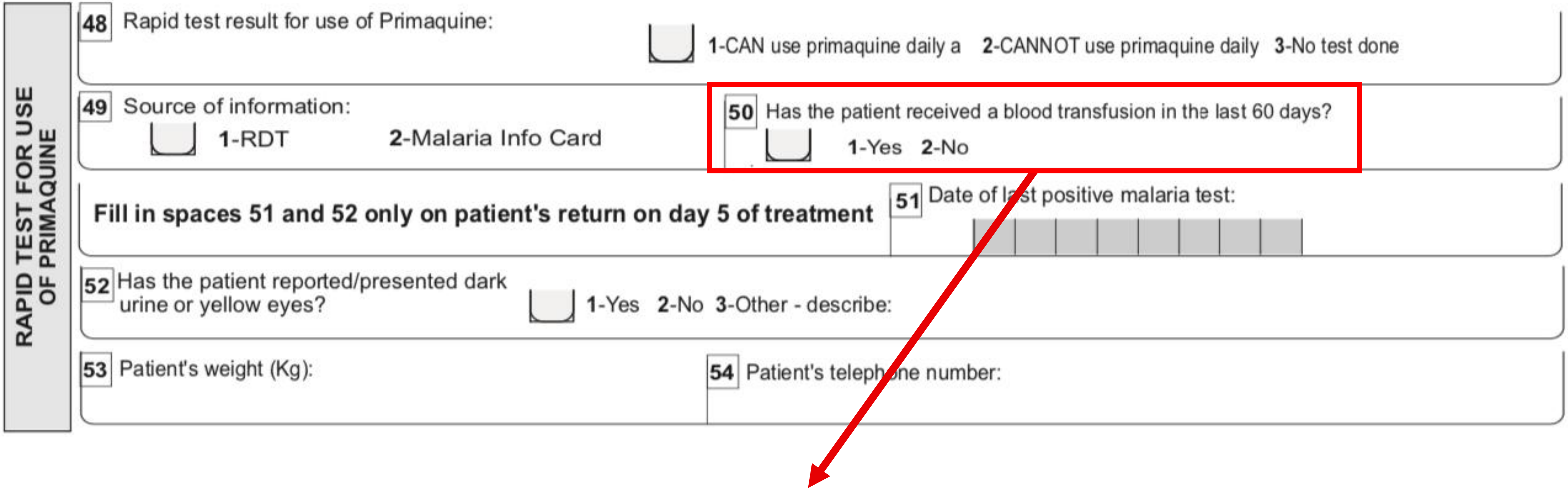

RAPID TEST FOR USE OF PRIMAQUINE

48 Rapid test result for use of Primaquine: 1-CAN use primaquine daily a 2-CANNOT use primaquine daily 3-No test done

49 Source of information: 1-RDT 2-Malaria Info Card

50 Has the patient received a blood transfusion in the last 60 days? 1-Yes 2-No

Fill in spaces 51 and 52 only on patient's return on day 5 of treatment

51 Date of last positive malaria test:

52 Has the patient reported/presented dark urine or yellow eyes? 1-Yes 2-No 3-Other - describe:

53 Patient's weight (Kg):

54 Patient's telephone number:

RAPID TEST FOR THE SAFE USE OF PRIMAQUINE:

Field 50: Has the patient had a blood transfusion in the last 60 days :

1- Yes

2- No

3. Filling in the SIVEP Form

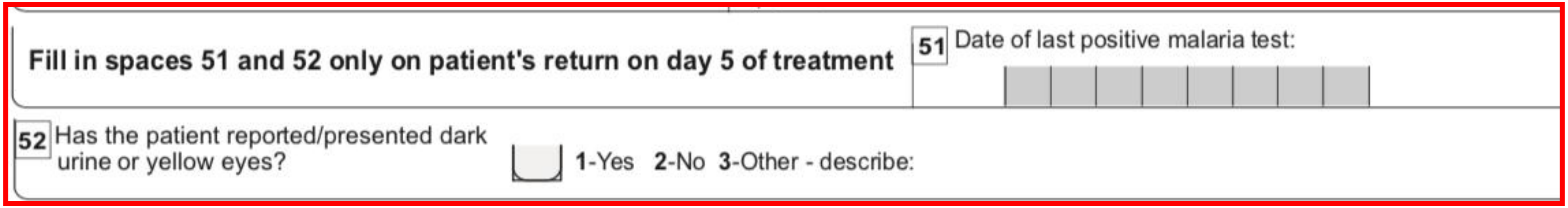
Fill in spaces 51 and 52 only on patient's return on day 5 of treatment

51 Date of last positive malaria test:

52 Has the patient reported/presented dark urine or yellow eyes? 1-Yes 2-No 3-Other - describe:

RAPID TEST FOR THE SAFE USE OF PRIMAQUINE:

To be filled in on day 5 after treatment begins

Field 51: **Date of the last positive malaria test**

Field 52: **Presented with dark urine or yellow eyes**

1- Yes

2- No

3- Others – Please describe

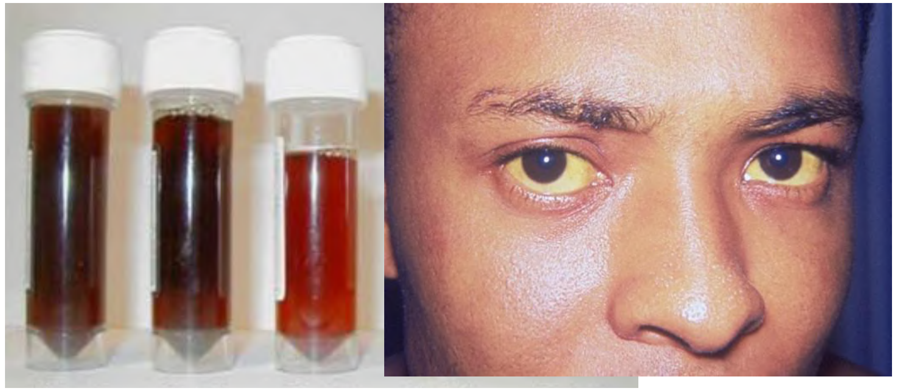

3. Filling in the SIVEP Form

53 Patient's weight (kg)

54 Patient's phone number:

RAPID TEST FOR THE SAFE USE OF PRIMAQUINE:

Field 53: **Patient’s weight**
Field 54: **Patient’s phone number**

3. Filling in the SIVEP Form

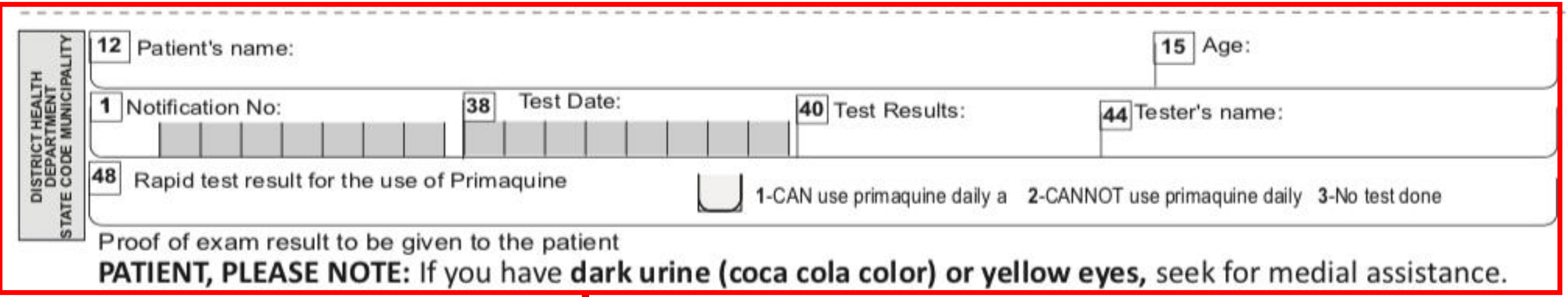

DISTRICT HEALTH DEPARTMENT
STATE CODE MUNICIPALITY

12 Patient's name:

15 Age:

1 Notification No:

38 Test Date:

40 Test Results:

44 Tester's name:

48 Rapid test result for the use of Primaquine

1-CAN use primaquine daily a 2-CANNOT use primaquine daily 3-No test done

Proof of exam result to be given to the patient

PATIENT, PLEASE NOTE: If you have **dark urine (coca cola color) or yellow eyes,** seek for medial assistance.

PATIENT'S PART OF THE FORM:

Field 48: **Results of the rapid test for the safe use of primaquine**

3. Filling in the SIVEP Form

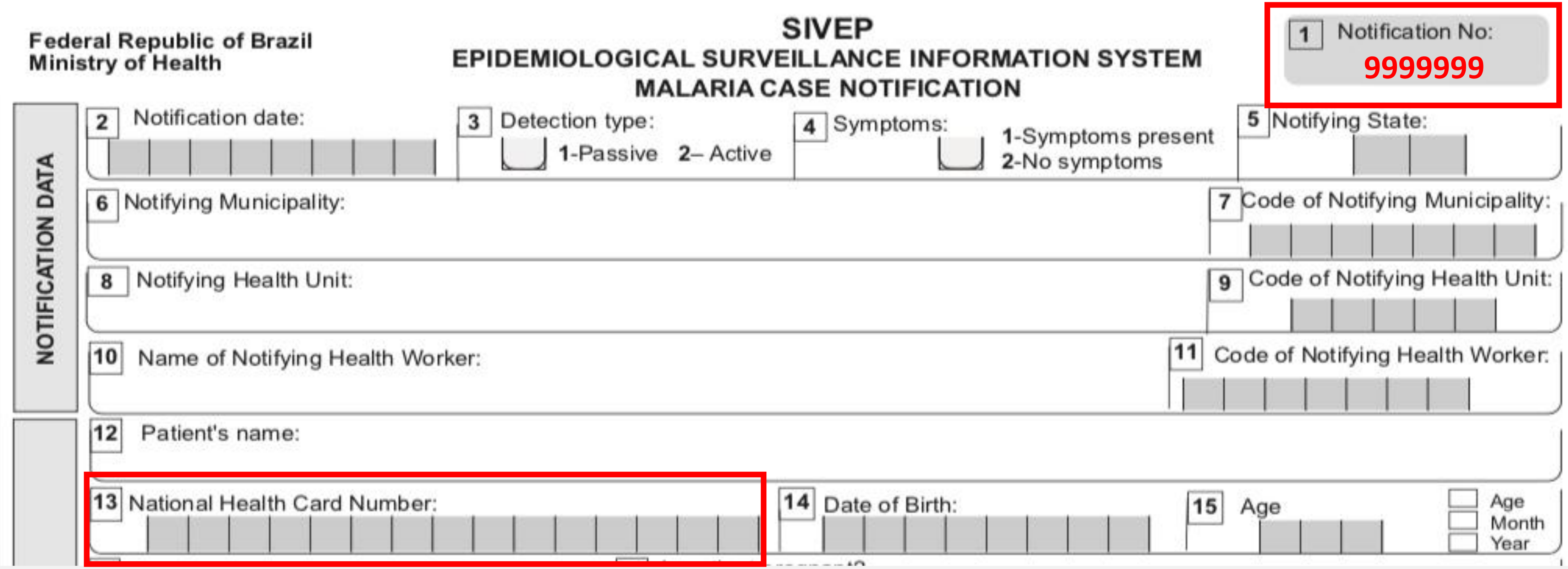
Federal Republic of Brazil
Ministry of Health

SIVEP
EPIDEMIOLOGICAL SURVEILLANCE INFORMATION SYSTEM
MALARIA CASE NOTIFICATION

1 Notification No: 9999999

NOTIFICATION DATA

2 Notification date:

3 Detection type: 1-Passive 2– Active

4 Symptoms: 1-Symptoms present 2-No symptoms

5 Notifying State:

6 Notifying Municipality:

7 Code of Notifying Municipality:

8 Notifying Health Unit:

9 Code of Notifying Health Unit:

10 Name of Notifying Health Worker:

11 Code of Notifying Health Worker:

12 Patient's name:

13 National Health Card Number:

14 Date of Birth:

15 Age — Age / Month / Year

OTHER DETAILS ON THE FORM:

Field 1: **Notification number is already on the sheet**

Field 13: **Always fill in the patient’s National Health Card number**

4. PATIENT COUNSELING

- Stress the importance of performing **complete treatment**
- Instruct the patient that whenever he/she has **malaria** they must inform the **doctor** about the **result of the rapid test for safe use of Primaquine.**
- Instruct the patient regarding the need to return on the 5th day to perform the **cure verification thick blood smear.**
- Make sure patient is aware of the signs of hemolytic anemia, such as **dark urine, yellow skin or eyes**.
- At any time, if the patient shows signs or symptoms of severity, inform that he should immediately seek the **referral health unit**.

5. Proficiency test

1

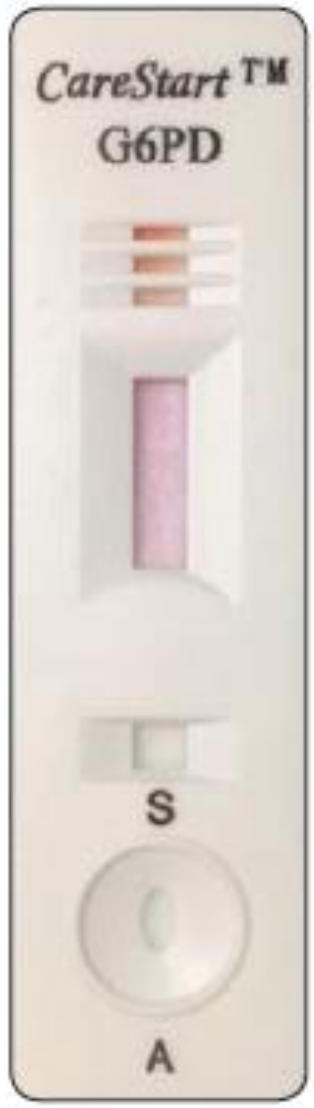

What is the result of this rapid test for the safe use of primaquine?

CAN TAKE DAILY PRIMAQUINE

CANNOT TAKE DIALY PRIMAQUINE

INVALID RESULT

5. Proficiency test

2

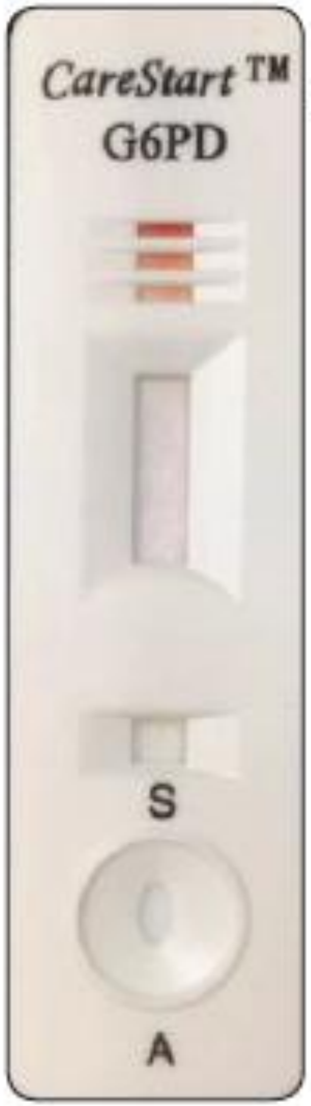

What is the result of this rapid test for the safe use of primaquine?

CAN TAKE DAILY PRIMAQUINE

CANNOT TAKE DAILY PRIMAQUINE

INVALID RESULT

5. Proficiency test

What is the result of this rapid test for the safe use of primaquine?

CAN TAKE PRIMAQUINE

CANNOT TAKE PRIMAQUINE

→ **INVALID RESULT**

3

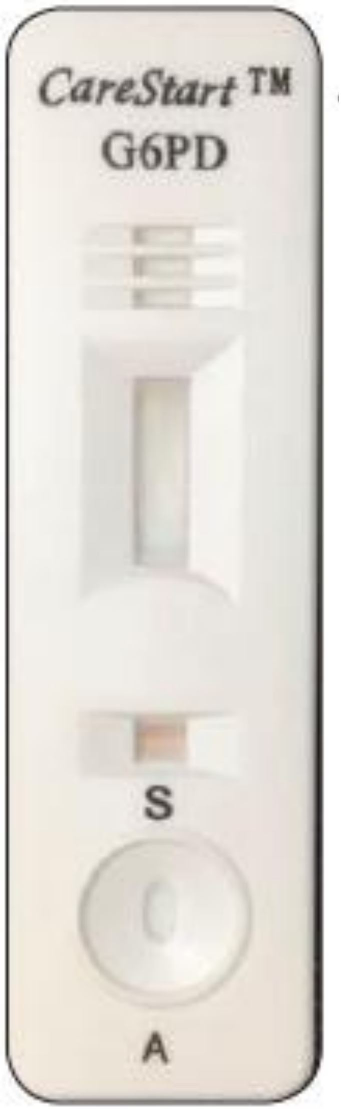

5. Proficiency test

What is the result of this rapid test for the safe use of primaquine?

CAN TAKE DAILY PRIMAQUINE

CANNOT TAKE DAILY PRIMAQUINE

INVALID RESULT

5

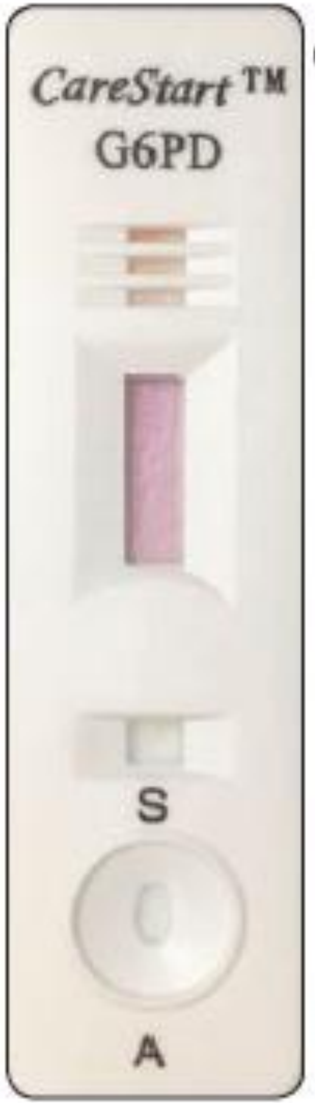

5. Proficiency test

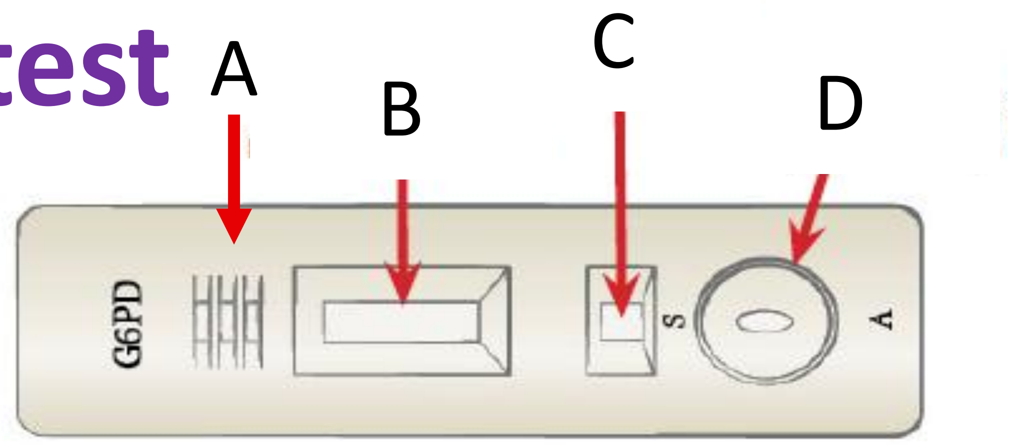

Look at the figure of the rapid test device for safe use of primaquine shown above, and answer:

1) The place where we should observe the **result** of the rapid test for use of primaquine is represented by the letter:

a) A
b) B
c) C
d) D

2) The two drops of the **reagent** must be placed in the location represented by the letter:

a) A
b) B
c) C
d) D

5. Proficiency test

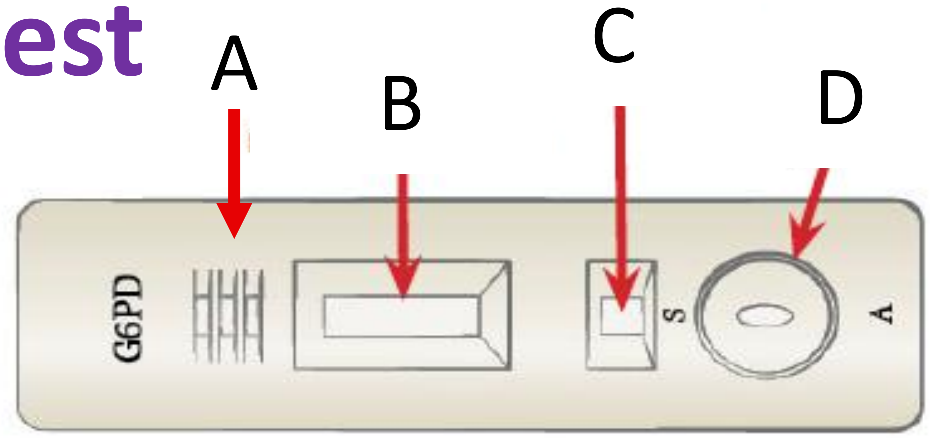

Look at the figure of the rapid test device for safe use of primaquine shown above, and answer:

3) The place where we see **if the blood flowed** in the device is represented by the letter:

a) A
b) B
c) C
d) D

4) The **2µL of blood** collected with the pipette after finger pricking must be added at the location represented by the letter:

a) A
b) B
c) C
d) D

5. Proficiency test

Patient JLX, male, 14 years old, was diagnosed with vivax malaria. The rapid test for the safe use of primaquine was performed for the first time to identify the appropriate treatment regimen. The test result color was white in the result window. Based on the clinical case, answer:

1) After adding the reagent, what is the appropriate time interval for reading the test result?

a) 5 to 15 minutes
b) 10 to 20 minutes
c) 15 to 25 minutes
d) 20 to 25 minutes
e) 25 to 30 minutes

2) The appropriate treatment regimen would therefore be:

a) Chloroquine for 3 days + primaquine daily for 7 days
b) Chloroquine for 3 days + primaquine daily for 14 days
c) Chloroquine for 3 days + primaquine weekly for 8 weeks
d) Chloroquine weekly for 12 weeks
e) Chloroquine for 3 days

5. Proficiency test

Patient JLX, male, 14 years old, was diagnosed with vivax malaria. The rapid test for the safe use of primaquine was performed for the first time to identify the appropriate treatment regimen. The test result was white in the result window. Based on the clinical case, answer:

3) In field 49 of the SIVEP form, you must check the option:

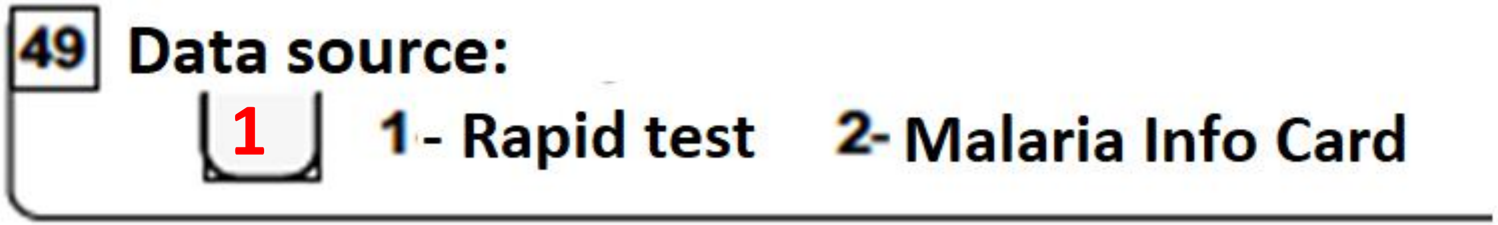
49 Data source:
1 | 1 - Rapid test 2- Malaria Info Card

4) The color of the Malaria Info Card the patient will receive will be:

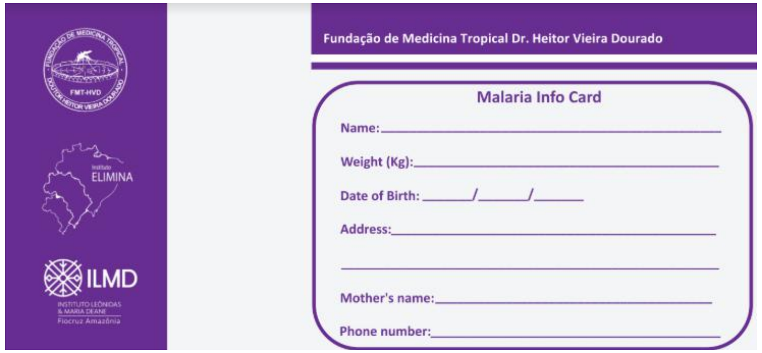

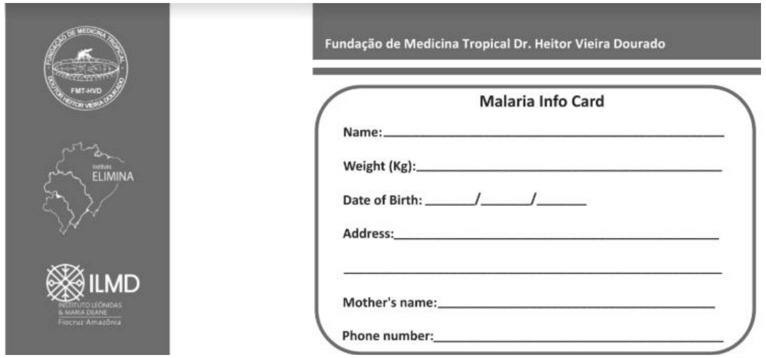

GRAY CARD

5. Proficiency test

The patient AAA, female, 34 years old, was diagnosed with vivax malaria on 11/01/2018. The patient did not take the rapid test for the safe use of primaquine, as it was not yet available. She took chloroquine for 3 days and was taking primaquine daily. On 11/5/2018, the patient returned to the UBS and reported having dark urine, and vomiting. She denies having received a blood transfusion previously. Based on the clinical case, how would you fill in fields 48 to 52 of the SIVEP form:

48 Rapid test result for use of Primaquine: **3** 1-CAN use primaquine daily a 2-CANNOT use primaquine daily 3-No tes **3- Did not do the test**

49 Source of information: 1-I **Do not fill in!!!** nfo Card

50 Has the patient received a blood transfusion in the last 60 days? **2** 1-Yes **2- No**

Fill in spaces 51 and 52 only on patient's return on day 5 of treatment

51 Date of last positive malaria test: **01/11/2018**

52 Has the patient reported/presented dark urine or yellow eyes? **1** **1- Yes** Other - describe:

This is end of the theoretical part and beginning of the practical part!

Thanks!

PRACTICAL PART OF TRAINING:

2. How to use the rapid test
# - Preparation before using the rapid test

2.1. Separate the necessary materials and supplies to perform the rapid test:

- ✓ **Rapid test kit**
  - ✓ Alcohol wipe
  - ✓ Lancet
  - ✓ Reagent
  - ✓ Pipette
  - ✓ Test device

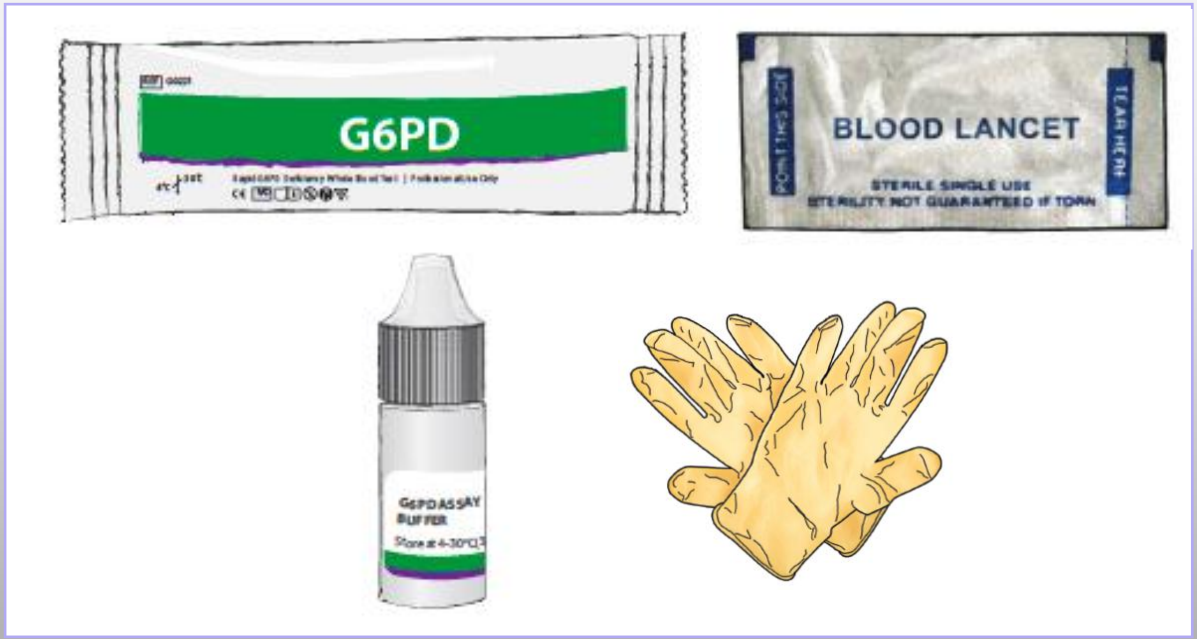

- ✓ One pair of NEW disposable gloves

2. How to use the rapid test
# - Preparation before using the rapid test

2.1. Separate the necessary materials and supplies to perform the rapid test:

In addition to the kit's components, the following materials are required:

- ✓ Sharps disposal container;
- ✓ Common waste disposal container;
- ✓ Pen or pencil
- ✓ Stopwatch

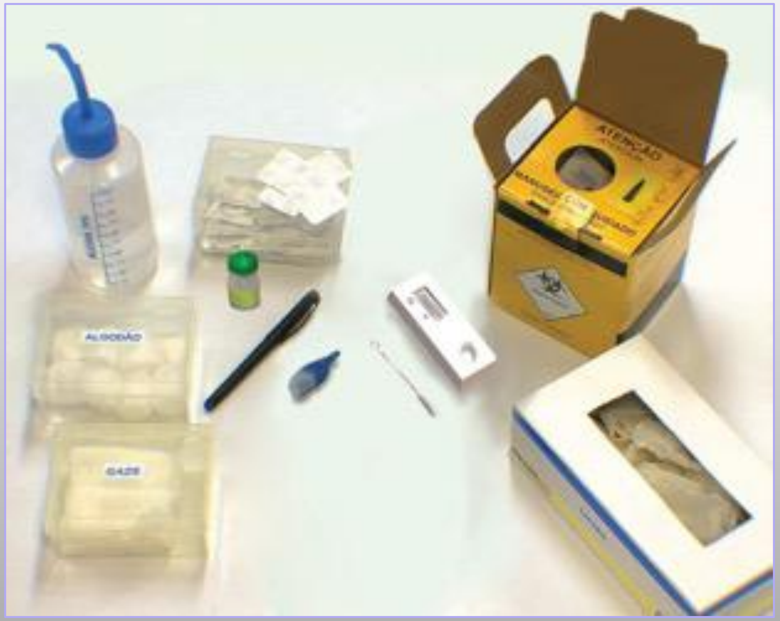

2. How to use the rapid test
# - Preparation before using the rapid test

2.2. Check the test's expiration date:

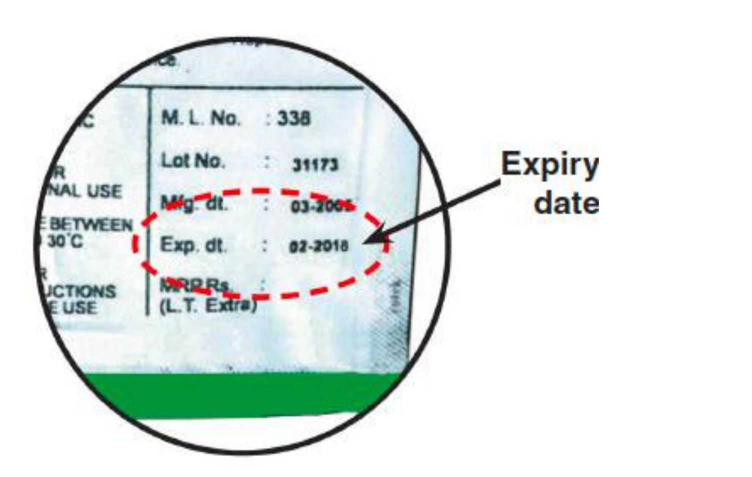

2.3. Professional preparation:

✓ Wash your hands using soap and water, 70% alcohol or other antiseptic and put on gloves.

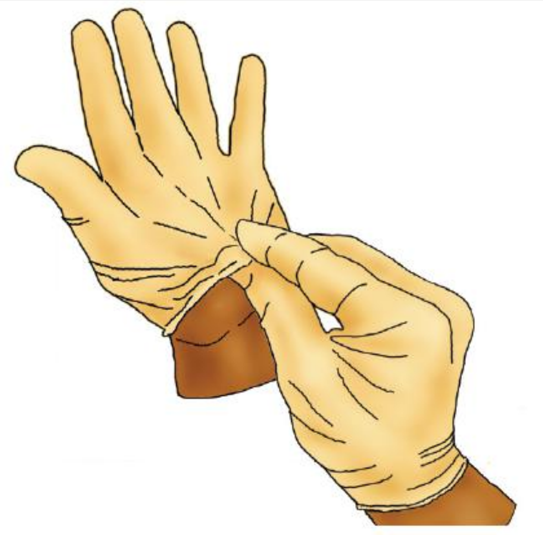

2. How to use the rapid test
# - Preparation before using the rapid test

2.4. Open the test package and remove the contents:

- ✓Test device
- ✓Silica gel sachet (keeps the test dry)
- ✓Pipette

Silica gel

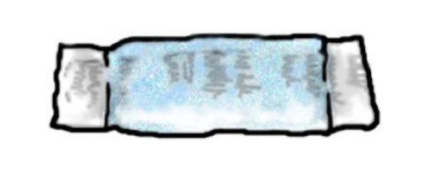

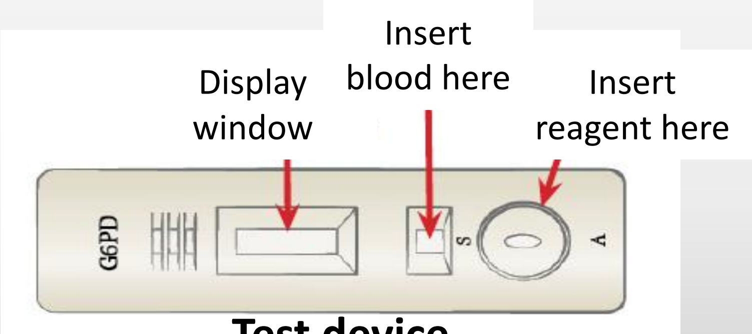

Test device

Pipette

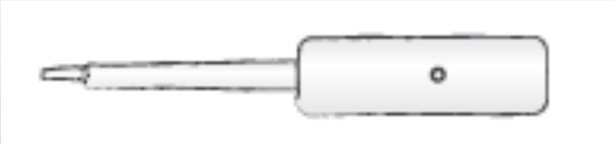

2.5. Write the patient's name on the test device:

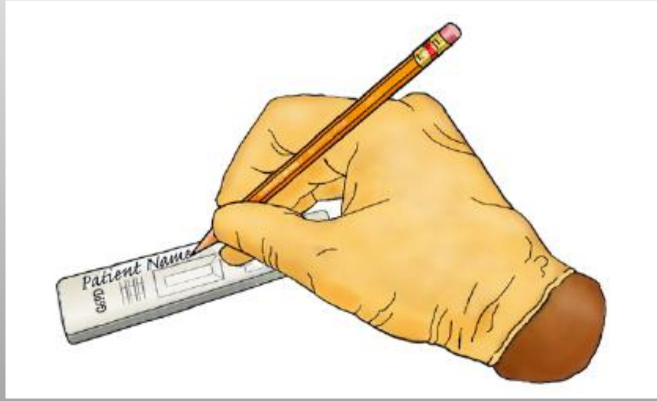

Rapid test device for safe use of primaquine

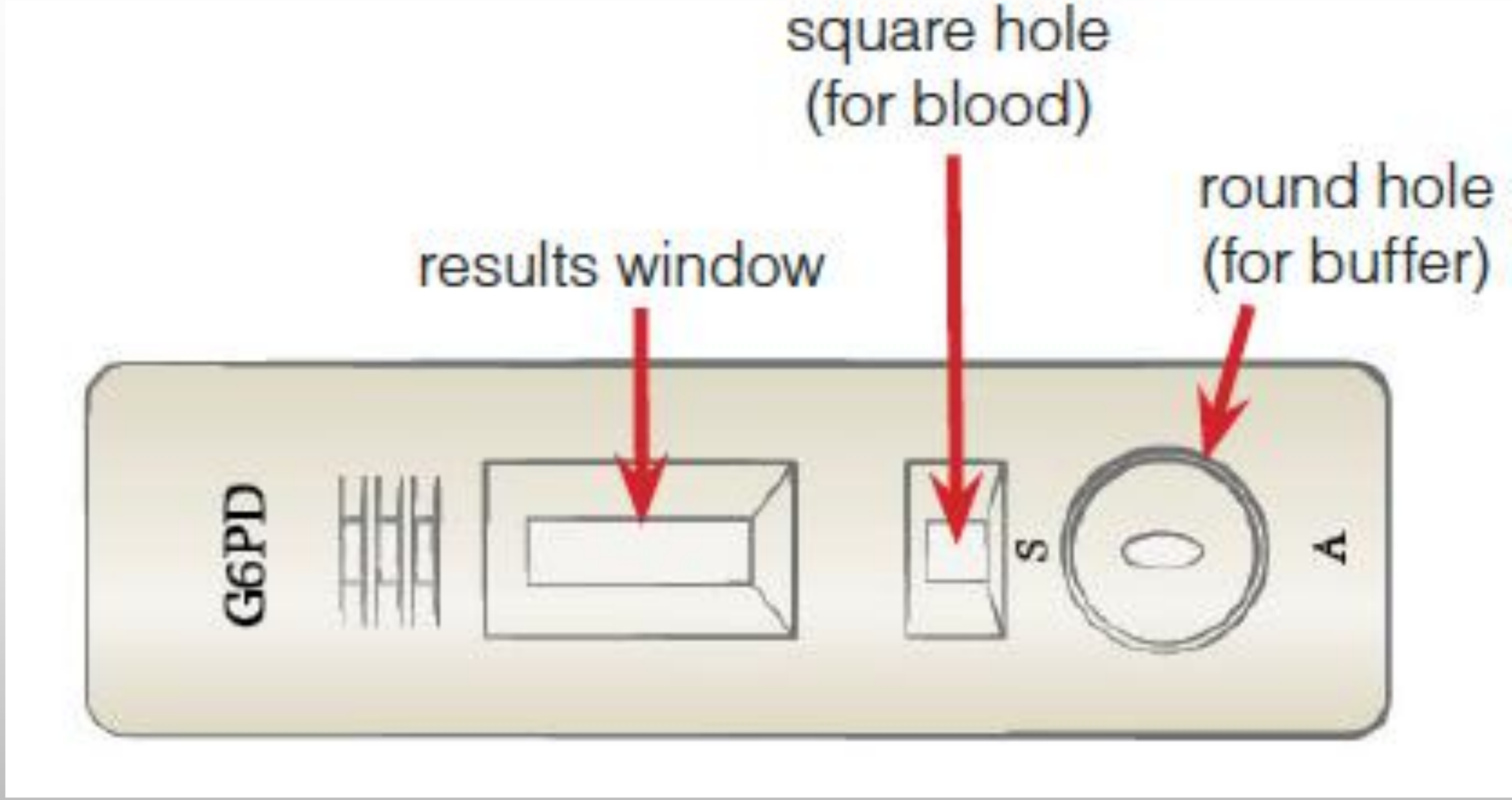

2. How to use the rapid test
# - Preparation for performing the rapid test

2.6. Prepare for the finger prick:

- ✓Observe the patient's fingers and choose the finger tip of the fourth finger
- ✓Perform antisepsis at the finger collection site with the alcohol wipe
- ✓Let the alcohol dry naturally

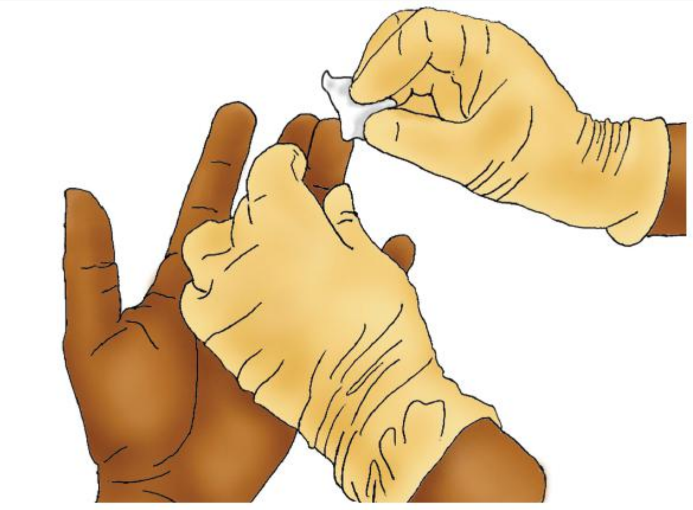

2. How to use the rapid test
# - Sample collection by finger pricking

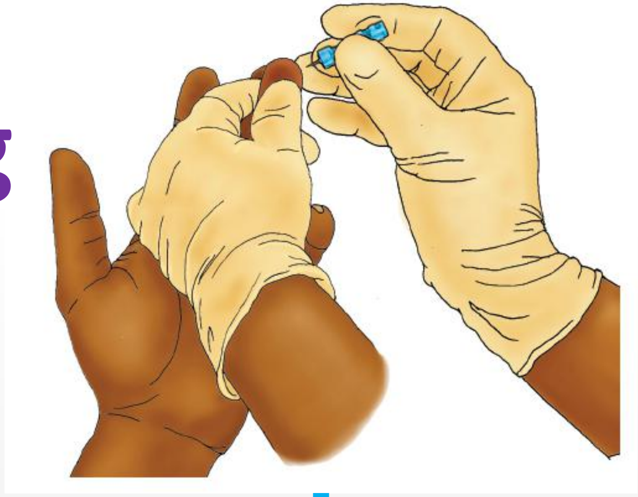

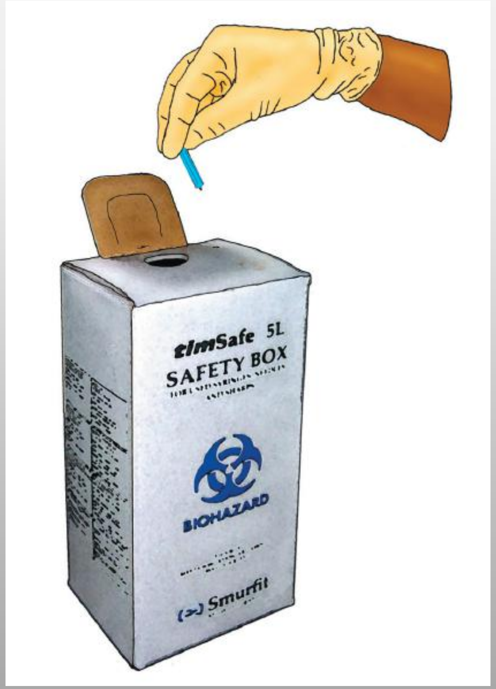

2.7. Perform the finger prick

- ✓ Open the lancet
- ✓ Press the lancet firmly against the finger and pierce the skin on the side of the finger
- ✓ Discard the lancet **immediately** in the sharps disposal box
- ✓ **Wipe the first drop of blood**
- ✓ **Collect the second drop of blood**

2. How to use the rapid test
# - Sample collection by finger pricking

2.8. Collect blood with the pipette

✓Squeeze the patient's finger near the puncture site to form a drop of blood.

✓Collect 2 µL of blood (up to the mark) with the individual pipette.

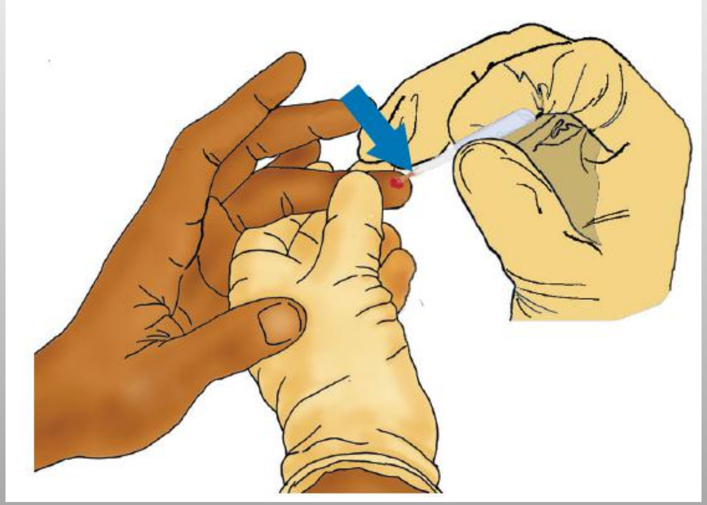

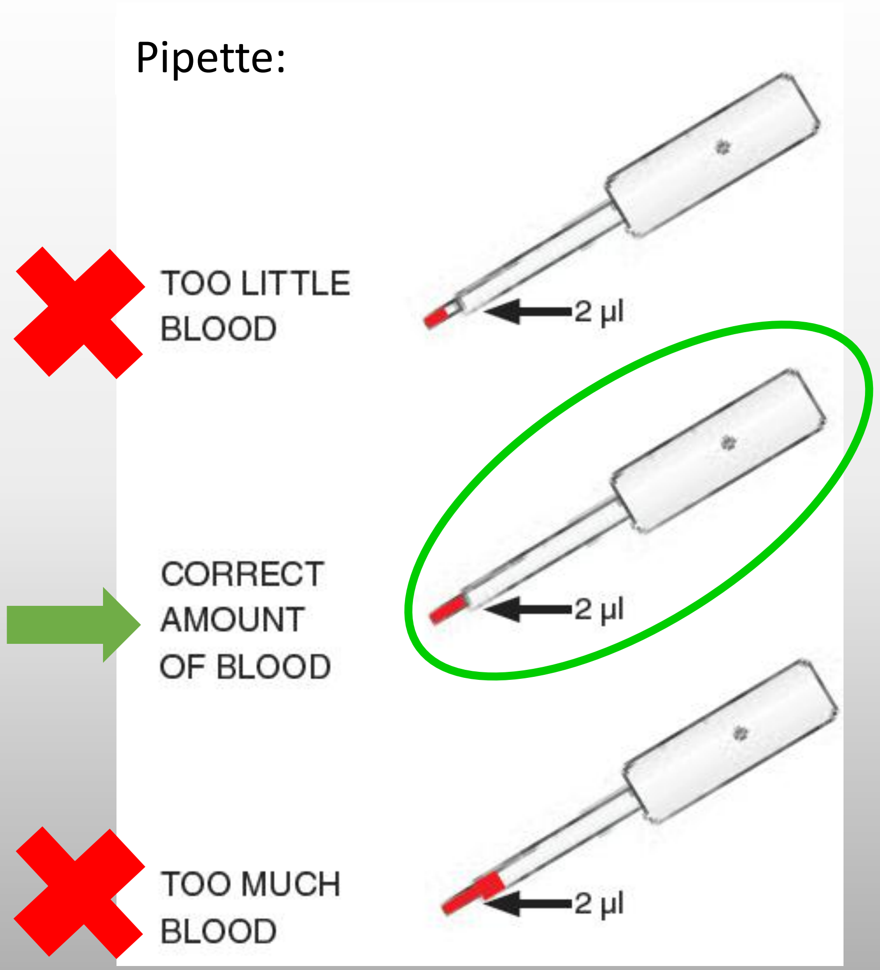

2. How to use the rapid test
# - Performing the test

2.9. Transfer the blood to the test device

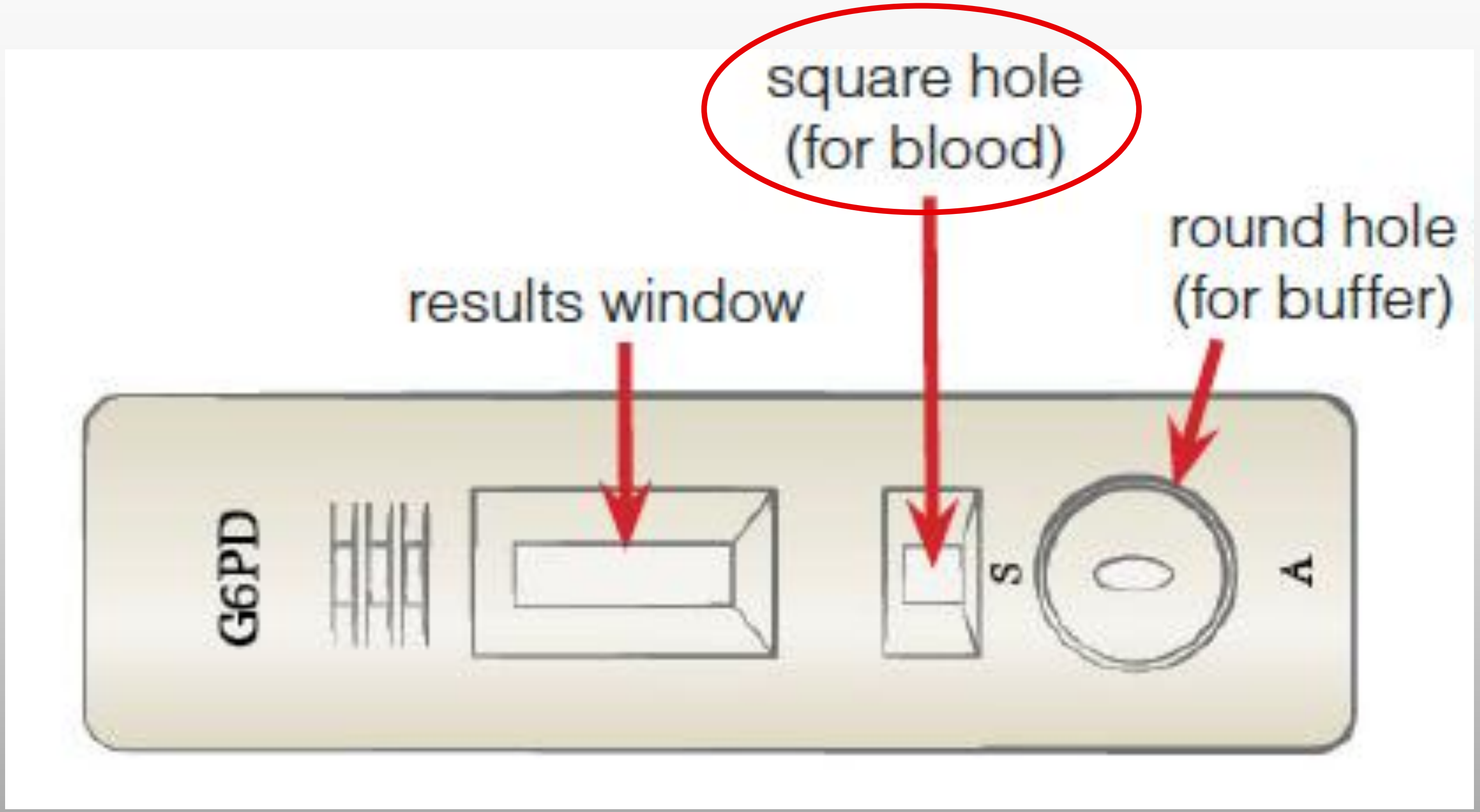

2. How to use the rapid test
# - Performing the test

2.9. Transfer the blood to the test device

✓Place the pipette on the membrane of the SQUARE window for Blood(**Letter ”S”**)

✓Cover and press the hole in the top of the pipette to transfer the blood

✓Check that blood is in contact with the blood window membrane

✓Discard the pipette after use

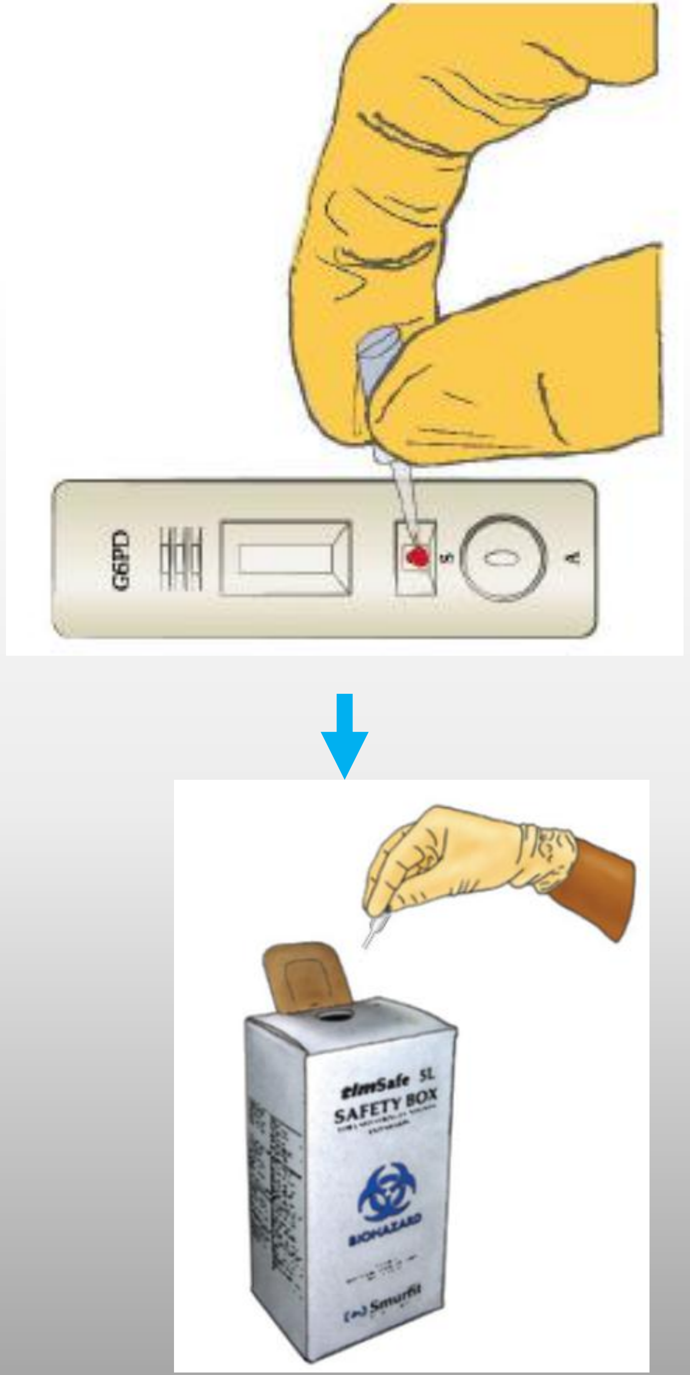

2. How to use the rapid test - Performing the test

2.10. Add the reagent

✓Add **TWO drops** of reagent to the **round window (Letter "A")**

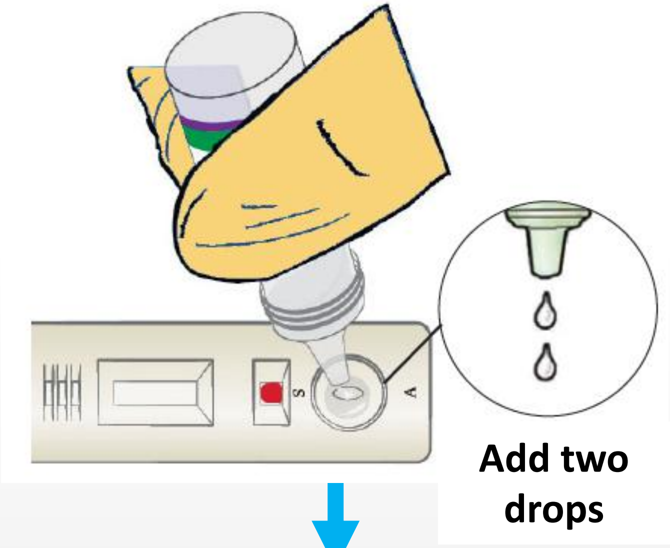

2.11. Wait for the correct time before reading the result

✓Note on the paper the time you added the reagent and the time you should read the test OR measure the time

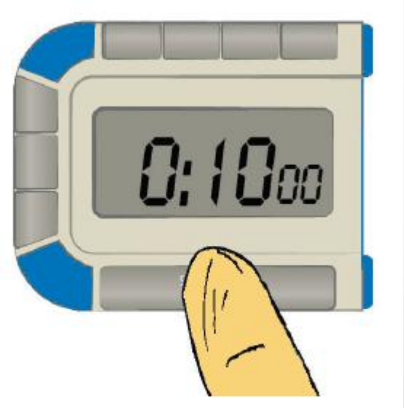

✓**Wait <u>at least 10 minutes</u> and at most 20 minutes**

✓Watch the blood flow to the result window and then disappear

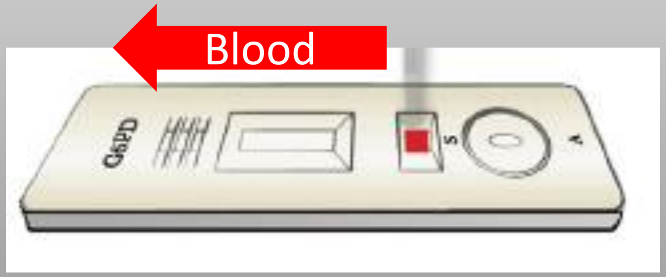

✓Watch the blood flow to the result window and then disappear

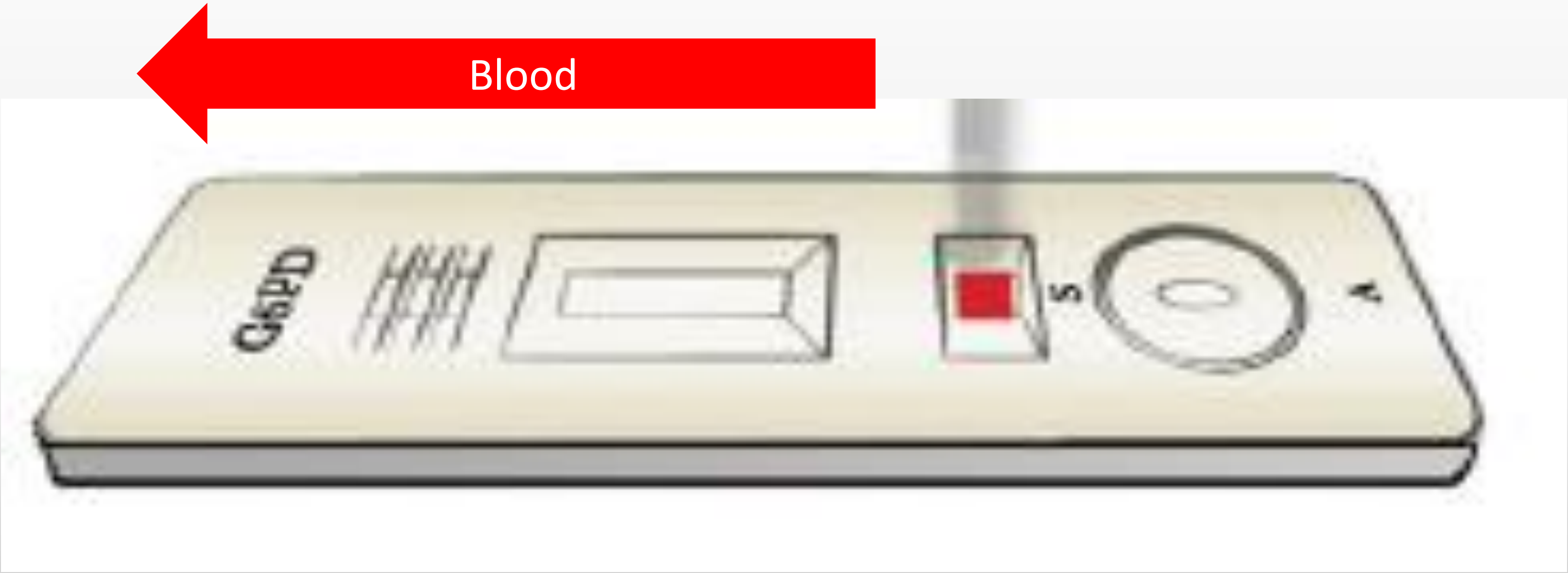

2. How to use the rapid test - Performing the test

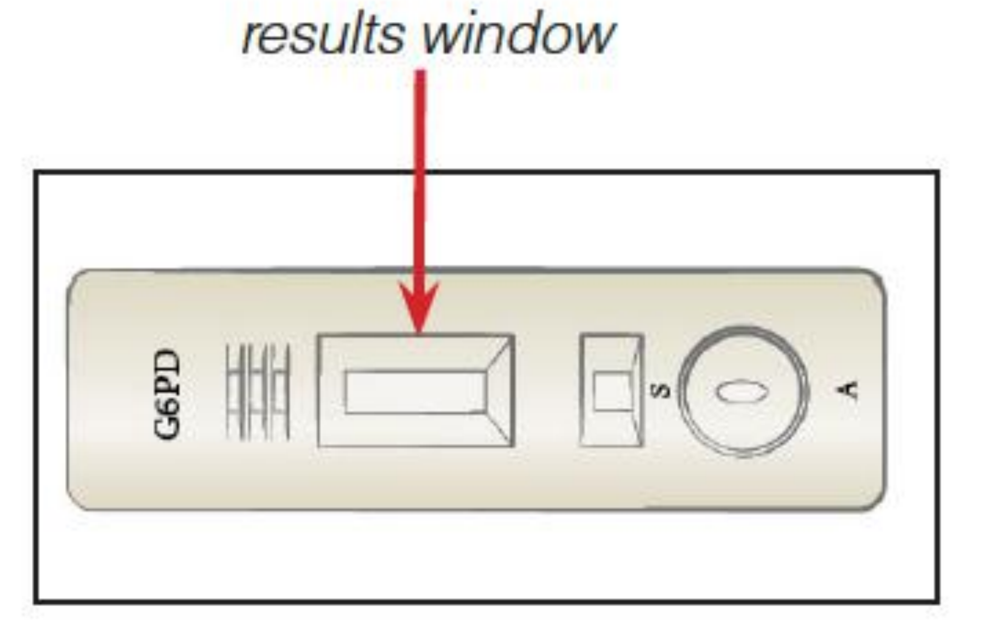

2.12. Read the test result

2.13. Dispose of gloves and test in a contaminated material bin

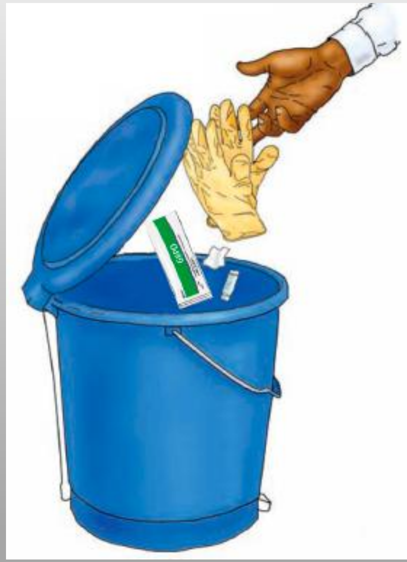

Training video

2. How to use the rapid test

WHAT SHOULD BE REMEMBERED WHEN USING THE RAPID TEST:

- Take proper care when handling blood and disposing of material.
- Follow product guidelines correctly.
- The test should be discarded if the envelope is damaged.
- Open the envelope and use it immediately after opening.
- Tests must not be reused.

2. How to use the rapid test
# - Reading and interpretation of results

➢ POSSIBLE RESULTS

The result is given by the presence of color or its absence.

INVALID TEST

Blood does not flow in the test window, as shown in the following example:

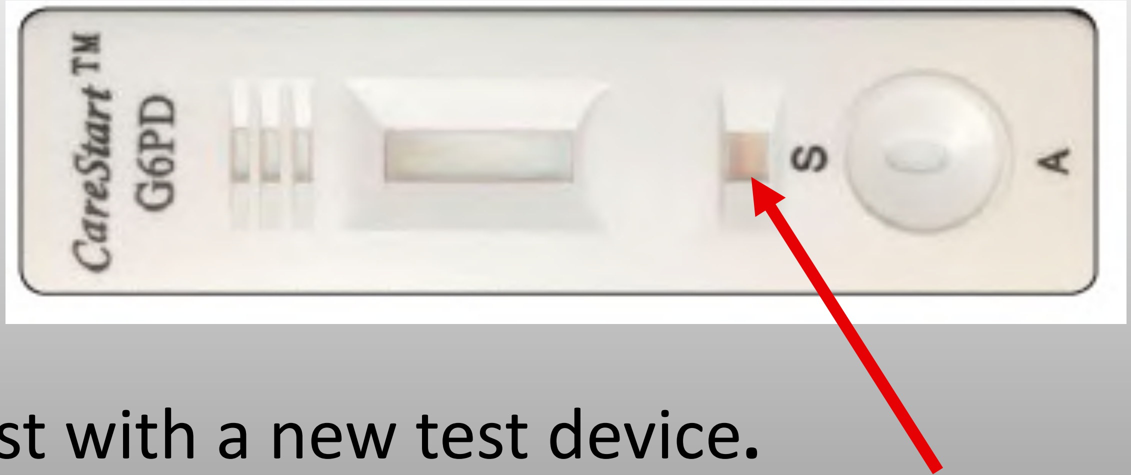

❑ Repeat the test with a new test device.

Reading and interpretation of results

CAN TAKE PRIMAQUINE ON A DAILY BASIS

A **strong purple color** means that the patient **CAN** take Primaquine on a daily basis (7 days):

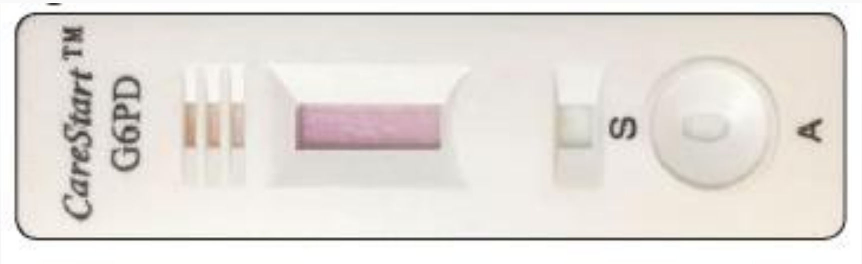

CANNOT TAKE PRIMAQUINE ON A DAILY BASIS

No color change or doubts regarding color (very faint change) means you **CANNOT** use Primaquine on a daily basis:

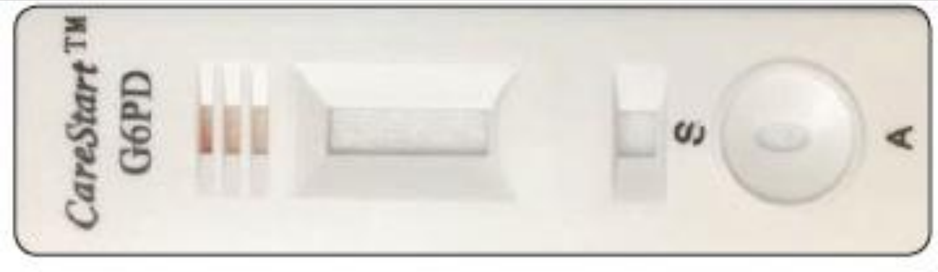

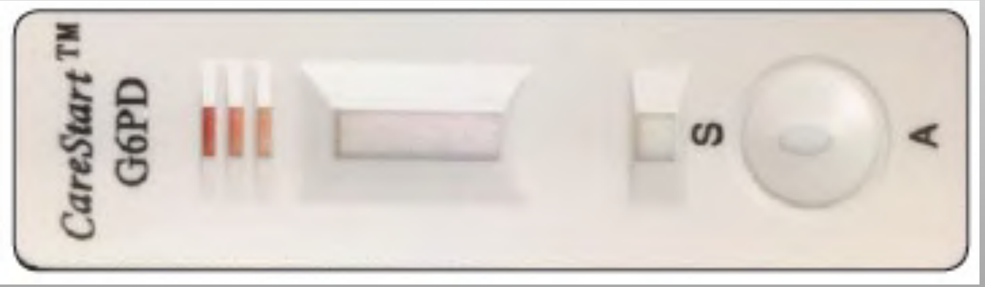

DECISION ALGORITHM

PATIENT WITH *P. VIVAX* MALARIA

PERFORM THE RAPID TEST FOR SAFE USE OF PRIMAQUINE

CAN TAKE PRIMAQUINE ON A DAILY BASIS

UNCOMPLICATED VIVAX MALARIA TREATMENT:
3 days of chloroquine and 7 days of primaquine (0.5mg/kg/day)

CANNOT TAKE PRIMAQUINE ON A DAILY BASIS

VIVAX MALARIA TREATMENT :
3 days of chloroquine with **primaquine weekly for 8 weeks under supervision (0.75mg/kg/week)**

CURE CONFIRMATION SLIDE ON 5th DAY

2. How to use the rapid test
# - Reading and interpretation of results

CAN TAKE PRIMAQUINE ON A DAILY BASIS

Strong purple color means the patient **CAN** use the daily primquine:

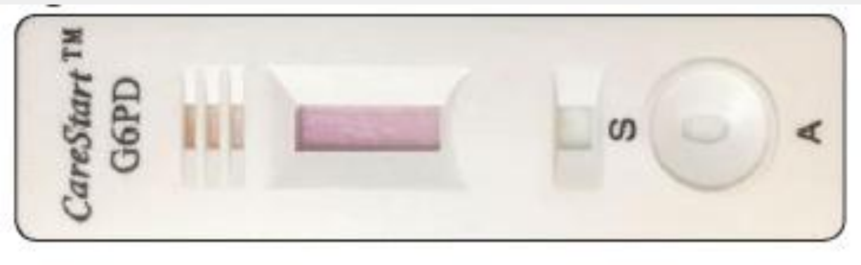

PRIMAQUINE

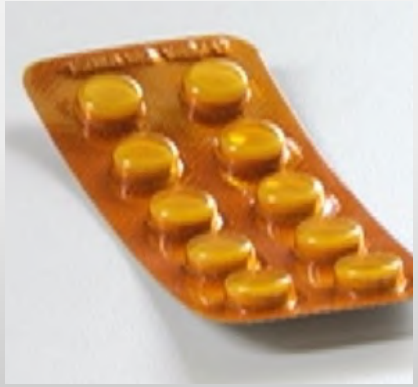

CHLOROQUINE

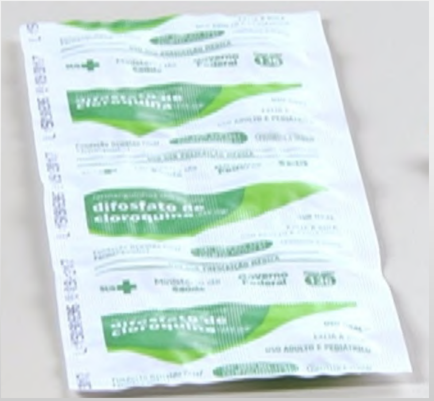

2. How to use the rapid test
# - Reading and interpretation of results

CANNOT TAKE PRIMAQUINE ON A DAILY BASIS

No color change or doubts regarding color (very weak) means that the patient **CANNOT** take primaquine on a daily basis:

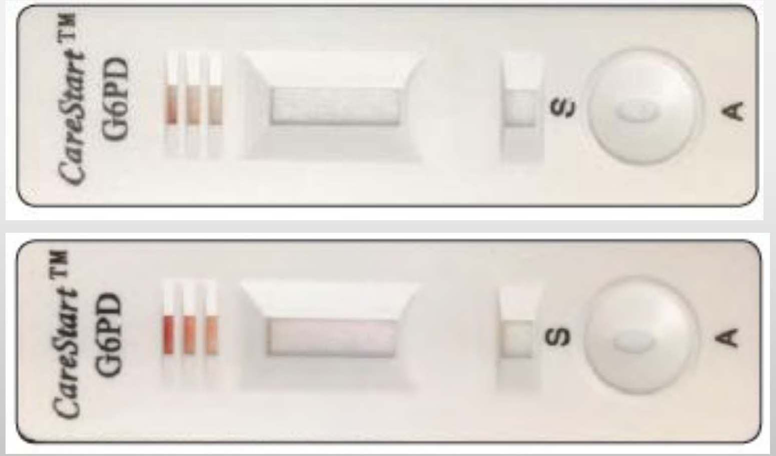

CHLOROQUINE

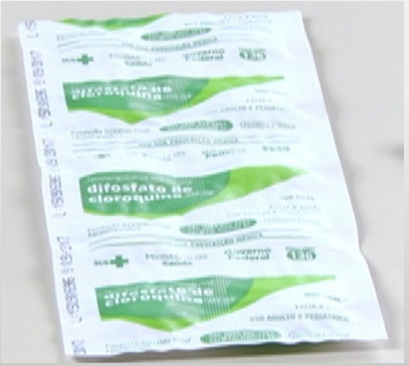

+

WEEKLY SUPERVISED PRIMAQUINE

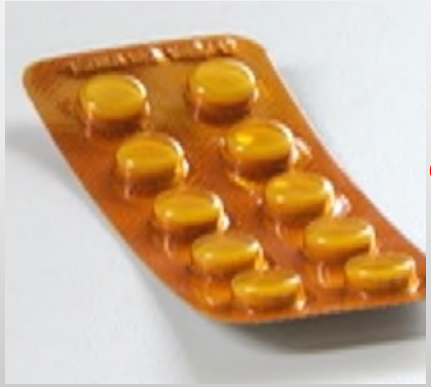

+

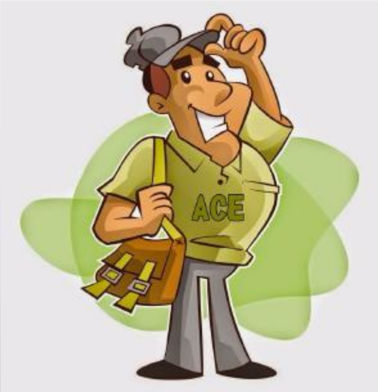

0.75mg/kg/week
1 dose per week
for 8 weeks

E.g. Patient weighs 70kg
75 x 0.75= 56.5mg
56.5/15mg= 3.75 = **4 tabs/15mg for week**
