# Supplementary material for: Real-life implementation of a G6PD deficiency screening qualitative test into routine vivax malaria diagnostic units in the Brazilian Amazon (SAFEPRIM study)
Source: PLoS Negl Trop Dis. 2021 May 18;15(5):e0009415. doi: 10.1371/journal.pntd.0009415 (PMC8162658; doi:10.1371/journal.pntd.0009415)
Supplement: S2 File — A locally recorded video was used for trainings sessions and to be distributed for HCPs during the implementation period. Due to the size of the file, please access it here: https://drive.google.com/file/d/1mm3FyP5S1soZzms1lnYjSJ3q2C_aLWl-/view?usp=sharing. (DOCX) [file pntd.0009415.s002.docx]

**Supplementary file S2. Test procedure for training purposes.** A locally recorded video was used for trainings sessions and to be distributed for HCPs during the implementation period. **Due to the size of the file, please access it here:** <https://drive.google.com/file/d/1mm3FyP5S1soZzms1lnYjSJ3q2C_aLWl-/view?usp=sharing>
