# Supplementary material for: Real-life implementation of a G6PD deficiency screening qualitative test into routine vivax malaria diagnostic units in the Brazilian Amazon (SAFEPRIM study)
Source: PLoS Negl Trop Dis. 2021 May 18;15(5):e0009415. doi: 10.1371/journal.pntd.0009415 (PMC8162658; doi:10.1371/journal.pntd.0009415)

TREATMENT ALGORITHM

The following decision algorithm must be used when employing rapid tests for safe use of Primaquine:

PATIENT HAS *P.VIVAX*

↓

PERFORM THE RAPID TEST FOR SAFE USE OF PRIMAQUINE

↓

PRIMAQUINE CAN BE USED DAILY

↓

TREATMENT OF UNCOMPLICATED *VIVAX* MALARIA: Chloroquine for 3 days and primaquine for 7 days.

PRIMAQUINE CANNOT BE USED DAILY

↓

TREATMENT OF VIVAX MALARIA: Chloroquine for 3 days and weekly primaquine under medical supervision for 8 week.

↓

BLOOD SMEAR ON 5th DAY

PATIENT COUNSELING AFTER TEST

ALL CASES

1. Remind patient of the importance of completing full malaria treatment.
2. Instruct the patient that, whenever he/she has malaria, the doctor must be informed about the result of the rapid test before prescribing Primaquine.
3. Instruct the patient to return on the 5th day of treatment in order to perform a verification blood smear.
4. Stress to the patient that he/she must be attentive to the signs of hemolytic anemia, such as dark urine ("Coca-Cola" color), yellow eyes or skin.
5. At any time, should the patient show signs or symptoms of severity, inform him/her that the specialist at the referral health unit should be sought immediately.

FUNDAÇÃO DE MEDICINA TROPICAL DOUTOR HEITOR VIEIRA DOURADO FMT-HVD

Instituto ELIMINA

ILMD INSTITUTO LEÔNIDAS & MARIA DEANE Fiocruz Amazônia

Fundação de Medicina Tropical Dr. Heitor Vieira Dourado

RAPID TEST FOR SAFE USE OF PRIMAQUINE

WHAT IS THE RAPID TEST FOR?

The rapid diagnostic test for the safe use of primaquine detects people who may experience adverse effects when using primaquine. A common adverse effect is hemolytic anemia.

WHEN IS THE TEST USED?

When a patient is diagnosed with *Plasmodium vivax* malaria.

HOW DOES THE RAPID TEST WORK FOR THE SAFE USE OF PRIMAQUINE?

The rapid test for the safe use of primaquine uses the blood collected from the patient in the test platform with the addition of a reagent. After 10 minutes, the test result is obtained.

STORAGE

- Storage should be on stands or shelves, away from the wall and floor in order to reduce damage by water, moisture and contaminants.
- Protect from the sun, excessive heat, rodents and insects.
- Store in air-conditioned environments or, if there is no air conditioning or refrigeration, ensure a natural ventilation system in the environment, such as windows and vents.
- Do not store in a freezer, respect the manufacturer's recommended storage temperature of between 18 °C and 32 °C.
- Store in an organized manner, keep tests with shorter shelf life the most accessible to users.

TRANSPORT

- Do not leave kits in a vehicle left in the sun.
- If possible, transport should take place in the morning or at night, avoiding high temperatures and exposure to the sun, as well as exposure to rain.
- When transporting on boats, bicycles and motorbikes, avoid exposure to the sun and water.
- Avoid carrying the rapid tests together with food or any material for personal use.

IMPORTANT THINGS TO REMEMBER WHEN USING THE RAPID TEST

- Handle with care any samples, sharps or other contaminatd materials. Dispose properly.
- Follow product guidelines correctly.
- The test kit should be discarded if the envelope is damaged.
- Open the envelope at room temperature only and use it immediately after opening.

HOW TO USE THE RAPID TEST FOR SAFE USE OF PRIMAQUINE

The test is simple, but all guidelines must be strictly followed. The necessary materials for its accomplishment are: rapid test kit, lancet, reagent, pipette, disposable gloves, alcohol wipe, pencil or pen, stopwatch and container for the disposal of sharps.

APPLYING THE RAPID TEST

1. Leave the test at room temperature.
2. Open the envelope.
3. Identify the test device.
4. Clean the patient's fingertip with the alcohol wipe.
5. Prick the patient's fingertip with the lancet.
6. Collect blood sample with individual pipette.
7. Squeeze the pipette bulb to transfer the **blood** to the **square** hole marked with the **letter "S".**
8. Add **2 drops of the reagent** to the **round** hole marked with the **letter "A".**
9. Wait **at least 10 minutes** but **no more than 20 minutes** before checking the result.

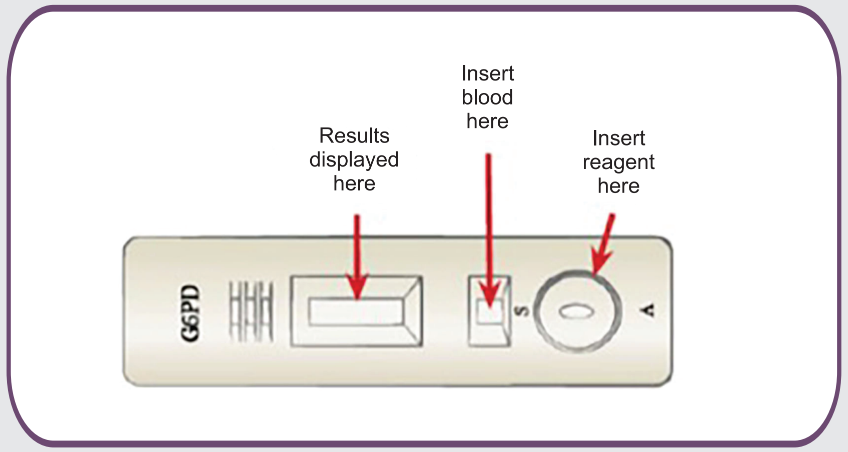

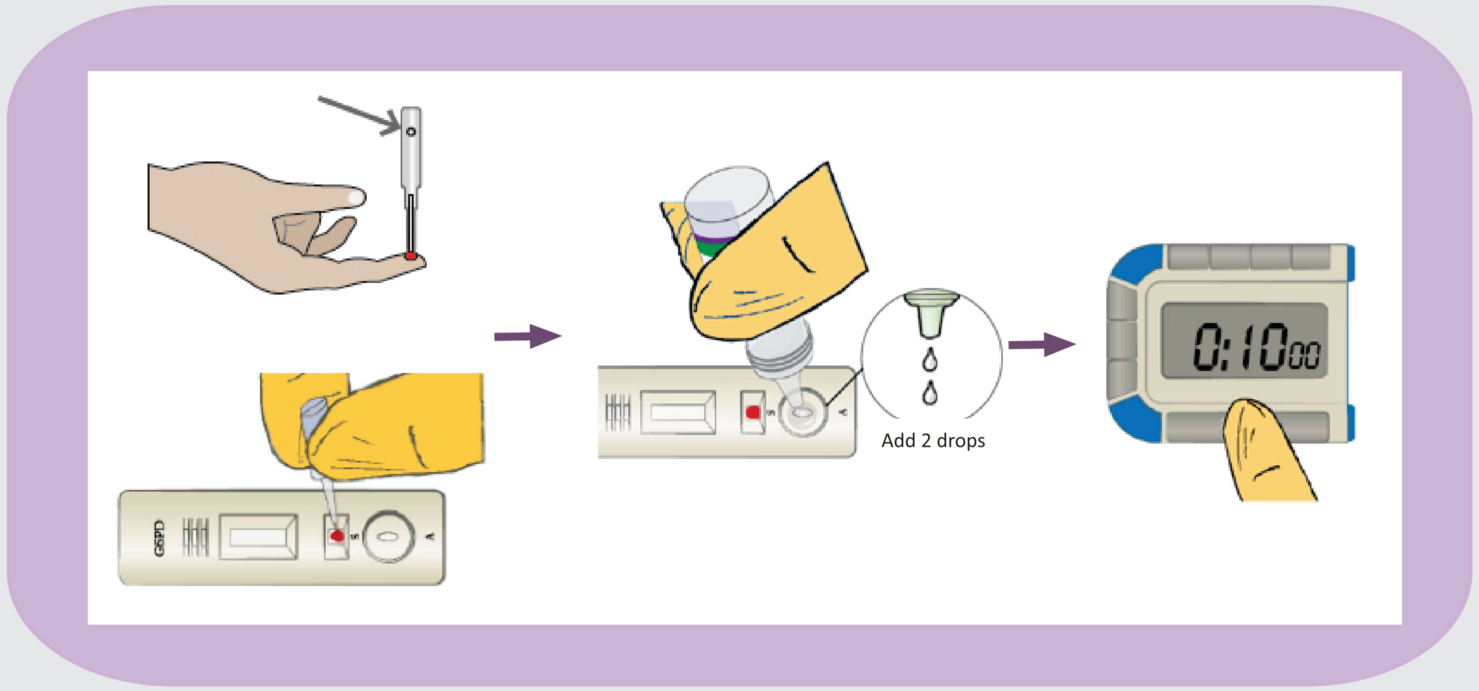

POSSIBLE RESULTS

The rapid test for the safe use of Primaquine is a test which gives its result by presence or absence of color change.

INVALID TEST RESULT

Blood does not flow properly on reading window, as in the example:

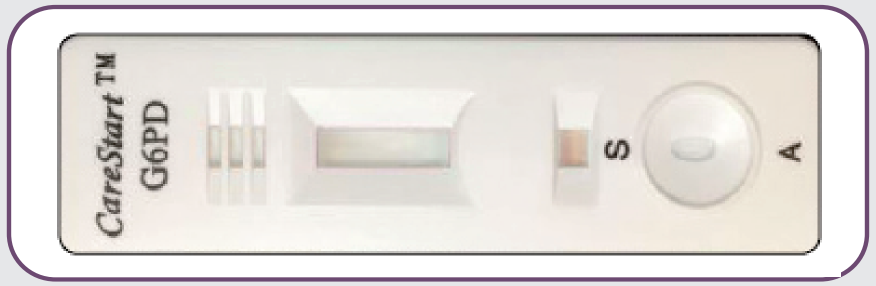

☐ **Repeat the test with a new device.**

PRIMAQUINE CAN BE USED DAILY

Purple color means that the patient **CAN** use daily primaquine.

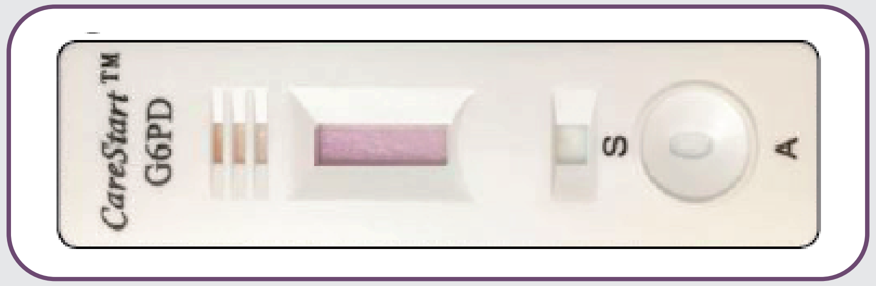

PRIMAQUINE CANNOT BE USED DAILY

No color change or very faint purple color means the patient **CANNOT** use daily primaquine.

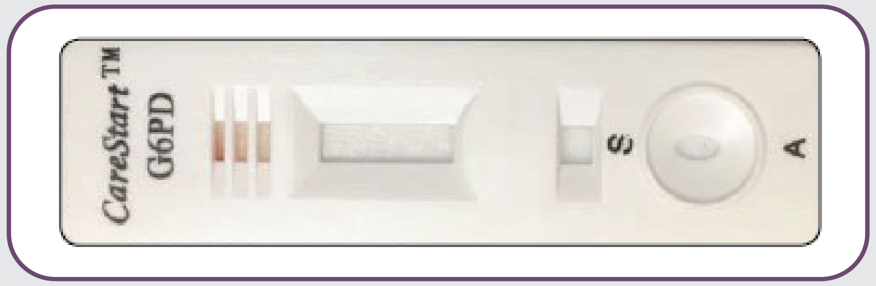

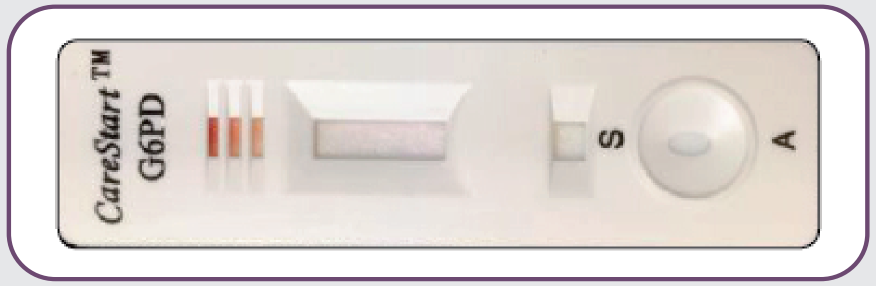

Supplement: S3 File — Folders were provided for every professional during the training sessions (Size when folded: 21.0 x 29.7cm). (PDF) [file pntd.0009415.s003.pdf]
