# Supplementary material for: Real-life implementation of a G6PD deficiency screening qualitative test into routine vivax malaria diagnostic units in the Brazilian Amazon (SAFEPRIM study)
Source: PLoS Negl Trop Dis. 2021 May 18;15(5):e0009415. doi: 10.1371/journal.pntd.0009415 (PMC8162658; doi:10.1371/journal.pntd.0009415)
Supplement: S4 File — Posters were provided for each unit for quick guidance (Size: 29.7 x 42.0cm). (PDF) [file pntd.0009415.s004.pdf]

RAPID TEST FOR SAFE USE OF PRIMAQUINE

INSTRUCTIONS FOR USING THE RAPID TEST:

1 1) Test must be done at room temperature. Open the envelope and clean the patient's finger with alcohol pad.

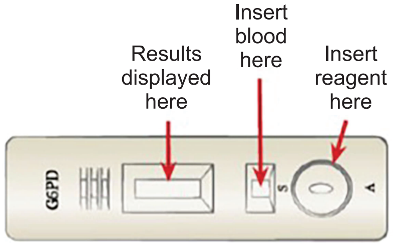

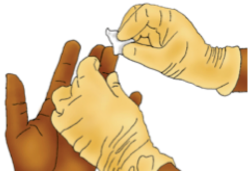

2 2) Prick the fingertip and collect blood with the single use pipette. Squeeze the bulb of pipette to transfer **blood** to the **blood** to the square hole marked witch the **letter 'S'**.

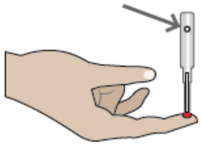

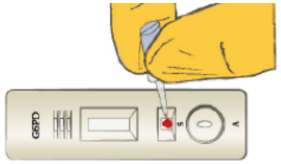

3 3) Add **2 drops of reagent** to the round hole marked with the **letter "A"**. Wait for **<u>at least 10 minutes</u>** and **no more than 20 minutes** in order to read test results.

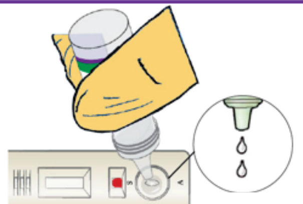

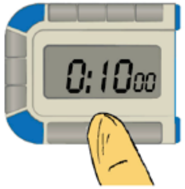

RESULTS:

PRIMAQUINE CAN BE USED DAILY:

A **purple color** indicates that the patient **CAN** use daily primaquine.

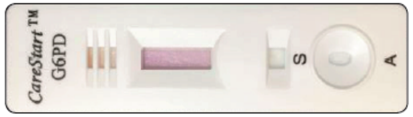

PRIMAQUINE CANNOT BE USED DAILY:

If there is no color change, or if the result is a very faint purple color, the patient **CANNOT** use daily primaquine.

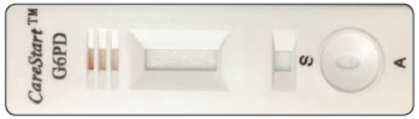

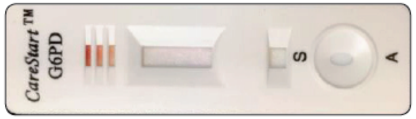

INVALID TEST RESULT

Blood does not flow properly.

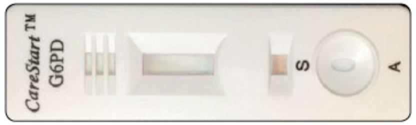

DECISION TREE:

Patient has *P. vivax* infection

↓

Perform rapid test for safe use of Primaquine

↓

**PRIMAQUINE CAN BE USED DAILY**	**PRIMAQUINE CANNOT BE USED DAILY**
↓	↓
**Treatment of uncomplicated vivax malaria: Chloroquine for 3 days and primaquine for 7 days.**	**Treatment of vivax malaria: Chloroquine for 3 days and weekly primaquine under medical supervision for 8 weeks.**

↓

BLOOD SMEAR ON 5th DAY

WHAT TO REMEMBER DURING THE TEST:

- Caution when handing blood and disposing contaminated materials.
- Follow product instructions carefully.
- If the envelope is damaged in any way, the test material should be discarded.
- Do not reuse test devices.

FUNDAÇÃO DE MEDICINA TROPICAL DOUTOR HEITOR VIEIRA DOURADO FMT-HVD

Instituto ELIMINA

ILMD INSTITUTO LEÔNIDAS & MARIA DEANE Fiocruz Amazônia
