# Supplementary material for: Real-life implementation of a G6PD deficiency screening qualitative test into routine vivax malaria diagnostic units in the Brazilian Amazon (SAFEPRIM study)
Source: PLoS Negl Trop Dis. 2021 May 18;15(5):e0009415. doi: 10.1371/journal.pntd.0009415 (PMC8162658; doi:10.1371/journal.pntd.0009415)
Supplement: S5 File — Forms were adapted from the national SIVEP-malaria reporting forms in order to add data on G6PD results and pharmacovigilance of hemolysis. (PDF) [file pntd.0009415.s005.pdf]

Federal Republic of Brazil
Ministry of Health

SIVEP
EPIDEMIOLOGICAL SURVEILLANCE INFORMATION SYSTEM
MALARIA CASE NOTIFICATION

1 Notification No:

NOTIFICATION DATA

2 Notification date:

3 Detection type: 1-Passive 2– Active

4 Symptoms: 1-Symptoms present 2-No symptoms

5 Notifying State:

6 Notifying Municipality:

7 Code of Notifying Municipality:

8 Notifying Health Unit:

9 Code of Notifying Health Unit:

10 Name of Notifying Health Worker:

11 Code of Notifying Health Worker:

PATIENT DATA

12 Patient's name:

13 National Health Card Number:

14 Date of Birth:

15 Age — Age / Month / Year

16 Gender: — Male F- Female

17 Is patient pregnant? 1-1st Trimester 2-2nd Trimester 3-3rd Trimester 4-Gestational age ignored 5-No 6-Does not apply

18 Schooling: 0 – Illiterate 1 – 1st to 4th grade Elementary School Education incomplete 2 – 2nd to 4th grade Elementary School Education complete 3 – 5th to 8th grade Elementary School Education incomplete 4– Elementary School Education complete 5 – High School Education incomplete6 – High School Education complete 7 – Unfinished university degree 8 – Full university degree 10 – Does not apply

19 Race/Color: 1-White 2-Black 3-Yellow 4-Brown 5-Indigenous

20 Mother's name:

21 Principal activity in the last 15 days: 1 – Agriculture 2 – Livestock raising 3 – Domestic duties 4 – Tourism 5 – Mining 6 – Farming 7 – Hunting/Fishing 8 – Construction of dams/highways 9 – Quarrying 10 – Traveling 11 – Others

22 Patient's Address:

23 Other country of residence:

24 State of Residence:

25 Municipality of Residence:

26 Municipality Code:

27) Location of Residence

28 Code of Location of Residence:

29 Date First Symptoms Occurred:

30 Has the patient received treatment for vivax malaria in the last 60 days? 1-Yes 2-No

31 Has the patient received treatment for falciparum malaria in the last 40 days? 1-Yes 2-No

PROBABLE PLACE OF INFECTION

32 Other country where infection could have occurred:

33 State where infection could have occurred:

34 Municipality where infection could have occurred:

35 Municipality code where infection could have occurred:

36) Place where infection could have occurred:

37 Place code where infection could have occurred:

TEST DATA

38 Date of Test:

39 Type of Test: 1-Thick blood smear 2-RDT

40 Test Results: 1- Negative; 2- F; 3- F+FG; 4- V; 5- F+V; 6- V+FG; 7- FG; 8- M; 9- F+M; 10- Ov; 11-No F

41 Parasites por mm^3:

42 Parasitemia counted as "crosses": 1- < +/2 (less than half a cross); 2- +/2 (half a cross); 3 - + (one cross); 4- ++ (two crosses); 5- +++ (three crosses); 6 - ++++ (four crosses)

43 Other Hemoparasites Researched: 1-Negative 2-Trypanosoma sp. 3-Microfilament 4-Trypanosoma sp. + Microfilaria 9-Not Researched

44 Tester's name:

45 Tester's code:

TREATMENT

46 Course of treatment used according to the Malaria Therapy Manual:

1 – P. vivax or P. ovale infections with chloroquine for 3 days and primaquine for 7 days (short course);
2 – P. vivax, or P. ovale infections with chloroquine for 3 days and primaquine for 14 days (long course);
3 – P. malariae infections for all ages and P. vivax or P. ovale infections in pregnant women and children under 6 months of age with chloroquine for 3 days;
4 – Prevention of frequent relapses by P. vivax or P. ovale with weekly chloroquine for 12 weeks;
5 – P. falciparum infections with the fixed combination of artemether + lumefantrine for 3 days;
6 – P. falciparum infections with fixed combination of artesunate + mefloquine for 3 days;
7 – P. falciparum infections with quinine for 3 days, doxycycline for 5 days and primaquine for 6 days;
8 – Mixed infections by P. falciparum and P. vivax or P. ovale with Artemether + Lumefantrina or Artesunate + Mefloquine for 3 days and Primaquine for 7 days;
9 – Uncomplicated P. falciparum infections in the first trimester of pregnancy and in children under 6 months with quinine for 3 days and clindamycin for 5 days;
10 – Severe and complicated P. falciparum malaria in all age groups;
11 – P. falciparum infections with the fixed combination of artemether + lumefantrine for 3 days and single dose primaquine;
12 – P. falciparum infections with the fixed combination of artesunate + mefloquine for 3 days and single dose primaquine;
13 – P. vivax or P. ovale infections with chloroquine for 3 days and weekly primaquine for 8 weeks under medical supervision
99 – Other course used (by doctor) - describe:

47 Treatment Start Date:

RAPID TEST FOR USE OF PRIMAQUINE

48 Rapid test result for use of Primaquine: 1-CAN use primaquine daily a 2-CANNOT use primaquine daily 3-No test done

49 Source of information: 1-RDT 2-Malaria Info Card

50 Has the patient received a blood transfusion in the last 60 days? 1-Yes 2-No

Fill in spaces 51 and 52 only on patient's return on day 5 of treatment

51 Date of last positive malaria test:

52 Has the patient reported/presented dark urine or yellow eyes? 1-Yes 2-No 3-Other - describe:

53 Patient's weight (Kg):

54 Patient's telephone number:

DISTRICT HEALTH DEPARTMENT STATE CODE MUNICIPALITY

12 Patient's name:

15 Age:

1 Notification No:

38 Test Date:

40 Test Results:

44 Tester's name:

48 Rapid test result for the use of Primaquine 1-CAN use primaquine daily a 2-CANNOT use primaquine daily 3-No test done

Proof of exam result to be given to the patient

PATIENT, PLEASE NOTE: If you have **dark urine (coca cola color) or yellow eyes,** seek for medial assistance.
