# Supplementary material for: Real-life implementation of a G6PD deficiency screening qualitative test into routine vivax malaria diagnostic units in the Brazilian Amazon (SAFEPRIM study)
Source: PLoS Negl Trop Dis. 2021 May 18;15(5):e0009415. doi: 10.1371/journal.pntd.0009415 (PMC8162658; doi:10.1371/journal.pntd.0009415)
Supplement: S6 File — A purple card was given to normal and a gray card was given to deficient patients, where the result was recorded and used in future malaria episodes (Size: 8.0x20.0cm). (PDF) [file pntd.0009415.s006.pdf]

Fundação de Medicina Tropical Dr. Heitor Vieira Dourado

FUNDAÇÃO DE MEDICINA TROPICAL DOUTOR HEITOR VIEIRA DOURADO
FMT-HVD

Instituto ELIMINA

ILMD
INSTITUTO LEÔNIDAS & MARIA DEANE
Fiocruz Amazônia

Malaria Info Card

Name:____

Weight (Kg):___________________________________

Date of Birth: _______/_______/_______

Address:______________________________________

____

Mother's name:_________________________________

Phone number:__________________________________

RESULTS OF THE RAPID TEST FOR SAFE USE OF PRIMAQUINE:

This patient has had the G6PD test and **CANNOT** use daily primaquine.

Test date:________/_________/__________
Name of clinic:____________________________________
HCP:____

**Date of malaria test**	**Result**	**Treatment**	**Return date** (Return on day 5 of treatment)	**Signs and symptoms**
_____/_____/_____	☐ *VIVAX*<br>☐ *FALCIPARUM*<br>☐ *MIXED INFEC.*		_____/_____/_____	☐ *Dark urine*<br>☐ *Yellow eyes and or skin*<br>☐ *Others, please list:*
_____/_____/_____	☐ *VIVAX*<br>☐ *FALCIPARUM*<br>☐ *MIXED INFEC.*		_____/_____/_____	☐ *Dark urine*<br>☐ *Yellow eyes and or skin*<br>☐ *Others, please list:*
_____/_____/_____	☐ *VIVAX*<br>☐ *FALCIPARUM*<br>☐ *MIXED INFEC.*		_____/_____/_____	☐ *Dark urine*<br>☐ *Yellow eyes and or skin*<br>☐ *Others, please list:*
_____/_____/_____	☐ *VIVAX*<br>☐ *FALCIPARUM*<br>☐ *MIXED INFEC.*		_____/_____/_____	☐ *Dark urine*<br>☐ *Yellow eyes and or skin*<br>☐ *Others, please list:*

- If your **urine becomes dark, similar to Coca Cola**, **your eyes or skin become yellow**, seek medical attention.
- **Do not stop taking your medicine**, even if you are feeling better.
- Always **show this card to the nurse or your doctor**, if you are suspected of having malaria.

FUNDAÇÃO DE MEDICINA TROPICAL
FMT-HVD
DOUTOR HEITOR VIEIRA DOURADO

Instituto
ELIMINA

ILMD
INSTITUTO LEÔNIDAS
& MARIA DEANE
Fiocruz Amazônia

Fundação de Medicina Tropical Dr. Heitor Vieira Dourado

Malaria Info Card

Name:____

Weight (Kg):_______________________________________

Date of Birth: _______/_______/_______

Address:____

____

Mother's name:_____________________________________

Phone number:_____________________________________

RESULTS OF THE RAPID TEST FOR SAFE USE OF PRIMAQUINE

Test date:________/_________/__________

Name of clinic:____________________________________

HCP:____

This patient has had the G6PD test and **CAN** use daily primaquine.

Date of malaria test	Result	Treatment	Return date (Return on day 5 of treatment)	Signs and symptoms
_____/_____/_____	☐ *VIVAX*<br>☐ *FALCIPARUM*<br>☐ *MIXED INFEC.*		_____/_____/_____	☐ *Dark urine*<br>☐ *Yellow eyes and or skin*<br>☐ *Others, please list:*
_____/_____/_____	☐ *VIVAX*<br>☐ *FALCIPARUM*<br>☐ *MIXED INFEC.*		_____/_____/_____	☐ *Dark urine*<br>☐ *Yellow eyes and or skin*<br>☐ *Others, please list:*
_____/_____/_____	☐ *VIVAX*<br>☐ *FALCIPARUM*<br>☐ *MIXED INFEC.*		_____/_____/_____	☐ *Dark urine*<br>☐ *Yellow eyes and or skin*<br>☐ *Others, please list:*
_____/_____/_____	☐ *VIVAX*<br>☐ *FALCIPARUM*<br>☐ *MIXED INFEC.*		_____/_____/_____	☐ *Dark urine*<br>☐ *Yellow eyes and or skin*<br>☐ *Others, please list:*

- If your **urine becomes dark, similar to Coca Cola**, **your eyes or skin become yellow**, seek medical attention.
- **Do not stop taking your medicine**, even if you are feeling better.
- Always **show this card to the nurse or your doctor**, if you are suspected of having malaria.
