# Supplementary material for: Real-life implementation of a G6PD deficiency screening qualitative test into routine vivax malaria diagnostic units in the Brazilian Amazon (SAFEPRIM study)
Source: PLoS Negl Trop Dis. 2021 May 18;15(5):e0009415. doi: 10.1371/journal.pntd.0009415 (PMC8162658; doi:10.1371/journal.pntd.0009415)
Supplement: S7 File — (PDF) [file pntd.0009415.s007.pdf]

FUNDAÇÃO DE MEDICINA TROPICAL – DOUTOR HEITOR VIEIRA DOURADO

INSTITUTO DE PESQUISA CLÍNICA – CARLOS BORBOREMA

FIELD TEAM:
ALÍCIA CACAU
LEONARDO LINCOLN
TALITA BASTOS

COORDINATOR: DR FELIPE MURTA

INTERVIEW SCRIPTS FOR PROFESSIONALS AND PATIENTS IN THE STUDY: IMPLEMENTATION OF THE RAPID TEST FOR THE USE OF PRIMAQUINE IN MALARIA DIAGNOSTIC UNITS IN TWO MUNICIPALITIES IN THE BRAZILIAN AMAZON REGION

MANAUS – AM

1.0 INTERVIEW SCRIPT: PRE-TRAINING OF HEALTH PROFESSIONALS

START RECORDING (RECORD PARTICIPANT'S NAME)

INTRODUCTION AND OBJECTIVES

Good Morning/Good afternoon, my name is [specify name] of [specify organization]. The Fundação de Medicina Tropical Dr. Heitor Vieira Dourado (FMT – HVD) is carrying out a study in order to understand the perception of health professionals regarding the application of the rapid diagnostic test for the use of primaquine (RTP). This assessment will help us to understand how malaria treatment can be improved. We would like to request that you be part of an interview about the implementation of the RTP.

Thank you [for agreeing to participate] or [for your time].

I) INTRODUCTION

1) Please, could you talk a little about your specific role and responsibilities in your work? **[QUESTION THE FOLLOWING, IF IT WAS NOT MENTIONED]**
    a) When did you start this job?
    b) How long have you been working in the field with malaria?
    c) What were you doing before this job?

II) MALARIA AND THE IMPORTANCE OF VIVAX MALARIA

1) Do you take care of many patients with malaria?
2) What type of malaria do you treat in the region?
3) Do you consider vivax malaria a problem in the region? Why?
4) Do you think that malaria financially/economically affects the people and people of the families who are infected? How so?
    a) Does it affect school attendance and/or performance in children?
    b) Does it cause loss of wages or job loss?

III) DIAGNOSIS OF MALARIA AND RAPID TEST

1) How is the diagnosis of malaria carried out?
2) Have you ever used the rapid test for malaria?
   a) How was your experience with the rapid test?
3) What is a rapid diagnostic test?
4) Do you trust the result of the rapid test?
   a) If not, why?

IV) IMPLEMENTATION OF THE RAPID TEST FOR THE USE OF PRIMAQUINE

1) Have you heard of the rapid test for the use of primaquine?
   a) If yes, what do you know about the test?

V) TREATMENT OF VIVAX MALARIA

1) How is malaria treatment currently performed?
2) What treatment is given for vivax malaria?
   a) And for pregnant women or patients under 6 months of age?
   b) And for severe malaria (very high fever, seizures, shortness of breath, anemia and severe vomiting)?
3) What are the challenges for malaria treatment in your region?
4) Are there many cases of treatment abandonment in your region?
5) In your opinion, what would be the reasons for abandoning treatment?
6) Do patients return to perform the cure check slide to see if they have been cured? How often?

VI) TREATMENT WITH PRIMAQUINE

1) Do you know if there are problems associated with the treatment of vivax malaria?
   a) If so, what are the associated problems?
   b) Do you think that they are related to primaquine?
2) Do you know of any cases where a patient had a complication due to the use of the vivax malaria medicine?
   a) Do you think was related to primaquine?
3) Do you know of any cases where a patient presented dark urine (Coca-Cola color) or yellow eye/skin?

   a) Do you think was related to primaquine?
4) Do you know of any case of a patient who had to have blood transfusion because of vivax malaria treatment?
   a) Do you think it was related to primaquine?

VII) PHARMACOVIGILANCE

1) What is the measure taken when a patient has any adverse effect/unexpected effect or complication due to the treatment of malaria?
2) In case of adverse effect caused by the medicinal product, is it reported to someone or a specific department?

VIII) CLOSURE

1) That is the end of my questions. Thank you very much for your time and participation. Do you have any questions for me?

**1. Notifying unit:**	**Sivep Code:**
**2. Municipality:**	
**3. Date:____/____/_____**	
**4. Name of Observer:**	
**5. Professional**: ( ) 1-Physician; 2-Nurse; 3-Nursing Assistant; 4-Nursing Technician; 5-Community Health Worker, 6-Malaria Control Worker, 7-Microscopist; 8-specify: ______________________________________	
**6. Years of service:** ___________ years	
**7. Education level:** ( ) 1-Primary education complete; 2-Primary education incomplete; 3-Secondary education complete; 4-Secondary education incomplete; 5-Higher learning incomplete; 6- Higher learning complete; 7-illiterate;8- Declined to inform	
**8.** Interview began at: _____:_____	Interview ended at: _____:_____
**9.** Recording number:	

2.0 INTERVIEW SCRIPT AND FOCUS GROUP: POST-TRAINING OF HEALTH PROFESSIONALS

START RECORDING (RECORD PARTICIPANT'S NAME)

INTRODUCTION AND OBJECTIVES

Good Morning/Good afternoon, my name is [specify name] of [specify organization]. The Fundação de Medicina Tropical Dr. Heitor Vieira Dourado (FMT – HVD) is carrying out a study in order to understand the perception of health professionals regarding the application of the rapid diagnostic test for the use of primaquine (RTP). This assessment will help us to understand how malaria treatment can be improved. We would like to request that you be part of an interview about the implementation of the RTP.

Thank you [for agreeing to participate] or [for your time]

I) INTRODUCTION

1) Please, could you talk a little about your specific role and responsibilities in your work? **[QUESTION THE FOLLOWING, IF IT WAS NOT MENTIONED]**
   a) When did you start this job?
   b) How long have you been working in the field with malaria?
   c) What were you doing before this job?

II) MALARIA AND THE IMPORTANCE OF VIVAX MALARIA

1) Do you take care of many patients with malaria?
2) What type of malaria do you treat in the region?
3) Do you consider vivax malaria a problem in the region? Why?
4) Do you think that malaria financially/economically affects the people and people of the families who are infected? How so?
   a) Does it affect school attendance and/or performance in children?
   b) Does it cause loss of wages or job loss?

III) ANALYSIS OF TRAINING

1) How was the training given by our team?

IV) Positive points in relation to training? Why?

V) Negative points in relation to training? Why?

VI) If I gave you a test now would you feel confident using it?

VII) Do you think there is a need for further training? Why?

VIII) Was the content confusing?

IX) Were the classes tiring? To what extent could the training become more interesting?

X) Did teachers know how to pass on the information clearly?

XI) Did you end the class with any questions about the test?

1) What is G6PD?
2) Do you think it's necessary to test the patient for G6PD deficiency?
   a) If not, why?

XII) IMPLEMENTATION OF THE RAPID TEST FOR THE USE OF PRIMAQUINE

1) Patient refuses to take the rapid G6PD Test. (Why does he refuse?)
2) Test is done after the result of drop test. (Is that a problem?)

XIII) Distribution Logistics of the medication

1) Do you think the patient understands the reason for performing the G6PD Test? Regarding malaria, what is the role of the Community Health Worker (CHW)? What is your function?

XIV) Do you think the CHW can perform the G6PD Test on the patient? Do you agree that it is or is not the function of CHW to test patients?

XV) Since it is the Malaria Control Worker (MCW) is who collects the slide, is there any problem regarding the performance of the test being done by the CHW? Why?

XVI) Does the test interfere with your normal routine? Why?

1) The Microscopist is the person who is responsible for the storage and distribution of tests.

XVII) Does this cause a problem for the microscopist? Does it interfere with the microscopist's routine?

XVIII) Is that a good strategy? Will there be any difficulty in accessing the tests?

XIX) How are the tests being stored?

XX) Can the tests stay with the agents in the field? What will they do with the buffer solution?

XXI) EXECUTION OF RAPID TEST AND RELIABILITY

XXII) Do you trust the result of the rapid test? Why? Any problems?

XXIII) Is this test better to do in the field or in the laboratory? Why?

XXIV) Are there any difficulties in interpreting the test?

XXV) Is viewing the test result difficult? Why/How so?

XXVI) How would you improve visualization with the existing test?

XXVII) Do you have any difficulty in performing the test? Examples?

XXVIII) Have you had a case of a patient with *vivax* that hasn't been tested? Why?

XXIX) Diagnosis of *P. falciparum* malaria, is testing done or not? Why/Why not?

XXX) Diagnosis of *P. vivax* malaria, is testing done or not? Why/Why not?

XXXI) Diagnosis of mixed malaria, is testing done or not? Why/Why not?

XXXII) Test image. Where to put the sample and where to put the buffer solution? [USE IMAGES]

Evaluate the images and give the result. Did you find any difficulty? [USE IMAGES]

XXXIII) COMPLETING THE SIVEP FORM

1) What do you think of the SIVEP form?
2) What did you think of the change in the form? Does it disrupt your routine? Why?
3) Do you consider the following question important: "Have you had vivax malaria in the last 60 days?" Why?
4) Sometimes SIVEP forms that are filled in incompletely. Why do you think this happens? Give examples of situations in which this can occur.

XXXIV) CARD FOR THE USE OF PRIMAQUINE

1) What did you think of the card?
2) What do you think of the term G6PD deficiency? Does it get the patient's attention?
3) What do you think of the term “apt to receive primaquine”?
4) Did the patient receive the card well? Was it hard to explain?

5) What about the color of the card?
6) Do you think it's feasible to give the patient that card?
7) The patient was tested 4 months ago and contracted malaria again. He/she has the purple card. Should he/she test again for G6PD?
8) The patient was tested 4 months ago and contracted malaria again. He/she has the gray card. Should he/she test again for G6PD?
9) If the patient does not have the card and claims that he/she took the test and that the color of the card was purple (can use primaquine), does he/she have to take the test again? Why?
10) Is there a copy of the card given to the patient or a photo that is in the health unit? Is it registered in a notebook?

XXXV) ACTIVE SEARCH (AS) AND G6PD TESTING

XXXVI) What difficulties in doing AS? Patient weighing?

XXXVII) What is the difficulty in doing the AS + G6PD Test?

XXXVIII) What can be improved in regards to the test to be used in the field?

XXXIX) Is the waiting time to get the test result a problem? Why?

XL) CURE CHECK SLIDE (CCS IS A THICK BLOOD SMEAR ON 5th) AND ITS IMPORTANCE

XLI) What is the difficulty involved in doing a CCS on the 5th day?

XLII) What is the importance of the CCS?

XLIII) Why doesn't the patient return?

XLIV) What are the main factors?

XLV) What can be done to increase the number of CCS done?

XLVI) TREATMENT SCHEME

XLVII) A pregnant patient is diagnosed with vivax malaria: is a G6PD test done or not? If the test result is purple, what would be the treatment scheme? What if the result is gray?

XLVIII) A woman with vivax malária, who is breastfeeding: is a G6PD test done or not? If the test result is purple, what treatment scheme? What if the result is gray?

XLIX) Patient has vivax malaria, the test result is gray. Is he/she treated with primaquine? For how many days?

L) What do you do with the patient who got a gray card?

LI) What did you think of the new treatment scheme of primaquine weekly for 8 weeks?

LII) In relation to G6PD patients, how did changing treatment from 1 week to 8 weeks impact their lives?

XI) HEMOLYSIS

[Use pictures of real cases for demonstration purposes]

a) What is the color of the urine of a patient with hemolysis?
b) The patient reported dark urine, we did the first test and it was negative. Do you think we can test again?
c) The patient has malaria and gray G6PD test result, but reported dark urine during treatment. What do you think might have happened? Why?
d) If a patient has dark urine what guidance would you give him/her? In your opinion, would yellowish skin indicate hemolysis?

TESTING OF EDUCATIONAL MATERIALS

The focus of this section is to evaluate the different visual and message elements of the instructional materials, comparing the character-based and Icon-based versions. The interviewer shall give the participant(s) **RED** and **GREEN** markers and instruct them to return to the version of the LEAFLET issued for the previous activity. Circle in **RED** anything that the parent/guardian considers confusing and/or unpleasant for any reason and b) Circle in **GREEN** anything that the Parent/Guardian finds clear, easy to understand/follow and/or pleasant for any reason.

[For **GREEN** circled areas, ask:]

1. In what ways is it easy to understand/pleasant?
2. What specifically do you like in this part? Why?
3. How can this be improved, how can it be more clear/attractive to you?

CARD

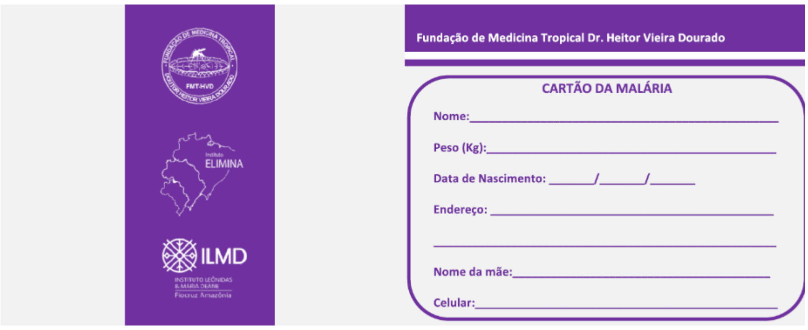

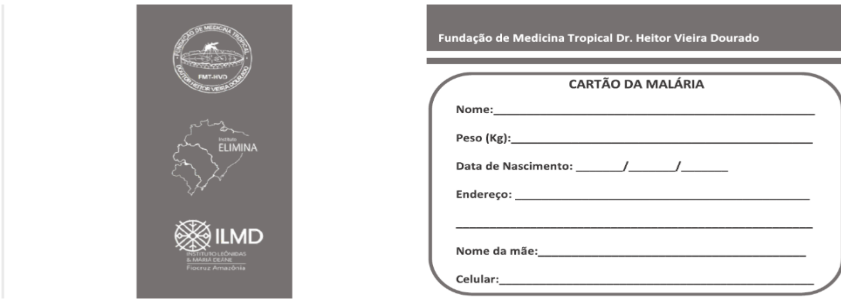

FOLDER

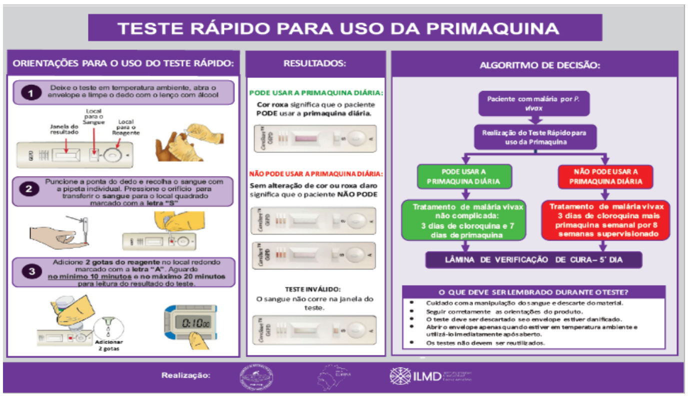

LIII) CLOSURE

Those were all the questions I had for you. Thank you very much for your time and participation. Do you have any questions for me?

3.0 INTERVIEW SCRIPT FOR PATIENTS: POST-IMPLEMENTATION

START RECORDING (RECORD PARTICIPANT'S NAME)

INTRODUCTION AND OBJECTIVES

Good Morning/Good afternoon, my name is [specify name] of [specify organization]. The Fundação de Medicina Tropical Dr. Heitor Vieira Dourado (FMT – HVD) is carrying out a study in order to understand the perception of health professionals regarding the application of the rapid diagnostic test for the use of primaquine (RTP). This assessment will help us to understand how malaria treatment can be improved. We would like to request that you be part of an interview about the implementation of the RTP.

Thank you [for agreeing to participate] or [for your time].

1. **Thematic axis: Experiences**
    1) Do you have any interesting experiences/stories about malaria?
    2) Who was associated with the story?
    3) Is this person a family member?
    4) Where did it happen?
    5) When did it happen?
    6) How did the story end?

2. **Thematic axis: current context of malaria and control measures**
    7) How do you view the current malaria situation in your neighborhood/city?
    8) Does anyone in your family currently have malaria?
    9) What do you feel when you think about malaria?
    10) Is there any way to prevent malaria?
    11) How do we catch malaria?
    12) How do you protect yourself from malaria?
    13) What time of day do you think people catch malaria? (morning, afternoon or evening)? Why?

14) Is the place of residence related to malaria? Why?

3. **Thematic axis: diagnosis and treatment**

15) How is malaria detected in the person (which examination is done)?
16) Have you ever had malaria tested before? Where was it done?
17) Do you think the test for malaria is reliable? Why/why not?
18) What do you think of the medicine for malaria?
19) What about medical care?
20) Do you receive visits from the Family Health Program?
21) How do you rate health care in your neighborhood or near your home?

I) IMPLEMENTATION OF THE RAPID TEST FOR THE USE OF PRIMAQUINE

1) The health workers did a test that was different from the usual malaria test and then you got a card, correct?

- What explanation did you get about the test?
- Do you think this test is important?
- At some point did they mention about the acronym G6PD?
- [G6PD DEFICIENT ONLY] - what did you think of taking primaquine weekly? What are the problems? Did you feel uncomfortable having a different treatment?
- What does it mean to be apt to receive primaquine and inapt?
- How was your test done? Did you find it a problem to test malaria first and then G6PD? Why?
- What can be improved so that people with malaria accept G6PD testing? Why?
- Do you trust the result of the rapid test? Why?

II) CARD FOR THE USE OF PRIMAQUINE

1) What did you think of the card?
2) What do you think of the term G6PD deficiency? Would it be better to use APT or INAPT? Why?

3) Do you think it's feasible to always present this card when you have symptoms of malaria?
4) Where do you keep this card? Can you show me the place? (PHOTO)
5) Do you have cell phone? Does it have internet access?
6) Do you use Whatsapp or text message more often?
7) If you lose the card, do you think it necessary to do a new G6PD Test? Why?
8) What would you so you do not lose the card? And how would you remember the color of the card?

III) TREATMENT

- Do you trust the malaria medicine they give you?
- When you had malaria, did you have any difficulty taking the medicine? Which one?
- Did you have difficulty going to the health unit? Why?

IV) THE CCS AND ITS IMPORTANCE

- After taking all the medicines do you think you need to go to the health unit? Why?
- After how many days should you return to the health facility? Why?
- Do you think the same malaria can come back? Why?

V) HEMOLYSIS

- Did the health worker mention anything about the color of urine during malaria treatment?
- If I say to you now, "you are going to take the malaria medicine, but if your urine gets dark like Coke you should go straight to the health unit" What would this dark urine mean to you?
- Does this urine represent what I said? [SHOW PICTURES OF REAL CASES].

TESTING OF EDUCATIONAL MATERIALS

The focus of this section is to evaluate the different visual and message elements of the instructional materials, comparing the character-based and Icon-based versions. The interviewer shall give the participant(s) **RED** and **GREEN** markers and instruct them to return to the version of the LEAFLET issued for the previous activity. Circle in **RED** anything that the parent/guardian considers confusing and/or unpleasant for any reason and b) Circle in **GREEN**

anything that the Parent/Guardian finds clear, easy to understand/follow and/or pleasant for any reason.

[For **GREEN** circled areas, ask:]

1. In what ways is it easy to understand/pleasant?
2. What specifically do you like in this part? Why?
3. How can this be improved, how can it be more clear/attractive to you?

CARD

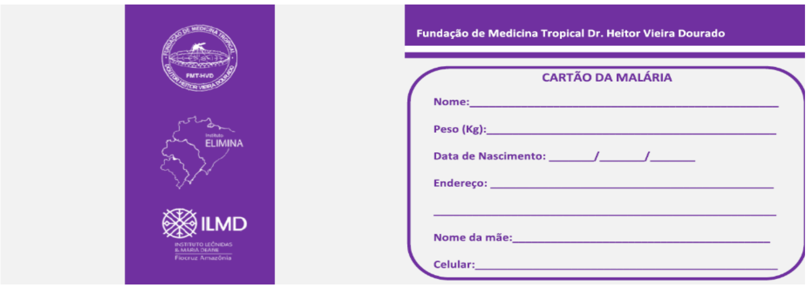

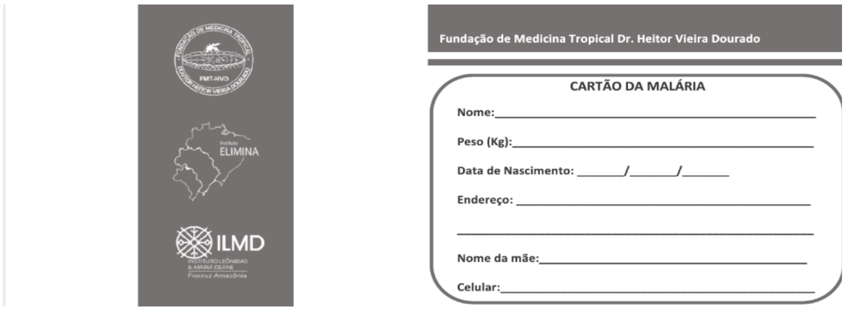

- **CLOSURE**

Those were all the questions I had for you. Thank you very much for your time and participation. Do you have any questions for me?
